# Beyond Platonic: How to Build Metal–Organic Polyhedra Capable of Binding Low-Symmetry, Information-Rich Molecular Cargoes

**DOI:** 10.1021/acs.chemrev.1c00763

**Published:** 2022-04-18

**Authors:** Charlie T. McTernan, Jack A. Davies, Jonathan R. Nitschke

**Affiliations:** Yusuf Hamied Department of Chemistry, University of Cambridge, Lensfield Road, Cambridge CB2 1EW, United Kingdom

## Abstract

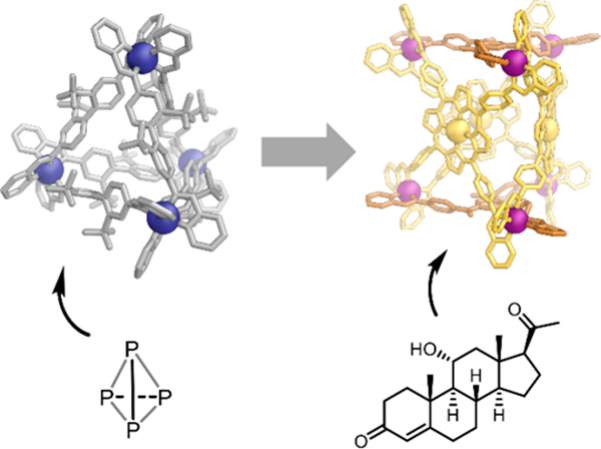

The field of metallosupramolecular chemistry has advanced rapidly in recent years. Much work in this area has focused on the formation of hollow self-assembled metal-organic architectures and exploration of the applications of their confined nanospaces. These discrete, soluble structures incorporate metal ions as ‘glue’ to link organic ligands together into polyhedra.Most of the architectures employed thus far have been highly symmetrical, as these have been the easiest to prepare. Such high-symmetry structures contain pseudospherical cavities, and so typically bind roughly spherical guests. Biomolecules and high-value synthetic compounds are rarely isotropic, highly-symmetrical species. To bind, sense, separate, and transform such substrates, new, lower-symmetry, metal-organic cages are needed. Herein we summarize recent approaches, which taken together form the first draft of a handbook for the design of higher-complexity, lower-symmetry, self-assembled metal-organic architectures.

## 1 Introduction

### 1.1 Overview

**Figure 1:**
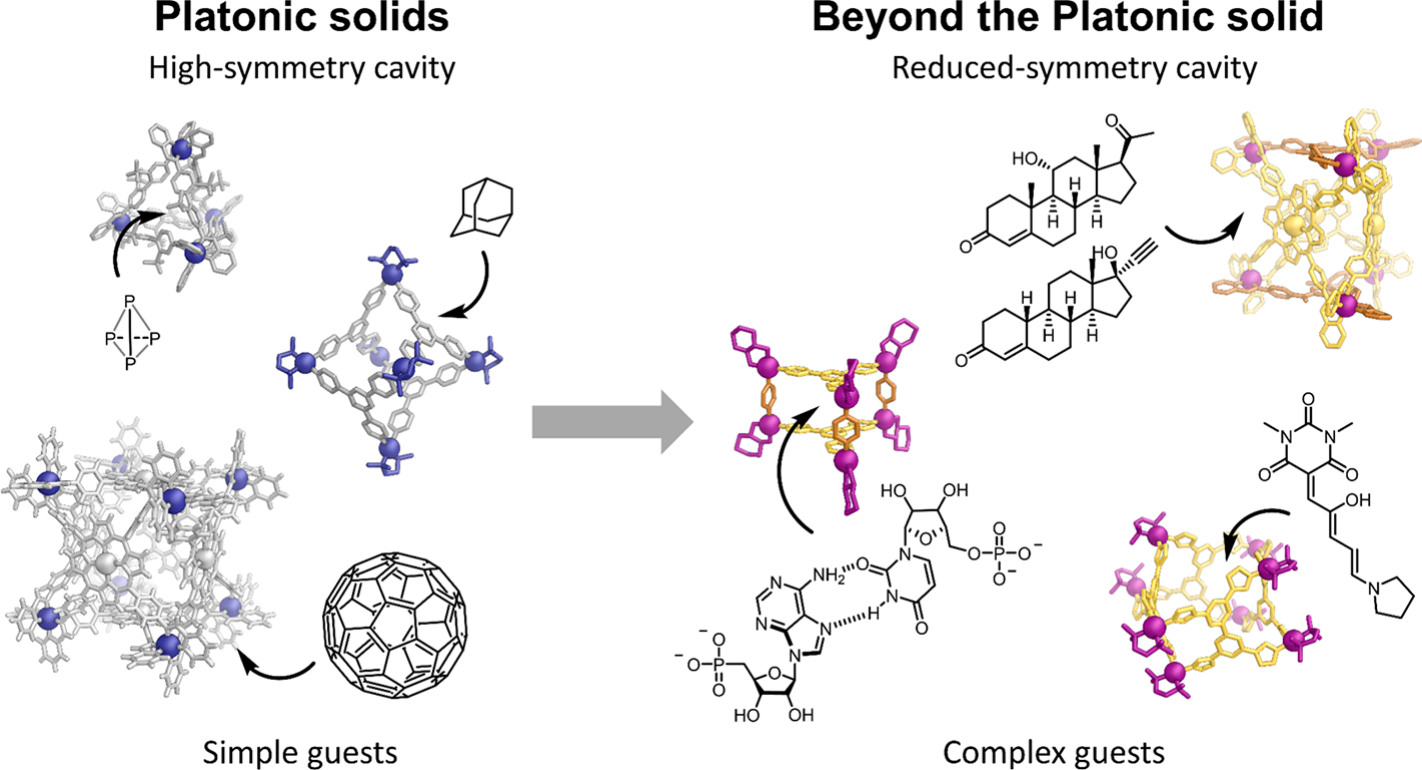
Examples of coordination cages with structures corresponding to Platonic solids, which are well-adapted to pseudospherical guests, contrasted with more complex “beyond Platonic” cages, which are primed for binding of anisotropic guests.^25,35,36,59,62−64^

The field of metallosupramolecular chemistry has advanced rapidly in recent years. Much work in this area has focused on the formation of hollow self-assembled metal–organic architectures and exploration of the applications of their confined nanospaces.^1−5^ These discrete, soluble structures incorporate metal ions as “glue” to link organic ligands together into polyhedra. Their hollows have found applications in binding and sensing guests,^6−8^ stabilizing reactive molecules,^9−13^ and catalyzing reactions as enzymes do.^14−19^ Most of the architectures employed to date have been highly symmetrical, as these have been the easiest to prepare (Figure 1).^20^ An understanding of the design principles underpinning the formation of high-symmetry metal–organic cages,^1^ such as tetrahedra,^21−24^ cubes,^25−28^ and octahedra,^29−34^ has enabled their synthesis and application.^35−38^ Modification of these structures, either before or after assembly,^39−41^ can imbue them with new functions.^42^ Such functions include modulation of the guest-binding properties,^43−46^ phase transfer (whereby a capsule and its cargo are induced to move between phases),^47,48^ and enabling the formation of higher-order metal–organic cage-based materials.^49−56^

Such high-symmetry structures contain pseudospherical cavities and thus bind roughly spherical guests optimally,^25,35−38^ although asymmetric guests can also be encapsulated.^10,12,20,57−59^ In some cases more than one smaller guest is bound within a relatively large cavity,^57^ or the flexibility of a guest enables it to adopt a folded conformation with a complementary size and shape for the cage cavity.^18,59,60^

Biomolecules and high-value synthetic compounds are rarely isotropic, highly symmetrical species.^61^ To bind, sense, separate, and transform such substrates, new lower-symmetry metal–organic cages are needed. In response to this need, recent work has focused upon the construction of metal–organic cages with interior cavities of reduced symmetry.

Many early examples of lower-symmetry structures were discovered serendipitously. Only a limited number of structure types beyond the Platonic solids were prepared using established design principles. The great promise of lower-symmetry structures to bind lower-symmetry guests selectively (Figure 1) has motivated efforts to decipher the rules underpinning the formation of complex architectures.^62−64^ Herein we outline different approaches that taken together form the first draft of a handbook for the design of higher-complexity, lower-symmetry, self-assembled metal–organic architectures.

### 1.2 Classification of Approaches

The design of metal–organic architectures has been discussed in terms of the following four strategies: the directional-bonding approach,^1^ the symmetry-interaction approach,^65^ the molecular-paneling approach,^66^ and the weak-link approach.^67−69^ Each of these strategies has been employed to form metallomacrocycles or high-symmetry three-dimensional architectures, often with Platonic geometries. With careful consideration, these design strategies can also be employed to form lower-symmetry structures that deviate from the Platonic solids. However, in this review we have opted for a method of classification that deviates from the strategies noted above because approaches enabling the formation of more complex metal–organic assemblies have recently been established that do not neatly fall within these categories. We focus instead upon the properties of the building blocks along with reaction conditions. This organization lends itself to the aim of this review—to act as a preliminary guide for the further design of complex self-assembled architectures.

**Figure 2:**
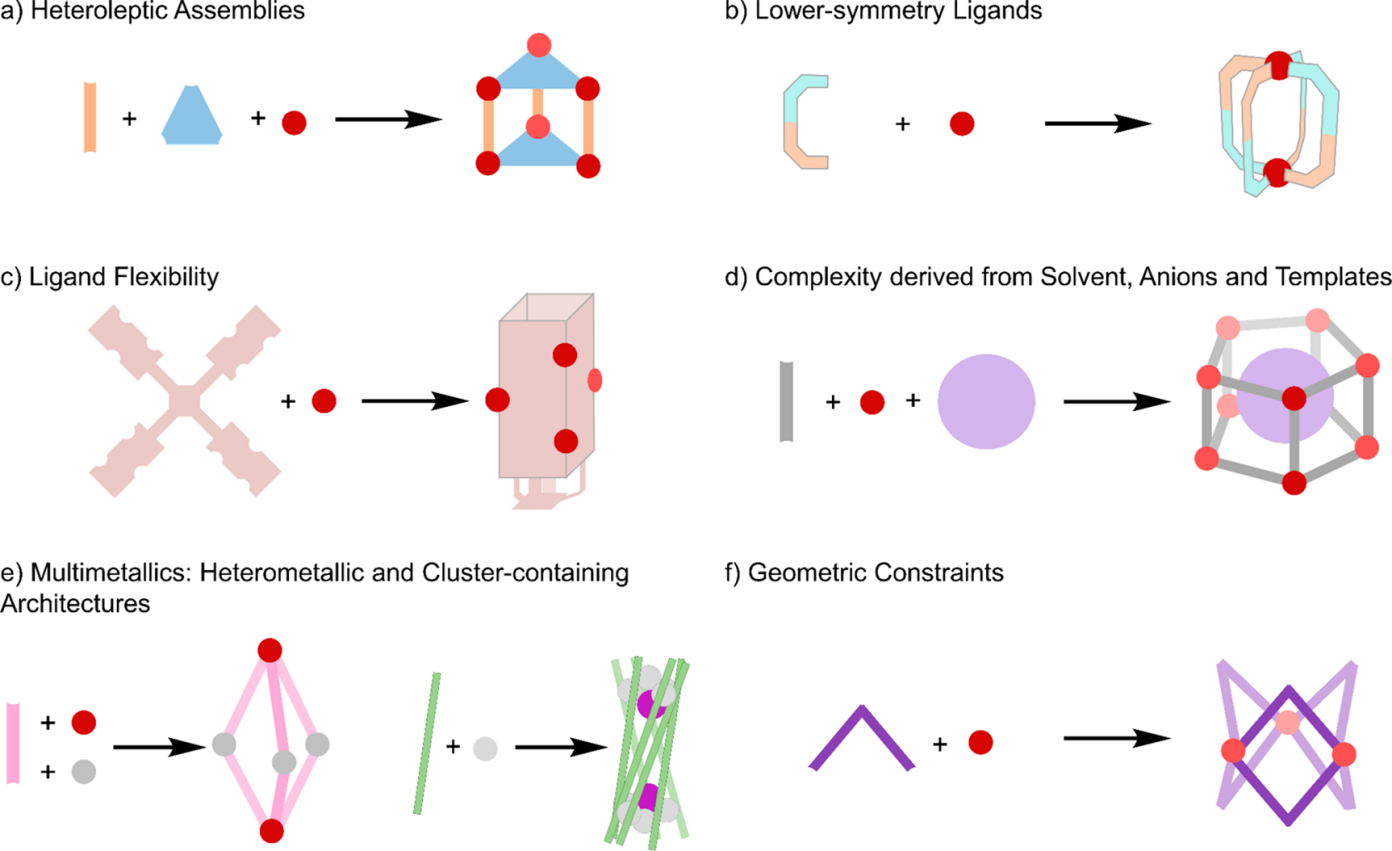
Categorization of approaches to forming complex metal–organic architectures.

Using this building block/reaction condition-based classification, we have identified six broad categories of approach (Figure 2): (1) Heteroleptic Assemblies; (2) Lower-Symmetry Ligands; (3) Ligand Flexibility; (4) Complexity Derived from Solvent, Anions, and Templates; (5) Multimetallics: Heterometallic and Cluster-Containing Architectures; and (6) Geometric Constraints.

Heteroleptic architectures incorporate multiple different ligands (Figure 2a). A particular challenge in this approach is to ensure that the different building blocks integrate into a single product rather than segregating to form simpler structures, each containing only one type of building block. One strategy developed to overcome this challenge involves harnessing the enthalpic and entropic driving forces that govern self-assembly in order to favor a heteroleptic structure.

A similarly intuitive approach involves the use of ligands with greater structural complexity or reduced symmetry, which then translates to the assembly of more complex three-dimensional architectures (Figure 2b).

Flexibility is often incorporated within ligands by the addition of alkyl spacers. Such enhanced flexibility can increase the array of feasible structures in comparison with the use of more rigid ligands, but it can also decrease the predictability of the self-assembly process (Figure 2c).

Complexity based upon solvent, anion, and template effects relies upon altering the self-assembly reaction conditions in order to favor structural complexity (Figure 2d). This method is well-established for producing complex metal–organic architectures. However, as with enhancing ligand flexibility, predicting the outcome of self-assembly using this approach can be challenging.

Multimetallic architectures either contain more than one type of metal center or have vertices that consist of homometallic clusters. Both cases can introduce coordinational flexibility, enabling the formation of architectures with increased structural complexity (Figure 2e).

The sixth approach to generating complex structures in a controlled and predictable manner is the incorporation of geometric constraints into the ligands. These geometric constraints can act to frustrate the formation of simpler structures, thus favoring the construction of architectures with greater complexity (Figure 2f). Examples in which steric control or non-covalent interactions are used to form complex metal–organic structures are also highlighted in this section.

### 1.3 Scope of the Review

This review focuses on techniques used to prepare metal–organic architectures by self-assembly of organic ligands and metal ions. Some complex structures that form with metal-cluster cores or with metal clusters as vertices^70−72^ are also included. The term “complex structure” within this review generally refers to structures that deviate from a framework corresponding to one of the high-symmetry Platonic or Archimedean solids. Some examples of structures that outwardly resemble these simple polyhedra, but with reduced symmetry, are included, particularly when the source of the reduced symmetry can be determined.

Although a key motivation for this review is to aid those who might wish to design new lower-symmetry structures for new applications, we focus on construction principles as opposed to the utility and functions of these structures. As the field that we attempt to cover is wide-ranging and fast-moving, omissions in our coverage will be inevitable. We apologize for these in advance.

## 2 Heteroleptic Assemblies: Incorporation of Multiple Ligands Generates More Complex Architectures

The complexity of metal–organic assemblies can be increased through the use of combinations of multiple ligands, particularly those having different topicities, i.e., with different numbers of metal-binding sites per ligand. In principle, combinations of multiple ligands with different shapes can allow the emergence of unusual architectures with complex geometries. In practice, however, achieving the selective formation of a single structure from a range of possibilities can be challenging. This section explores ways in which this challenge has been overcome, focusing on approaches that may allow general routes to heteroleptic structures.

### 2.1 Heteroleptic Selectivity by Destabilization of Homoleptic Assemblies

**Figure 3:**
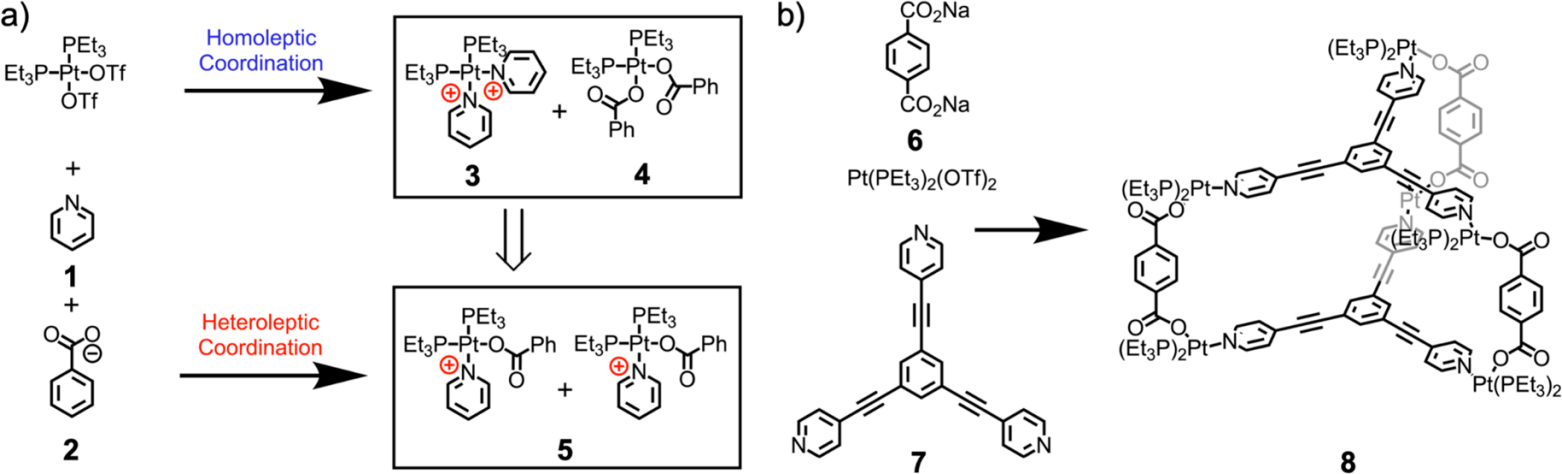
(a) Charge separation as a method for driving heteroleptic complex formation, leading to (b) selective formation of mixed-ligand cages.^73^

The selective assembly of a single heteroleptic metal–organic architecture is often entropically disfavored. For example, a square-planar metal vertex coordinated by 2 equiv of two different ligands through monodentate donors (i.e., ML^1^_2_L^2^_2_) may coexist with other mixed-ligand (i.e., ML^1^_1_L^2^_3_, ML^1^_3_L^2^_1_) and homoleptic (ML^1^_4_, ML^2^_4_) vertices. One way to overcome this tendency is to build in an enthalpic driving force for heteroleptic assembly. Stang et al. found that the principle of charge separation could drive the assembly of less-symmetric structures.^73^ This approach, shown in Figure 3, depends on the use of platinum(II) centers with two strong-field ligands in a *cis* configuration and both pyridine (**1**) and carboxylate (**2**) donor ligands. After coordination of a pyridine donor to platinum, the pyridine nitrogen atom bears a partial positive charge. When two pyridine donors are adjacent, they repel each other electrostatically (Figure 3, **3**). This repulsion is ameliorated when one of the pyridine donors is replaced by a carboxylate (Figure 3, **5**). This reduction in repulsion thus leads to the observed preference for heteroleptic coordination.

The Stang group has employed this concept extensively, for example to form an array of trigonal, tetragonal, and hexagonal prisms and other heteroleptic complexes, by combining *cis*-Pt^II^(PEt_3_)_2_(OTf)_2_ with 1,4-benzenedicarboxylate (**6**) (Figure 3) and three-, four-, or sixfold-symmetric pyridine donors.^73−75^Figure 3 shows the structures of **6,** threefold-symmetric donor **7**, and the self-assembly product **8**. In collaboration with the Huang group, this concept was extended to generate highly emissive platinum(II) metallacages using a fourfold-symmetric pyridine donor component that contains a fluorophore that undergoes aggregation-induced emission.^76^ The strict spatial separation enforced by the metal–organic architecture preserved the fluorescence in both high- and low-concentration regimes, allowing white-light emission. Similar principles were recently reported in a metallacycle where a high degree of intramolecular twist constrained the incorporated anthracenes, increasing the emission intensity.^77^ Furthermore, the same group, working with the Sun group, showed that metal–organic capsules can self-assemble into soft superstructures of up to the millimeter scale.^78^

Combinations of nitrogen-donor and carboxylate ligands have also been used to create molecular rectangles based on palladium.^79^ The formation of cages containing perylene diimide panels, which can bind polycyclic aromatic hydrocarbons, was recently reported by Zhang et al.^80^ By combining the orange emission of the cage and blue emission of a captured guest, white-light emission was obtained. Differences in fluorescence quantum yield between the solid-state and solution were also exploited to create hidden messages that were revealed upon exposure of the system to acetonitrile vapor.^80^

**Figure 4:**
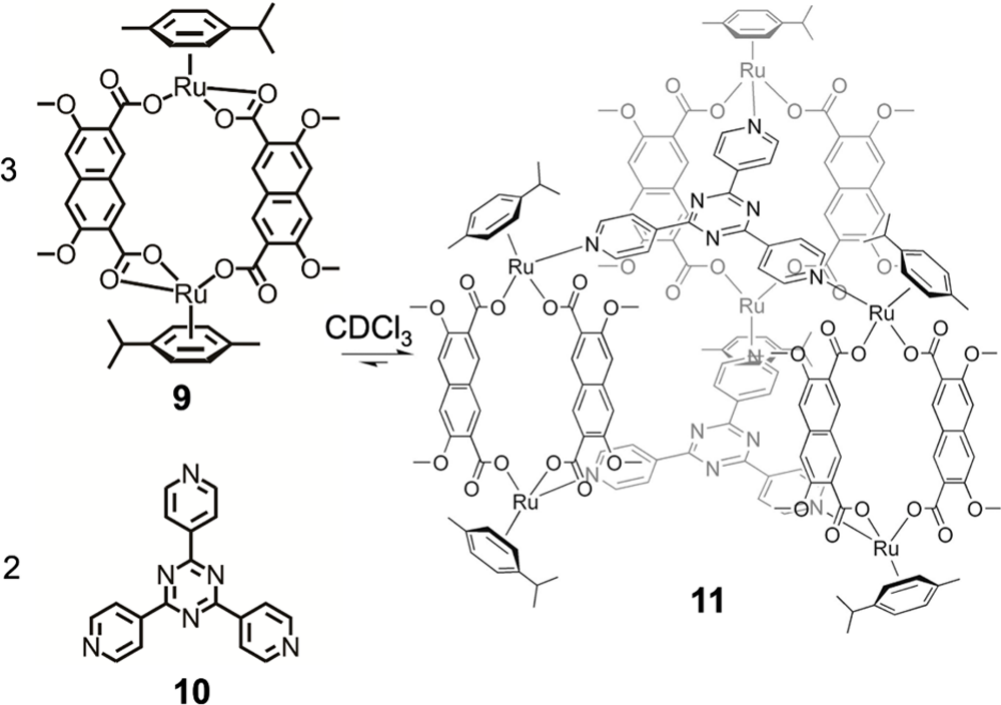
Selective assembly of trigonal prism **11** driven by the removal of a strained four-membered chelate ring in the homoleptic species. Reproduced from ref (81). Copyright 2010 American Chemical Society.

As shown in Figure 4, Severin et al. reported that strained homoleptic assemblies such as **9** rearrange following the addition of an extra ligand.^81^ Metallomacrocycle **9** is strained, and its strain is alleviated in heteroleptic assembly **11**, thus providing a driving force to counter the entropic cost of integrating more building blocks. In homoleptic assembly **9**, one carboxylate at each metal center forms a four-membered chelate ring, the strain of which is relieved as these carboxylates become monodentate in flexible trigonal prism **11** following the addition of 2,4,6-tris(pyridin-4-yl)-1,3,5-triazine (**10**). The resulting monodentate binding endows product **11** with a high degree of flexibility. In the absence of a guest, the trigonal-prismatic framework of **11** collapses in the solid state, forming a compressed structure without an interior cavity. However, when coronene is added, the trigonal prism expands to encapsulate two coronene guests in the solid state. This work shows that flexible coordination cage cavities can be generated not just from flexible organic ligands but also from coordinational flexibility about metal centers.

**Figure 5:**
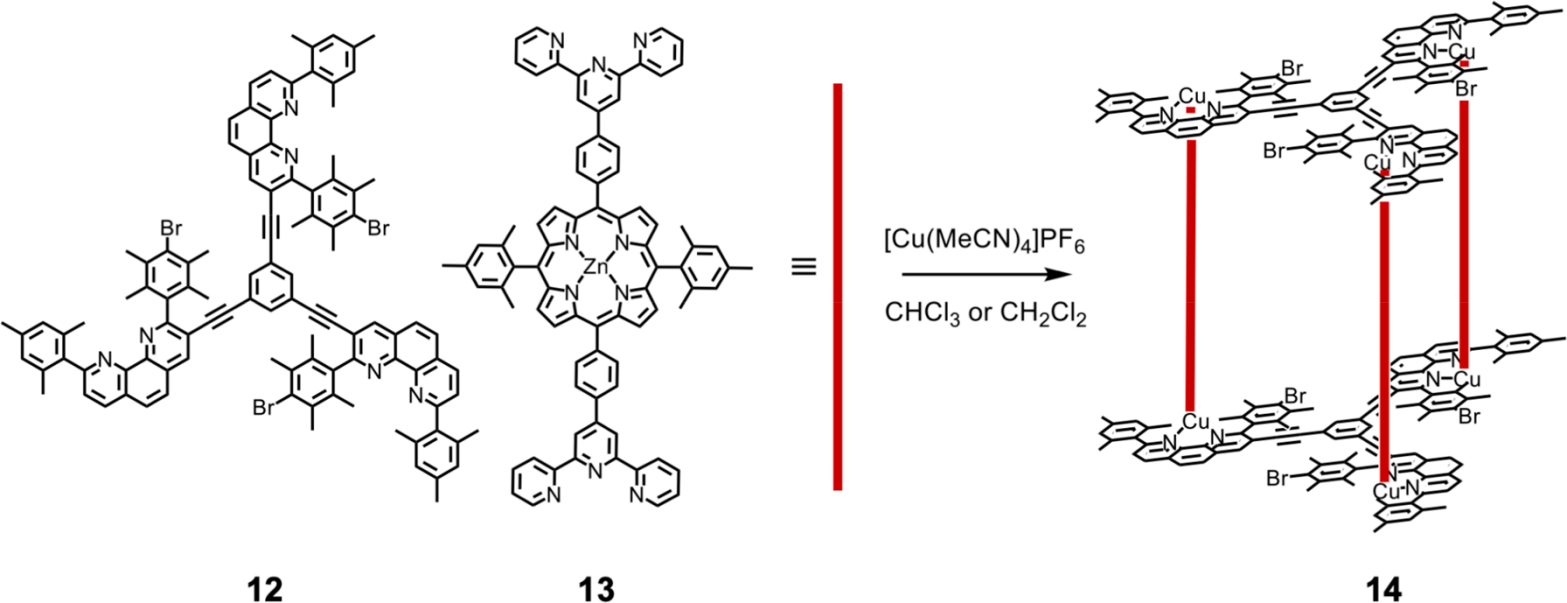
Selective assembly of heteroleptic trigonal prism **14** from precursors **12** and **13** driven by steric restriction involving hindered phenanthrolines (HETTAP).^82^

Schmittel and co-workers reported the application of their “heteroleptic terpyridine and phenanthroline metal complexes” (HETTAP) concept to generate myriad of self-assembled structures, including nanoprisms. This approach relies on steric hindrance around the phenanthroline units to prevent homoleptic assembly.^82,83^ By combining a threefold-symmetric bulky phenanthroline-based ligand (**12**) with linkers of different lengths (i.e., **13**, shown in Figure 5), the authors generated a series of trigonal prisms of differing heights of the general form Cu^I^_6_L^1^_2_L^2^_3_.

The heteroleptic architecture of **14** was further stabilized, eliminating minor byproducts, by the addition of a suitable bridging guest capable of coordinating between the zinc centers in the porphyrins of the ditopic ligands. A planar tridentate pyridine ligand that binds in the central belt of the three porphyrins drives the quantitative formation of the heteroleptic structure. Similar approaches, heteroleptic bis(phenanthroline) complexation (HETPHEN) and heteroleptic pyridine and phenanthroline complexation (HETPYP), have also been shown to selectively yield heteroleptic metal–organic complexes.^83^

**Figure 6:**
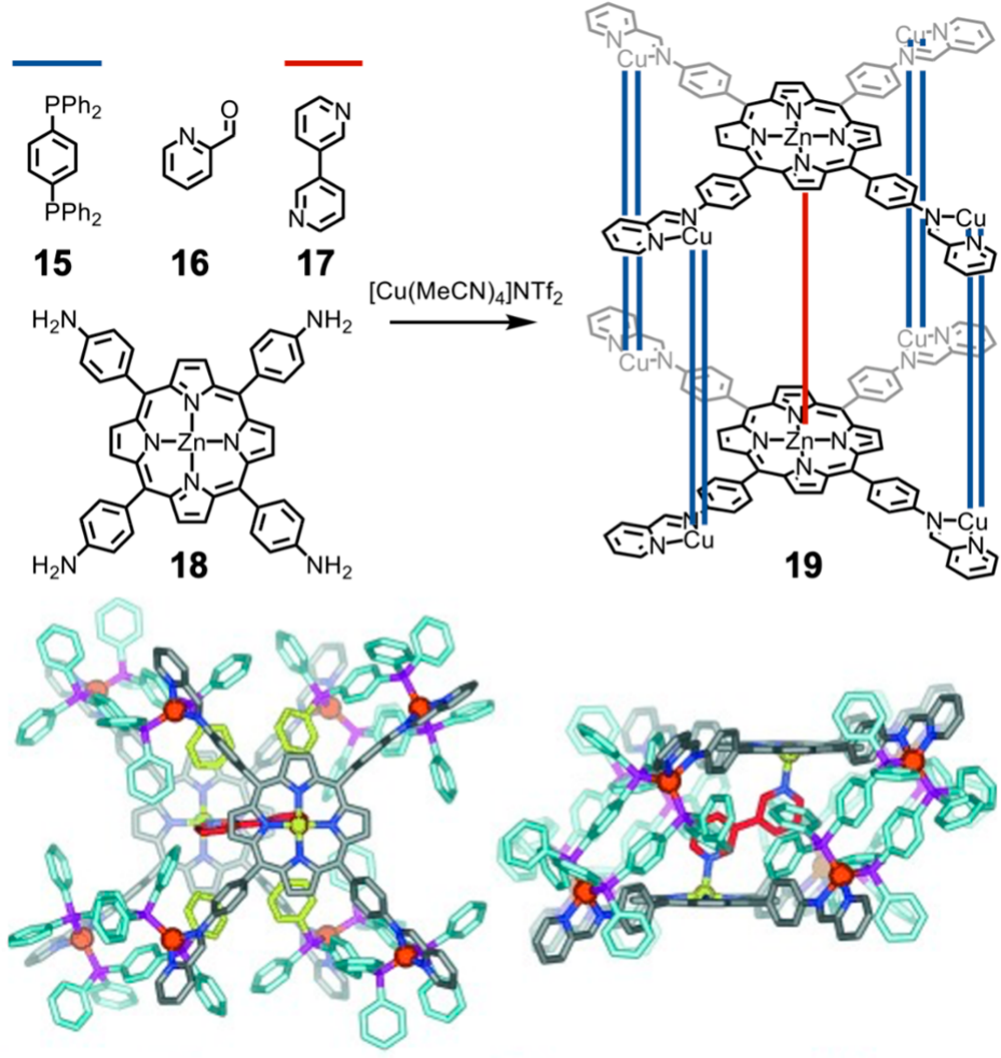
Formation of heteroleptic rhomboidal prism **19** by disfavoring the formation of homoleptic architectures. The offset between the zinc centers in the porphyrins leads to the selective incorporation of 3,3′-bipyridine (**17**). Adapted with permission from ref (87). Copyright 2015 Wiley-VCH Verlag GmbH & Co. KGaA, Weinheim.

A system may be guided toward heteroleptic assembly through destabilization of alternative homoleptic products that would undergo steric clash. An early seminal example was provided by Yoshizawa and co-workers, who combined sterically hindered and unhindered ligands containing two pyridines to form heteroleptic trigonal prisms.^84^ Similar approaches have been taken more recently by the Clever group, who used steric bulk to destabilize certain assemblies in order to favor heteroleptic species.^85,86^ We developed this concept during the selective formation of a copper(I) rhomboidal diporphyrin prism, shown in Figure 6.^87^ Upon mixing of 8 equiv of the bis(diphenylphosphino)benzene (**15**) struts, 8 equiv of 2-formylpyridine (**16**), a guest (**17**), and 2 equiv of the tetratopic zinc(II)porphyrin (**18**) with 8 equiv copper(I), rhomboidal prism **19** forms. The offset between the porphyrins within **19** leads to its selective binding of 3,3′-bipyridine (**17**) between zinc centers.

The formation of a homoleptic L_2_Cu^I^_4_ porphyrin copper(I) sandwich complex is disfavored by steric clashes between the phenyl groups, and the formation of copper(I) complexes involving the coordination of more than two phosphines is disfavored by the steric bulk of the phenyl groups on phosphorus. The simplest assembly that gives coordinatively saturated copper(I) is thus prism **19**. The preference for heteroleptic assembly is likely reinforced by the known preference for copper(I) to selectively form mixed phosphine–pyridine complexes.^88^

**Figure 7:**
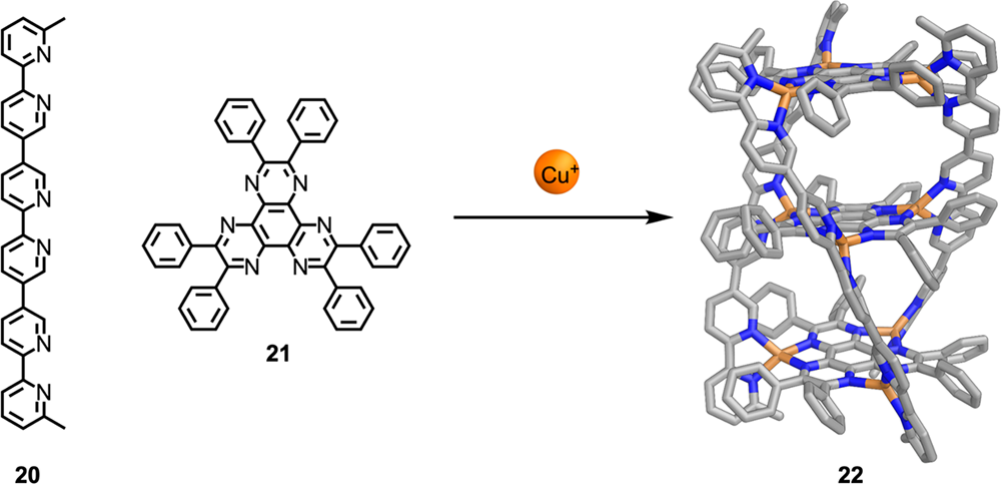
Formation of heteroleptic cylindrical complex **22**, with two guest-binding compartments, from tris(bipyridine) **20** and HAT **21**.^89,90^

The strategy of using ligands with donors of differing coordinative strength can also drive heteroleptic assembly in concert with the steric effects noted above. As shown in Figure 7, Lehn and co-workers reported a series of cylindrical complexes based on combinations of linear oligo(bipyridine) ligands such as **20** with planar, threefold-symmetric hexaazatriphenylene (HAT) ligand **21** and either silver(I) or copper(I).^89,90^ The electron-deficient HAT ligands bind less strongly than bipyridines, and their phenyl groups generate steric clashes when two HAT ligands bind around a single metal ion. Assemblies formed from HAT **21** alone would thus be relatively unstable as well as polymeric in nature and thus entropically less favored than the discrete cylindrical assemblies that are observed to form. Lehn et al. used linear ligands containing up to four bipyridine motifs, thus generating cylinders with up to three spatially separated binding pockets. Although the host–guest behavior of this system was not investigated in detail, the principle of using spatially separated binding pockets within the same assembly was further explored by others, such as Clever^91,92^ and Crowley^93^ (see Figure 63 in section 7.4).

### 2.2 Ligand Shape Complementarity

**Figure 8:**
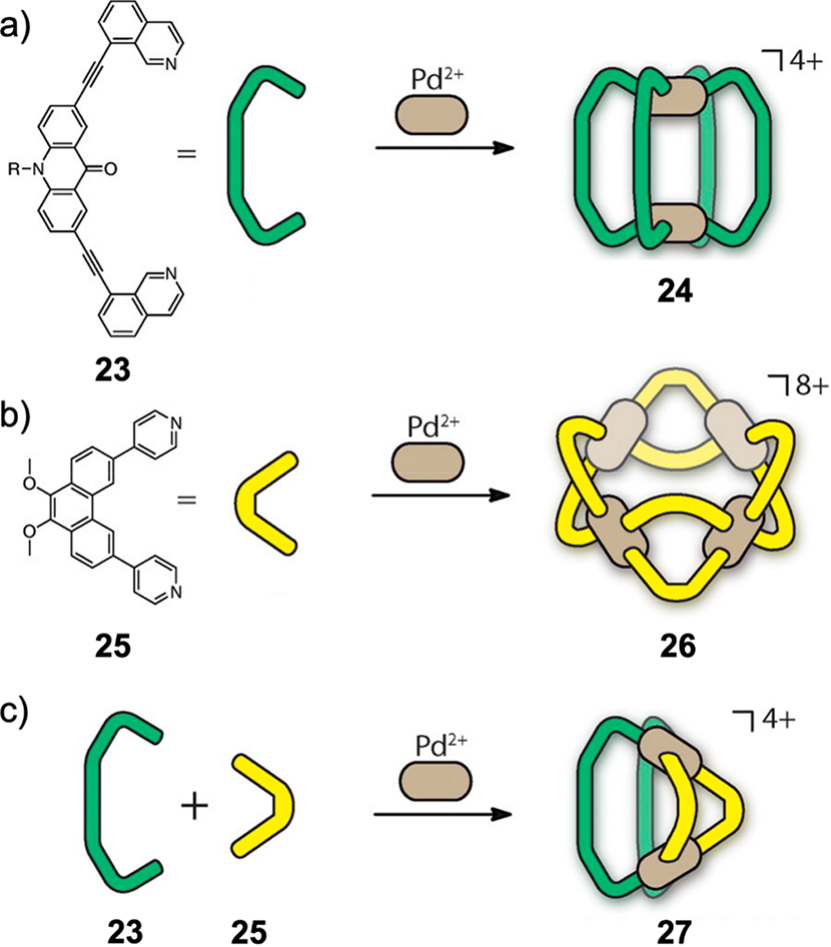
(a) Formation of homoleptic capsule **24**. (b) Formation of homoleptic capsule **26**. (c) Formation of heteroleptic lantern complex **27** driven by ligand shape complementarity between **23** and **25**. R = hexyl. Reproduced from ref (96). Copyright 2016 American Chemical Society.

Clever et al. reported a multitude of different heteroleptic Pd^II^_2_L_4_ lantern-type structures, based upon their initial work with analogous homoleptic structures, that contain bidentate ligands incorporating pyridine donors with parallel coordination vectors.^94,95^ Clever’s approach to forming heteroleptic structures exemplifies the use of shape complementarity.^96,97^ In the example in Figure 8, bidentate ligand **23** contains isoquinoline donors, and another, **25**, contains pyridine donors, each with nonparallel coordination vectors.^96^ Strain is thus incorporated into homoleptic structures **24** and **26**, as the offset coordination vectors cannot close up into a polyhedron by coordinating to square-planar palladium(II) without distortion. When mixed, however, the two ligands come together to form Pd^II^_2_**23**_2_**25**_2_ heteroleptic architecture **27** in which each ligand is *cis* to its complementary partner, thus forming a tilted lantern architecture. The extension of this concept to a wider variety of ligands subsequently enabled the discovery of an unusual self-penetrating heteroleptic cage architecture.^98^ Clever and co-workers reported a range of interpenetrated and heteroleptic systems based on similar principles.^99−103^ Severin and co-workers recently reported the use of similar “banana-shaped” ligands to create heteroleptic cages based on a virtual combinatorial library involving six separate ligands. This led to the formation of a trigonal-antiprismatic [Pd^II^_6_L_6_L′_6_](BF_4_)_12_ structure.^104^

**Figure 9:**
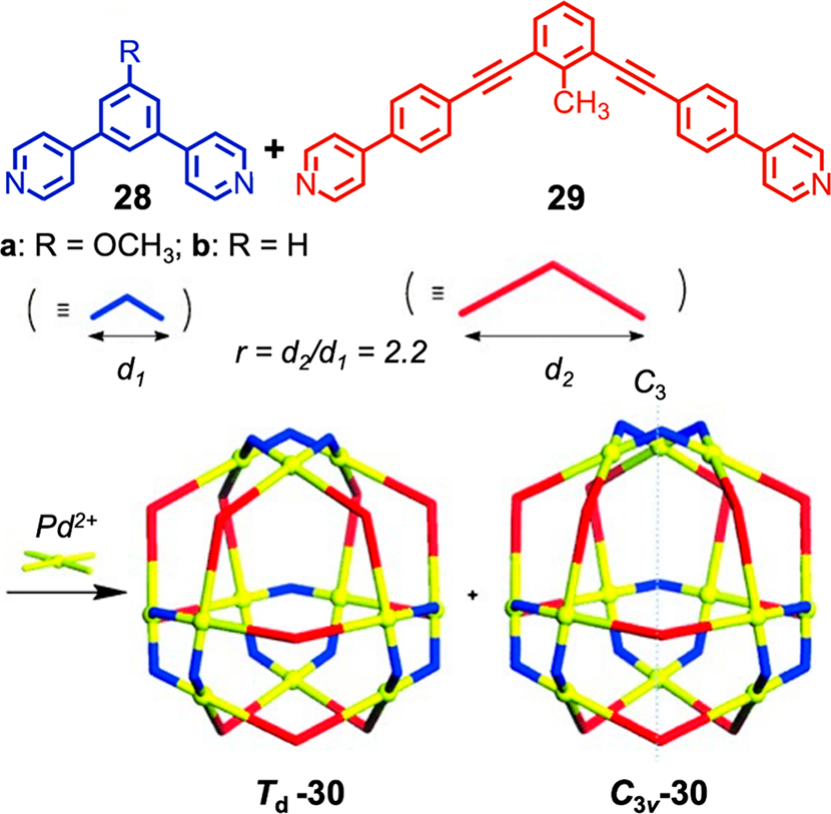
Formation of two diastereomers of heteroleptic cantellated tetrahedron **30** from two ligands, **28** and **29**. Adapted with permission from ref (105). Copyright 2014 Wiley-VCH Verlag GmbH & Co. KGaA, Weinheim.

The Fujita group reported the assembly of a heteroleptic cantellated tetrahedron from ligands **28** and **29** (Figure 9).^105^ These ligands have the same angle between coordinating groups but different lengths. Each ligand forms a Pd^II^_12_L_24_ cuboctahedral assembly when combined with Pd^II^ on its own. However, when combined in a 1:1 ratio, the two diastereomers of product **30** shown in Figure 9 form instead. Rather than narcissistic self-sorting, where each homoleptic assembly forms independently, or random mixing, where a collection of different assemblies form with different ratios of the two ligands incorporated, the system instead produces only Pd^II^_12_**28**_12_**29**_12_ assemblies. Each *cis* pair of ligands coordinating the same Pd^II^ forms part of a smaller Pd^II^_3_**28**_3_ or larger Pd^II^_3_**29**_3_ triangular metallomacrocyle, with four of each of these metallomacrocycles covering the cage surface, sharing edges with Pd^II^_4_**28**_2_**29**_2_ rectangles. The Pd^II^_12_**28**_12_**29**_12_ constitution of **30**, as opposed to other ratios of **28** to **29**, thus minimizes strain among these triangles and rectangles.

Similar principles were used by Benkhäuser and Lützen to create a dinuclear copper(I) molecular kite from subcomponents that did not assemble into discrete, unstrained structures individually.^106^

### 2.3 Entropy as a Driving Force for Heteroleptic Assembly

**Figure 10:**
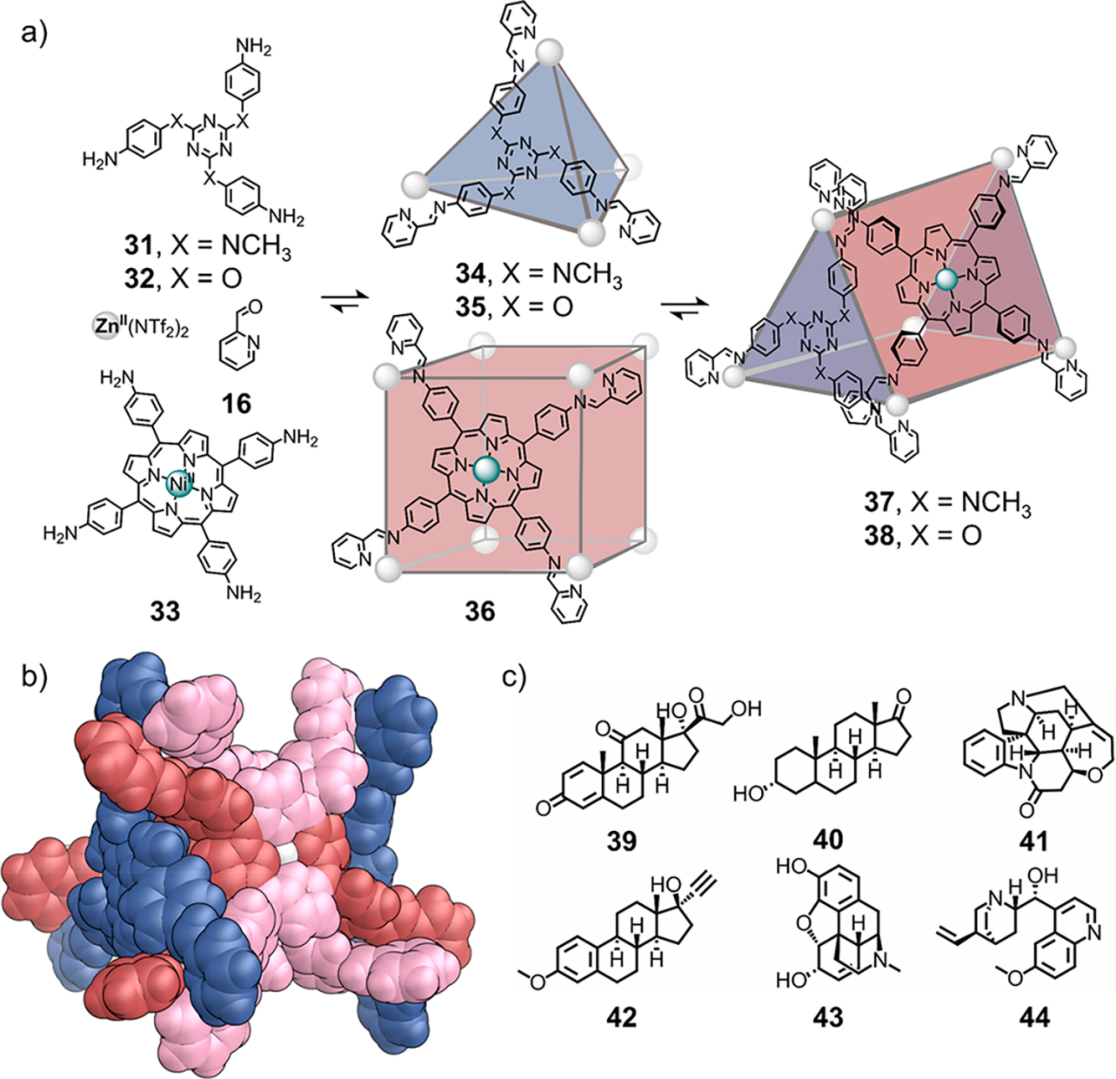
Formation of entropically favored heteroleptic triangular-prismatic complexes that can bind biologically relevant molecules. (a) Assembly of heteroleptic architectures **37** and **38**. (b) Crystal structure of **37**. (c) Pharmaceutical guests bound by the heteroleptic assemblies. Adapted from ref (62). Copyright 2019 American Chemical Society.

We recently reported a system that undergoes heteroleptic assembly by entropically favoring the mixed architecture (Figure 10).^62^ Cubes **36** and tetrahedra **34** or **35** are in equilibrium with triangular prisms **37** or **38**, respectively. The triangular-prismatic architecture is disfavored enthalpically, but its formation is favored entropically for two reasons. First, the triangular prism has a greater number of conformational microstates: each porphyrin unit adopts a saddled configuration, bowing in or out, in the triangular prism, whereas the porphyrins must lie planar in the cube. Second, the combined cavity volume of triangular prisms **37** or **38** is smaller than the combined volumes of the corresponding cubes and tetrahedra. Thus, fewer solvent molecules are trapped inside the cavities of triangular prisms **37** or **38** relative to the tetrahedra (**34** or **35**) and cube (**36**), leading to a more favorable entropy.

Homoleptic structures, such as **34**, **35**, and **36**, have higher symmetry and more-spherical cavities than the corresponding heteroleptic structures **37** and **38**. Such spherical, isotropic cavities are poorly adapted to the binding of more complex, anisotropic molecules of biological interest. A key advantage of the less-symmetric heteroleptic architectures **37** and **38** is the ability to bind higher-value, more complex substrates (e.g., **39**–**44**; Figure 10c) than the more symmetric homoleptic structures.

### 2.4 Favorable Interactions between Ligands to Drive Heteroleptic Assembly

**Figure 11:**
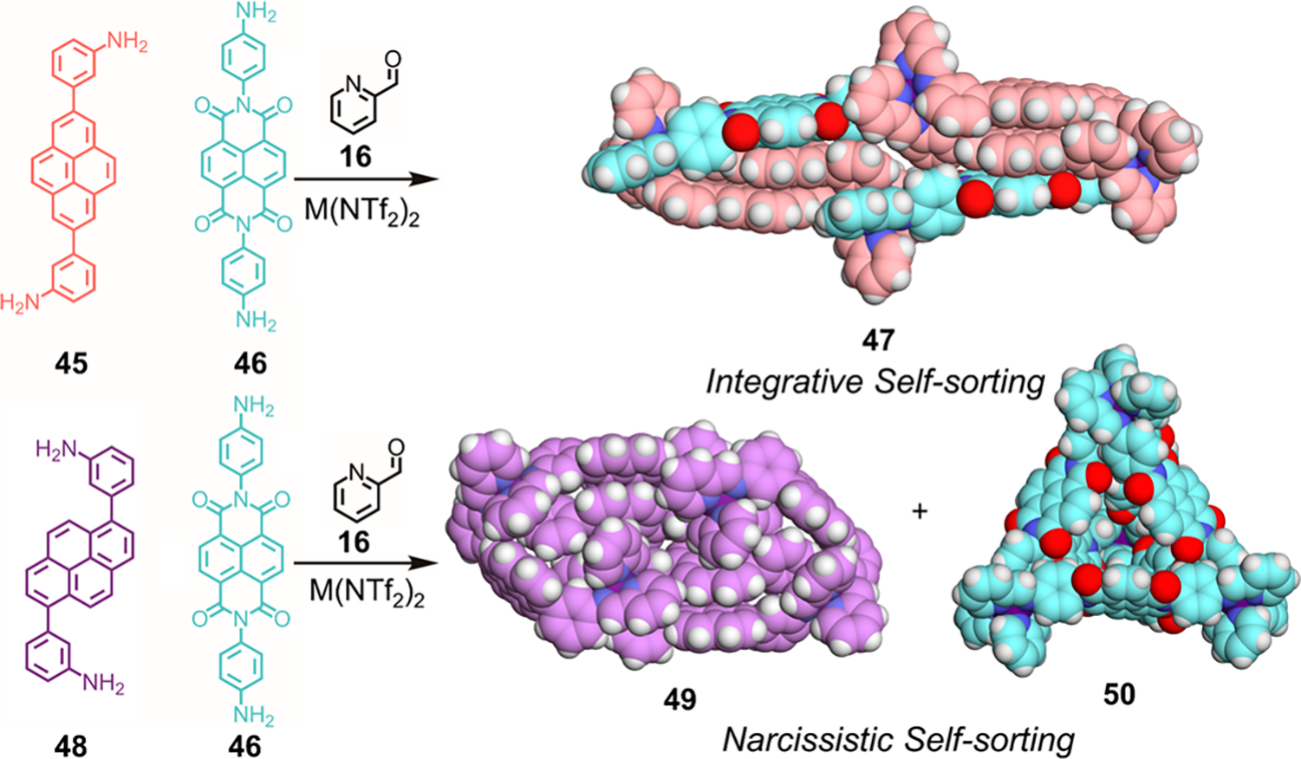
Formation of heteroleptic complex **47**, favored by aromatic stacking interactions, from the interplay of more electron-rich **45** and more electron-poor **46**, and narcissistic self-sorting observed from the combination of **48** and **46** to form homoleptic assemblies **49** and **50**.^107^

Heteroleptic assembly can be favored by engineering of additional favorable interactions that are not present in the corresponding homoleptic systems. We reported a system of mixed pyrene- and naphthalenediimide-based pyridylimine ligands (Figure 11).^107^ Alone, each ligand forms a stable homoleptic structure. However, together subcomponents **45** and **46** form Fe^II^_4_**45**_4_**46**_2_ elongated structure **47**, which has a different connectivity than either of the homoleptic assemblies. Differentially substituted subcomponent **48**, when combined with **46**, forms the original homoleptic architectures in an example of narcissistic self-sorting. The selective formation of heteroleptic structure **47** is driven by favorable aromatic stacking interactions between electron-rich and electron-deficient aromatic units that exist only in the mixed architecture. This stacking drives the assembly of the mixed architecture even in the presence of a guest that binds to only one of the possible homoleptic species. This system shows the importance of aromatic stacking interactions in metal–organic architectures.

Such stacking interactions were also critical in driving the formation of a recently reported twisted trigonal-prismatic architecture.^108^ Jung and co-workers also reported a catenated architecture based on the stacking of electron-deficient and electron-rich aromatic rings.^109^ In a similar vein, Yuasa et al. demonstrated that favorable interligand charge-transfer interactions can cause a preference for heteroleptic assemblies over homoleptic alternatives.^110^

Fujita and co-workers developed a heteroleptic Pt^II^_6_L_2_L′_3_ trigonal prism whose formation is templated by a rigid, flat aromatic guest that binds only in the heteroleptic architecture. Guest binding thus drives selective formation of the heteroleptic trigonal prism. After formation, the guest can be removed by extraction with an apolar solvent, leaving the empty trigonal prism.^111^ The cavity thus formed can then be used to stabilize the pairing of DNA nucleobases in aqueous solution.^63^

### 2.5 Complementary Binding Sites

**Figure 12:**
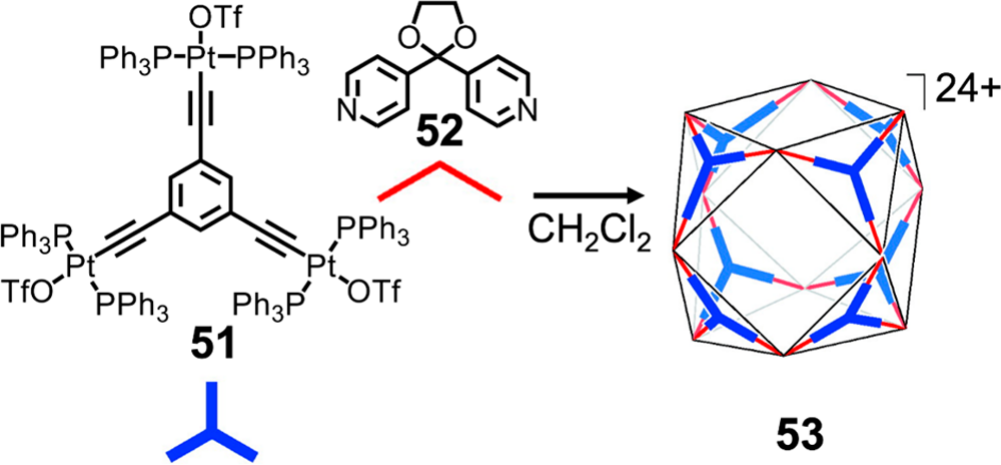
Formation of heteroleptic cuboctahedron **53** driven by the complementarity of binding sites of the different components. Adapted from ref (1). Copyright 2011 American Chemical Society.

Stang and co-workers have made extensive use of the square-planar geometric preference of palladium(II) and platinum(II) centers to construct metal–organic assemblies.^112^ They have obtained heteroleptic assemblies using the concept of complementary binding sites, whereby each component is unable to self-assemble without a complementary partner. As shown in Figure 12, cuboctahedron **53** can be prepared by the assembly of threefold-symmetric, planar metalloligand **51** with bidentate pyridine donor **52**. As the metal centers are covalently integrated into one ligand, a second ligand is required for assembly into the nanometer-scale product **53**. This work was subsequently extended to form similar chiral adamantanoid cages that incorporate optically active building blocks.^113^

Similar principles were previously used by Bosnich and co-workers to selectively generate platinum(II)-based heteroleptic rectangles using terpyridine and monopyridine ligands.^114^ The Nabeshima^115^ and Yam^116^ groups also used this concept to create molecular rectangles, and the area of complementary ligand denticity has recently been reviewed.^117^ The advantages of combining different donor groups in the same system were further established by Mukherjee and co-workers, who formed open “swings” and “boats” by using pyridine donors in combination with imidazole donors.^118^ These structures can bind C_60_ and catalyze Knoevenagel condensations.^119^

**Figure 13:**
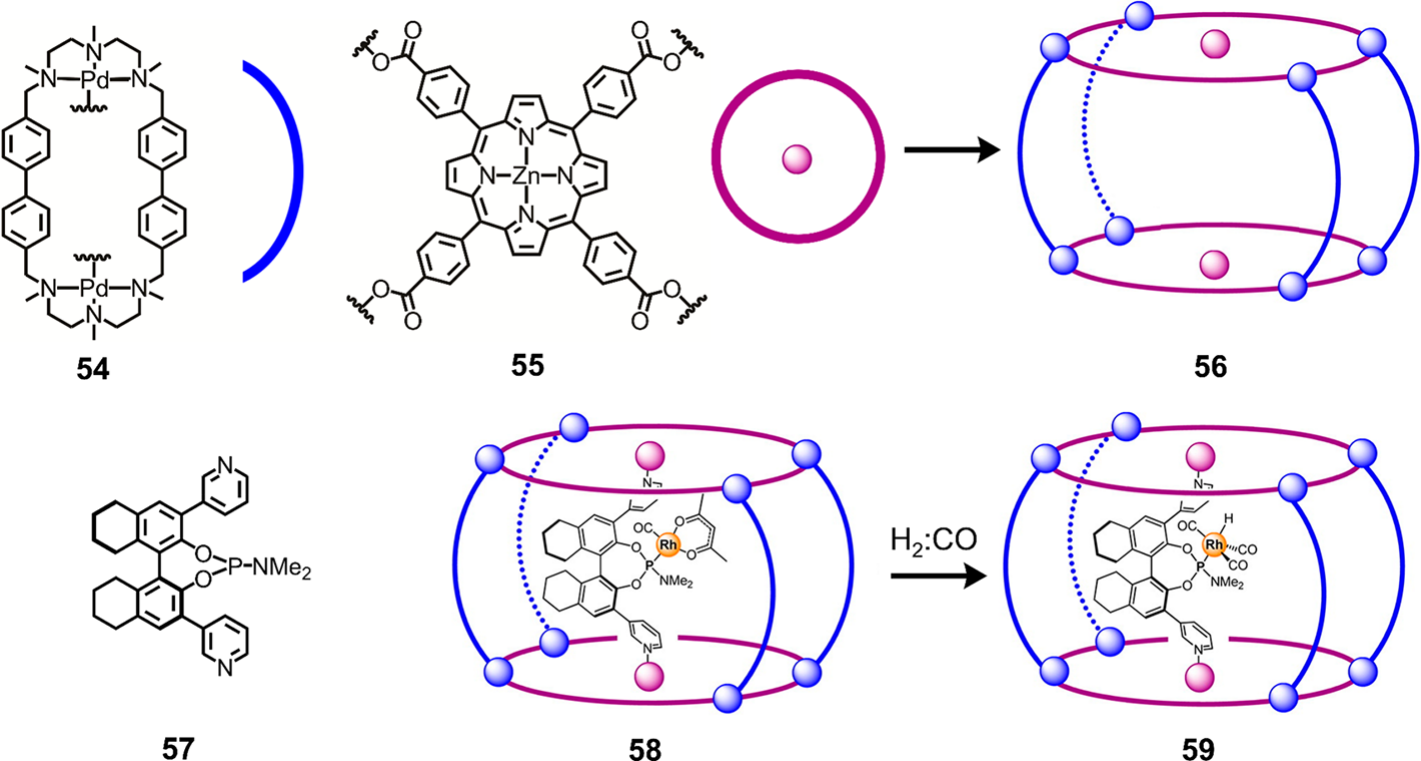
Formation of heteroleptic tetragonal prism **56** driven by the coordination complementarity of ligands **54** and **55**. Cage **56** binds aminophosphite **57**, which then binds rhodium (**58**) to form catalytically active rhodium complex **59**. Adapted from ref (120). Copyright 2015 American Chemical Society.

Other groups have further developed the concepts described above to form heteroleptic cages with useful properties. For example, the groups of Ribas, Costas, and Reek reported the formation of a tetragonal-prismatic supramolecular cage from the combination of tetratopic metalloporphyrin tetracarboxylate **55** and macrocycle **54** containing two palladium(II) centers, each coordinated by three nitrogen donors (Figure 13).^120^ In this system, the coordination preferences of Pd^II^ are satisfied by one carboxylate ligand and one macrocyclic ligand, leading to the formation of structure **56** with Pd^II^_8_**54**_4_**55**_2_ composition. This structure encapsulates aminophosphite ligand **57**, which coordinates rhodium to form **58**. The active supramolecular catalyst thus formed (**59**) operates with a greater degree of chiral induction due to cage control over the second coordination sphere. Similar capsules have been reported and used for the selective extraction and functionalization of fullerenes.^121−123^ In collaboration with the von Delius group, the Ribas group recently reported the formation of a “matryoshka” Russian doll-type assembly and its application in the selective formation of a single *trans*-3 fullerene bisadduct.^124^

**Figure 14:**
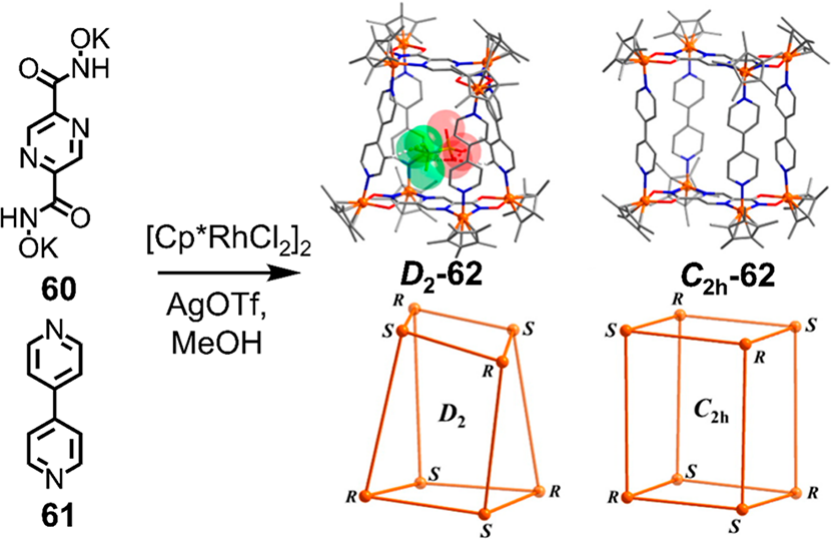
Assembly of molecular prisms with different symmetries based on the hard–soft bis(hydroxamate) donor **60** and 4,4′-bipyridine (**61**). Adapted from ref (125). Copyright 2015 American Chemical Society.

Jin and co-workers reported a system of heteroleptic cages where selective assembly is driven by the interplay between two pairs of distinct chelating sites, a harder O,O′ site and a softer N,N′ site, on a single hydroxamate ligand (**60**), as shown in Figure 14.^125^ Half-sandwich iridium and rhodium metal centers assemble with auxiliary pyridine-based ligands, such as 4,4′-bipyridine (**61**), to form tetragonal and trigonal prisms. The *D*_2_-symmetric diastereomer of cage **62** (Figure 14) binds triflate as a guest and template.

The hard/soft character of ligand **60** was also used to form heterometallic macrocycles with palladium and iridium centers selectively incorporated into the same framework. Within these heterometallic structures, palladium binds the softer nitrogen donors, whereas iridium binds the harder oxygen donors. One of these macrocycles encapsulated tetrathiafulvalene between parallel hydroxamate ligands.^125^ The authors recently reported an extension of this system in which symmetric bipyridine **61** is replaced by a bridging unit containing one pyridine and one carboxylate donor site, forming a *D*_2_-symmetric heteroleptic species selectively.^126^

**Figure 15:**
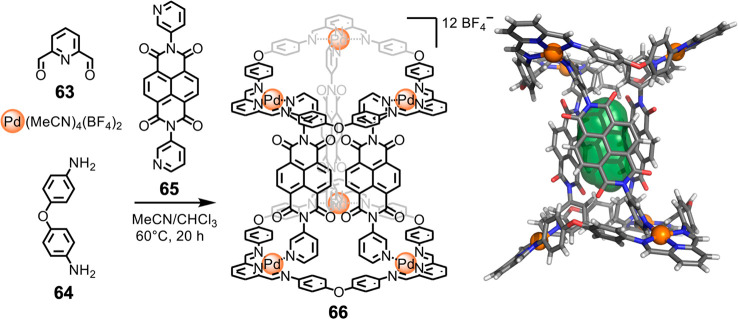
Formation of complex assembly **66** from subcomponents **63** and **64**, Pd^II^(MeCN)_4_(BF_4_)_2_, and *N*,*N*′-dipyridylnaphthalenediimide **65**. Reproduced from ref (127). Copyright 2019 American Chemical Society.

We reported a system of Pd^II^-based macrocycles and cages whose assembly is controlled by the addition of appropriate pyridine-containing templates to the assembled Pd^II^-bound macrocycles. Each Pd^II^ is coordinated by three nitrogens from the macrocycle (Figure 15) and one from the bridging ligand.^127^ The subcomponents 2,6-diformylpyridine (**63**) and flexible dianiline **64** assemble around palladium(II) templates to generate metal–organic macrocycles containing either three or four Pd^II^ centers, depending on the tri- or tetratopic nature of the pyridine template used. When we employed linear, ditopic pyridine template **65**, which has a geometry ill-adapted to incorporation within a single macrocycle, three-dimensional capsule **66** was generated. This structure (Figure 15) includes a trimeric macrocycle at each end with bridging ligands **65** between them. Assembly **66** forms cooperatively, with no structures observed containing fewer than three bridging ligands. Structure **66** encloses a small cavity, which was found to bind tetrafluoroborate selectively.

Similar systems were extended to form truncated tetrahedra and other metal–organic cages by the use of a tritopic aniline ligand. The dynamic pyridylimine bonds formed during self-assembly could be cleanly reduced to form secondary amines, thus disabling the equilibration process and fixing the structures formed.

### 2.6 Kinetic Traps

**Figure 16:**
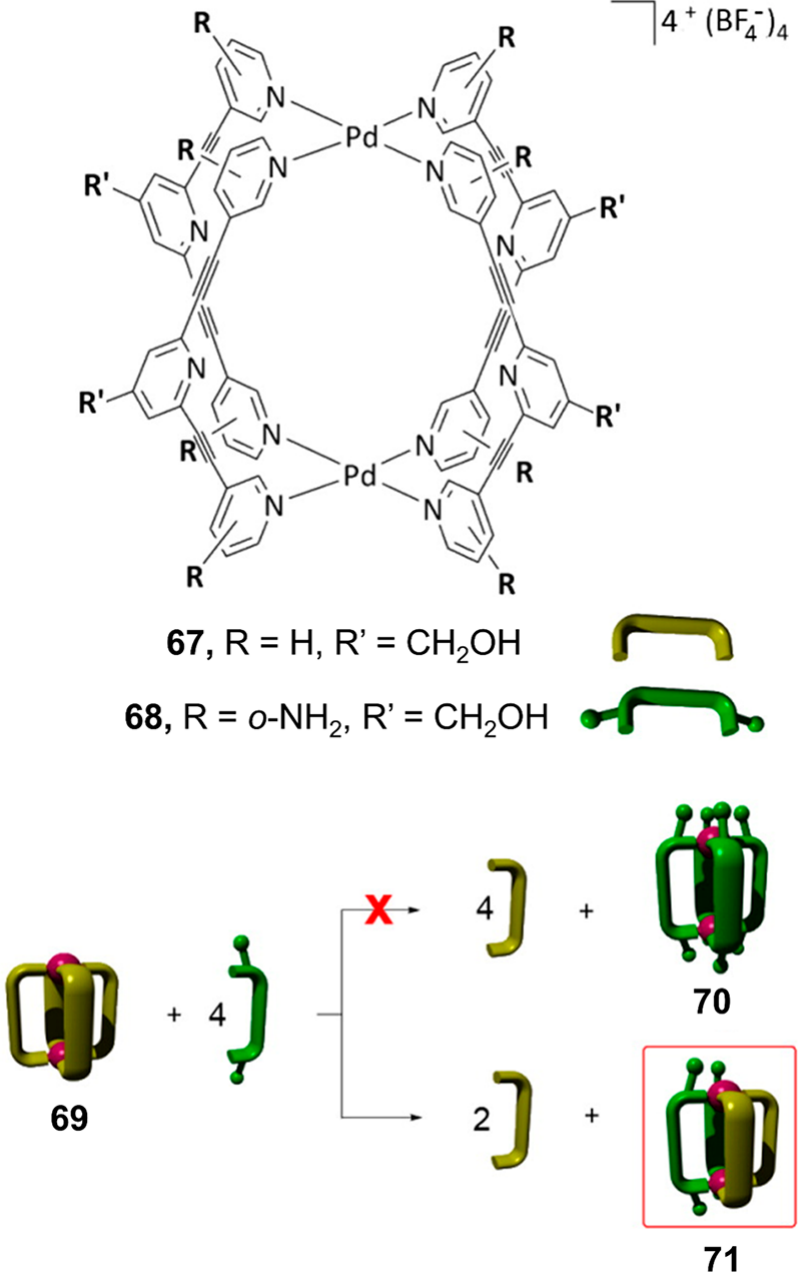
Formation of kinetically trapped heteroleptic molecular lantern complex **71** with selective incorporation of pairs of ligands **67** and **68**. Reproduced from ref (128). Copyright 2016 American Chemical Society.

Crowley and co-workers reported a novel approach to generating heteroleptic architectures that employs kinetic traps rather than favoring a thermodynamic product (Figure 16).^128^ Pd^II^_2_L_4_ lantern architecture **69**, formed from bidentate pyridine-containing ligand **67** with parallel coordination vectors (Figure 16), is combined with another ligand **68** containing 2-aminopyridines. Ligand **68** forms stronger bonds to palladium, so thermodynamics favors its incorporation. When excess ligand **68** is added to Pd^II^_2_**67**_4_ lantern **69**, Pd^II^_2_**67**_2_**68**_2_ lantern **71** forms selectively in a *cis* configuration. The selectivity for the *cis* isomer is attributed to hydrogen bonding between adjacent amino groups. The selective formation of a Pd^II^_2_**67**_2_**68**_2_ lantern, rather than complete substitution to form a homoleptic Pd^II^_2_**68**_4_ structure, is attributed to the effects of steric repulsion between the 2-amino groups and incoming pyridine ligands in the proposed associative mechanism. This repulsion increases the energetic barrier to ligand exchange, enabling the selective formation of the heteroleptic species. Calculations suggested that the heteroleptic species is a kinetically trapped metastable species rather than the thermodynamic product, and competition experiments supported this idea. Hydrogen bonding between the pyridine α-CH and adjacent 2-aminopyridine groups is inferred to reinforce this kinetic stability.

**Figure 17:**
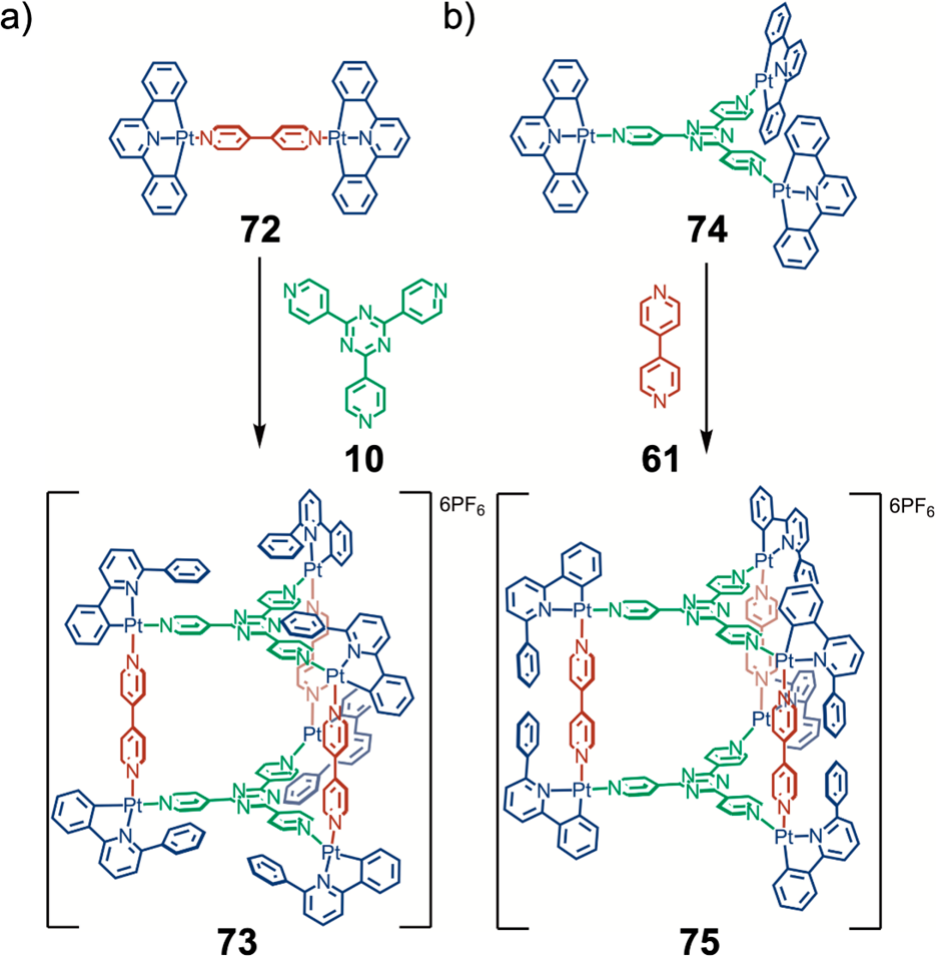
Selective formation of isomeric heteroleptic trigonal prisms **73** and **75** by control over the sequence of addition. (a) Initial addition of ditopic ligand **61**. (b) Initial addition of tritopic ligand **10**.^129^

An intriguing use of kinetic control in self-assembly was reported by Lusby, Barran, and co-workers, who used the low lability of cyclometalated platinum corners to create trigonal-prismatic assemblies (Figure 17).^129^ The identity of the product depends on the sequence of addition rather than the thermodynamic stability of the product. Starting from a platinum complex with one pyridine, one dimethyl sulfoxide, and two phenylato ligands, a bi- or terpyridine ligand is then added. This additional ligand displaces weakly bound dimethyl sulfoxide to form an intermediate complex with either twofold (**72**) or threefold (**74**) symmetry. In the case of twofold-symmetric intermediate **72**, tritopic pyridine ligand **10** is then added, which forms a new coordination bond *trans* to a phenylato ligand. This process displaces another phenylato ligand, which is then protonated. The phenyl group thus released is left above the threefold-symmetric face of trigonal prism **73**.

If instead bipyridine **61** is added to threefold-symmetric intermediate **74**, the released phenyl groups of product **75** instead stack above the twofold-symmetric ligand. This isomerism is further manifested in the mass spectrometry data collected, where the weaker coordination bonds *trans* to the phenylato group are observed to rupture preferentially. This approach provides an example of how the sequence of addition can control the outcome of a self-assembly process and thus provides a novel mode of generating structural complexity.

This section has reviewed different approaches for generating heteroleptic structures, which frequently have novel, lower-symmetry architectures. We have explored how control over both the entropy and enthalpy of formation can be used to bias systems toward thermodynamic heteroleptic assembly. More subtly, we have also seen how fine control of the balance of kinetics in a system can enable the formation of kinetically trapped heteroleptic products without preventing the error checking that is vital to the self-assembly of complex architectures.

## 3 Lower-Symmetry Ligands: Using Reduced-Symmetry Ligands Leads to Reduced-Symmetry Products

The complexity of metal–organic architectures may be increased through the use of components that themselves have more complex structures. This concept has recently been reviewed by Lewis and Crowley.^130^ Reduced-symmetry ligands can also lead to an increased number of possible structures. Thus, we also evaluate factors that drive the selective formation of one structure from among multiple possibilities.

### 3.1 Reduced-Symmetry Ligands

**Figure 18:**
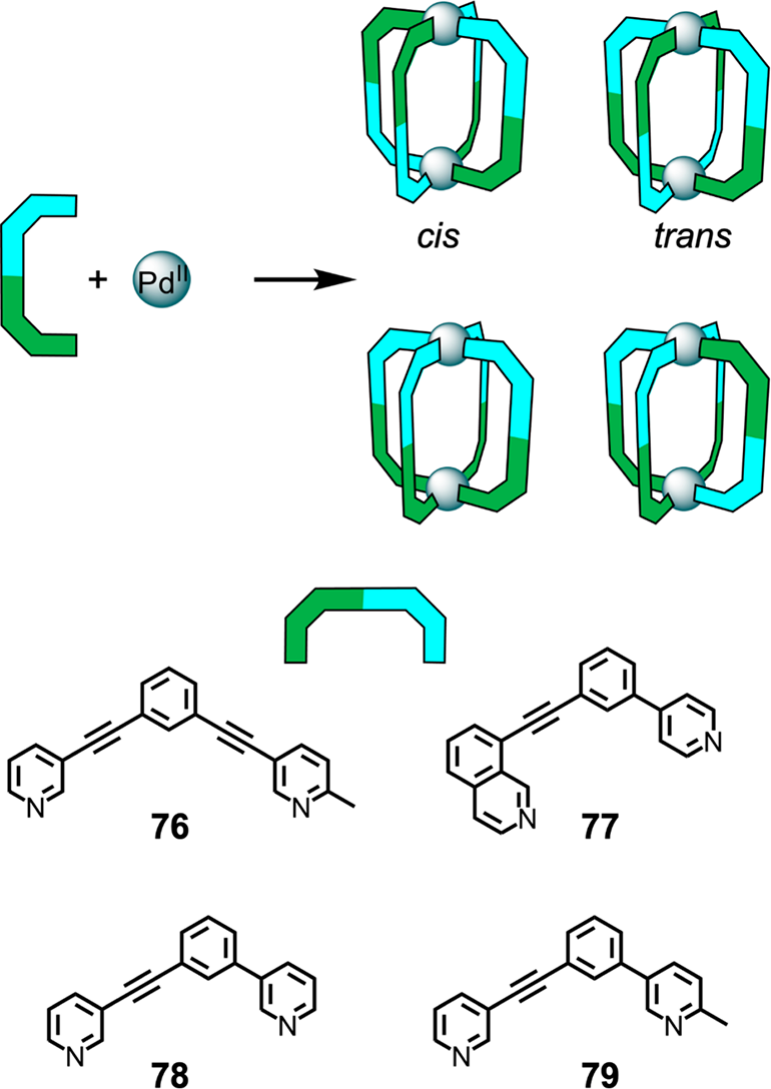
Representations of the four possible isomers of homoleptic Pd^II^_2_L_4_ cages that can be formed from one of the reduced-symmetry ditopic ligands **76**–**79**.^133^

M_2_L_4_ cages using bis-monodentate ligands and square-planar metal centers have been well-studied and would not be considered “complex” in terms of the scope of this review.^131,132^ However, several recent publications have reported the formation of M_2_L_4_ structures with reduced-symmetry ditopic ligands and a single type of metal ion^133−137^ or two different types of metal ion,^138^ leading to greater structural complexity. When M_2_L_4_ structures assemble from a reduced-symmetry ditopic ligand, several isomers are possible (Figure 18). Often one or more of these isomers are of lower energy than the others and therefore form preferentially.

Lewis and co-workers showed that the identity of the preferred isomer of a Pd^II^_2_L_4_ cage can be controlled by changing the identity of the ligand (**76**–**79**). Hindered ligand **76** produces a *C*_2*v*_-symmetric *trans*-Pd^II^_2_L_4_ isomer in MeCN, minimizing steric clashes, with product identification being supported by DFT calculations.^133^ Upon an increase in the polarity of the solvent by the use of DMSO, a mixture of the *trans*-Pd^II^_2_L_4_ and *cis*-Pd^II^_2_L_4_ isomers form. This phenomenon is tentatively attributed to selective stabilization of the *cis*-Pd^II^_2_L_4_ isomer by the more polar solvent, which is predicted by DFT to have a larger dipole moment than the trans isomer.

The *C*_2*h*_-symmetric *cis*-Pd_2_**77**_4_ isomer forms selectively in DMSO.^133^ This selectivity arises from the presence of different binding sites at the two ends of ligand **77**, a pyridine and an isoquinoline. Within **77**, the planes orthogonal to the coordinate vectors of the nitrogen donor atoms no longer coincide (even when the pyridine and isoquinoline rings are coplanar), thus favoring *cis*-Pd^II^_2_**77**_4_ formation. Subsequent investigations involving ligand **78** indicated that in this case the deviation from coplanarity was not significant enough to yield a single isomer of the Pd^II^_2_**78**_4_ complex. However, the greater steric hindrance around the coordination sphere of Pd^II^ bound to **79** results in the formation of a single Pd^II^_2_**79**_4_ isomer. On the basis of DFT calculations, *cis* stereochemistry was inferred.

Finally, the addition of steric bulk, in this case via the inclusion of methyl groups in **76** or **79**, causes an increase in the helical twist of the structure compared with analogous structures formed by ligands lacking methyl groups. The steric effects of these methyl groups on the conformation of the resulting structure may enable tailoring of the internal cavity space.

The Lewis group has also shown that reduced-symmetry ditopic ligands containing 1,2,3-triazole and isoquinoline binding sites can form a similar Pd^II^_2_L_4_ cage as a single *cis*-Pd^II^_2_L_4_ isomer.^137^ Variation of the substituent on the triazole moiety results in the formation of a series of externally functionalized cages. Because of the uniformity of the main ligand framework among all of the derivatized ligands, dynamic libraries of mixed-ligand cages are obtained when mixtures of the different ligands are used.

Bloch et al. recently demonstrated the use of conformational flexibility in producing reduced-symmetry ligands.^139^ In their system, a dicarboxylate ligand with a diimine core exists in three different rotational conformations, one of which has lower symmetry. Depending on the crystallization conditions, three distinct cage isomers are isolated from a dynamic library; their structures were determined by single-crystal X-ray crystallography. The three cage isomers each contained either two or four ligands in the reduced-symmetry conformation.

**Figure 19:**
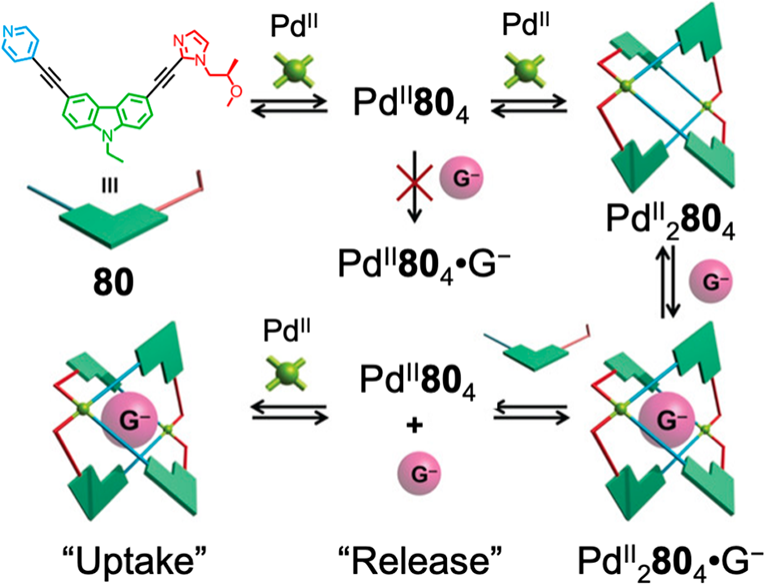
Stepwise self-assembly of a dynamic open Pd^II^_2_L_4_ coordination cage using unsymmetrical imidazole–pyridine-based ditopic ligand **80**. Stoichiometry-controlled structural transformation of this cage allows anion uptake and release. Adapted with permission from ref (134). Copyright 2019 Wiley-VCH Verlag GmbH & Co. KGaA, Weinheim.

Separate studies reported by Ogata and Yuasa^134^ and Crowley et al.^138^ also involved the formation of M_2_L_4_ structures with unsymmetrical ditopic ligands. Both utilized the differing labilities of coordination bonds involving different monodentate donors or metal ions to develop mechanisms for guest capture and release. Yuasa et al. altered the stoichiometric ratio of ligand to metal in the reaction mixture to drive the interconversion of a Pd^II^_2_**80**_4_ cage, capable of binding anions within its cavity, and a Pd^II^**80**_4_ complex, which does not bind guests (Figure 19). In this mononuclear complex, the imidazole groups of all four ligands are bound to the Pd^II^ center and the four pyridyl donors remain free because imidazole is a stronger donor than pyridine.^134^

**Figure 20:**
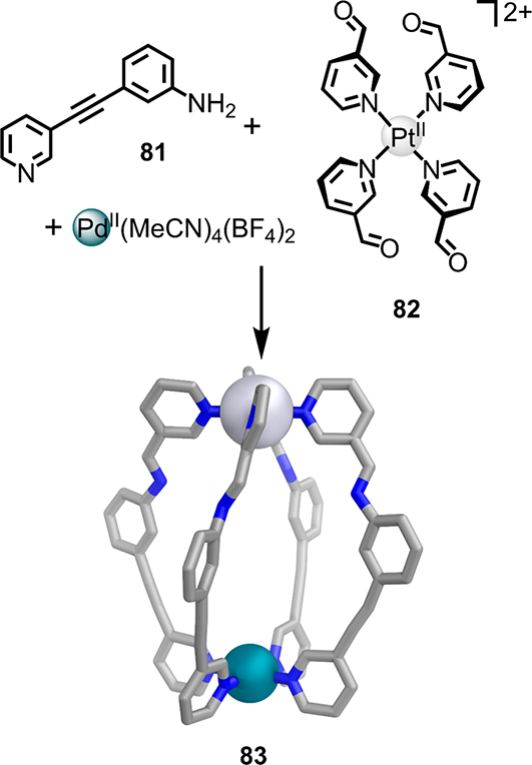
Synthesis of [Pd^II^Pt^II^L_4_](BF_4_)_4_ cage **83** via the combination of preformed Pt^II^(3-pyridylcarboxaldehyde)_4_ complex **82**, 3-[2-(3-pyridinyl)ethynyl]aniline (**81**), and Pd^II^(MeCN)_4_(BF_4_)_2_.^138^

An approach introduced by Crowley et al. is based on the design and synthesis of a cage in which the antipodes are Pt^II^, which forms more inert Pt^II^–pyridyl bonds, and Pd^II^, which forms more labile Pd^II^–pyridyl bonds.^138^ Following its formation (Figure 20), Pd^II^Pt^II^L_4_ cage **83** can open and close reversibly. The addition of 4-dimethylaminopyridine (DMAP) selectively sequesters Pd^II^, forming Pd^II^(DMAP)_4_ and opening the cage. Subsequent addition of *p*-toluenesulfonic acid protonates the DMAP ligands and causes their dissociation from the metal centers, releasing Pd^II^ and reforming cage **83**.

This stimulus-induced opening and closing of cage **83** also brings about reversible guest uptake and release, illustrating a potential function. Although these structures are relatively simple, they exemplify how functionality can be introduced by the use of reduced-symmetry ligands. These principles may be combined with other rules, detailed elsewhere in this review, that guide the formation of larger and more complex structures to yield architectures of greater complexity and functionality.

**Figure 21:**
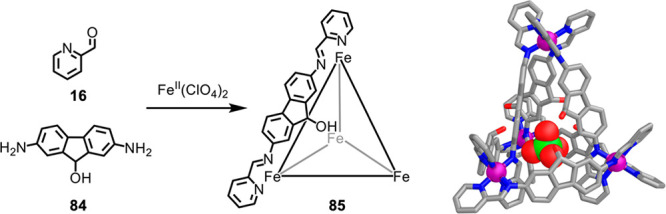
Hooley’s “wizard’s hat” assembly **85**, stabilized by internal hydrogen bonds and a templating perchlorate ion. The crystal structure shown at the right.^140^

Hooley and co-workers reported the use of a prochiral ligand in the assembly of a desymmetrized Fe^II^_4_L_6_ architecture (Figure 21).^140^ The presence of a prochiral CHOH center in the fluorenone ligand—a motif that they have explored to generate functional capsules^141−144^—brought about the selective formation of “wizard’s hat” **85**, a distorted tetrahedron. The formation of this unusual architecture is favored by a specific pattern of hydrogen bonding involving the −OH groups at the prochiral carbon atoms of the ligands and a templating perchlorate ion at the base of the assembly. An interesting aspect of this assembly is the presence of three *mer* Fe^II^ centers at the base of the structure, which are rare in self-assembled pyridylimine architectures and often drive the assembly of more complex structures, as discussed in subsequent sections.

Along with reduced-symmetry ditopic ligands, tritopic ligands with reduced symmetry can generate complex metal–organic architectures. Su et al. demonstrated the use of such tritopic ligands to form unusual architectures in the preparation of a Ag^I^_6_L_6_ tubular structure using an elongated T-shaped ligand.^145^

**Figure 22:**
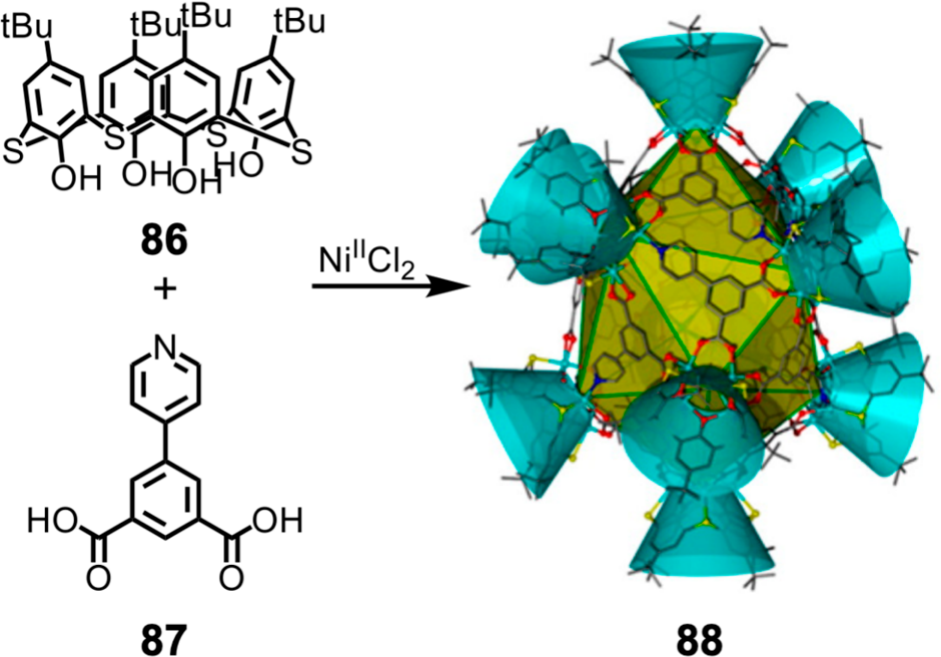
Ni_40_ coordination cage **88** with a structure corresponding to the *J*_17_ Johnson solid, formed from *p*-*tert*-butylthiacalix[4]arene (**86**), 5-(pyridin-4-yl)isophthalic acid (**87**), and Ni^II^Cl_2_. Reproduced from ref (146). Copyright 2016 American Chemical Society.

Hu et al. used 5-(pyridin-4-yl)isophthalic acid (**87**) with *p*-*tert*-butylthiacalix[4]arene (**86**) and Ni^II^Cl_2_ to form Ni^II^_40_ coordination cage **88**, with a structure corresponding to the J_17_ Johnson solid.^146^ As illustrated in Figure 22, the structure of **88**, a gyroelongated square bipyramid, consists of 10 Ni_4_-*p*-*tert*-butylthiacalix[4]arene shuttlecock-like vertices and 16 panels of ligand **87**. Four ligands converge at two of the 10 vertices, and five ligands converge at each of the other eight, closing the faces of the structure. In order to form the structure, the ligands coordinate to Ni^II^ centers through different donor atoms: through the carboxylate, which can either bridge or chelate Ni^II^, and through the nitrogen donor of pyridine. The phenoxo oxygen and sulfur atoms of the *p*-*tert*-butylthiacalix[4]arene units also coordinate to Ni^II^, along with additional **87** units that do not cap the faces of the structure, DMF molecules, chloride ions, and degradation products of DMF in order to satisfy the coordination geometry of Ni^II^.

**Figure 23:**
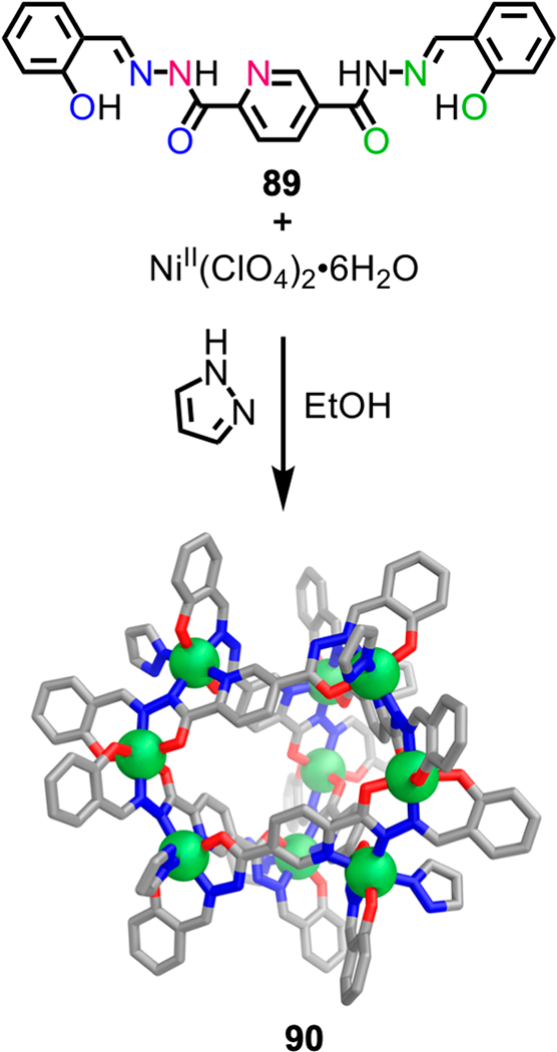
Self-assembly of Ni^II^_9_**89**_6_Pz_6_ barrel structure **90** incorporating asymmetric tritopic ligand **89**.^147^

Hong et al. employed tritopic ligand **89**, which has three binding sites arrayed asymmetrically along its length (Figure 23). The combination of this reduced-symmetry ligand, Ni^II^(ClO_4_)_2_, and pyrazole (Pz) in ethanol yields Ni^II^_9_**89**_6_Pz_6_ barrel structure **90**.^147^ In **90**, the pyrazole plays two roles, acting as a Lewis base and as an additional donor to satisfy the octahedral coordination sphere of Ni^II^.

Li et al. explored the use of desymmetrized tetratopic ligands resembling trapezoids to form metallosupramolecular architectures. Upon combination of these ligands with 180° dipalladium(II) acceptors, ring-in-ring^148^ or 2D Star-of-David^149^ structures form.

**Figure 24:**
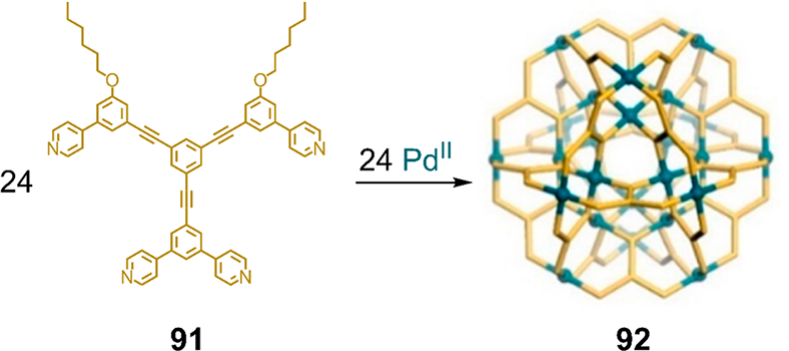
Self-assembly of Pd^II^_24_**91**_24_ three-dimensional sphere-in-sphere structure **92**. Adapted from ref (148). Copyright 2015 American Chemical Society.

The reaction of these same ligands with “naked” palladium(II) ions yields three-dimensional structures. One example is Pd^II^_24_**91**_24_ sphere-in-sphere architecture **92** (Figure 24), which forms from ligand **91** and Pd^II^.^148^ The authors drew a contrast between their approach and the one pioneered by Fujita and co-workers.^150^ The Fujita approach is based on the orthogonal assembly of two ditopic units into “independent” M_12_L_24_ spheres connected via flexible linkers to give the M_24_L_24_ sphere-in-sphere. In Li’s system (Figure 24) precise preorganization of the entire 3D architecture is enforced by the rigid nature of the ligand. Ligand **91** also reacts with a tritopic platinum(II) unit to form a double-layered pentagonal prism.^151^

Ligand **91** has donor groups arrayed in two distinct ways; Li et al. also designed ligands with four distinct binding sites that form double-layered macrocyclic structures.^152^ Their reports exemplify how rational design of new classes of ligands can allow unique metallosupramolecules with high degrees of complexity to be formed.

### 3.2 Additional Donor Sites

**Figure 25:**
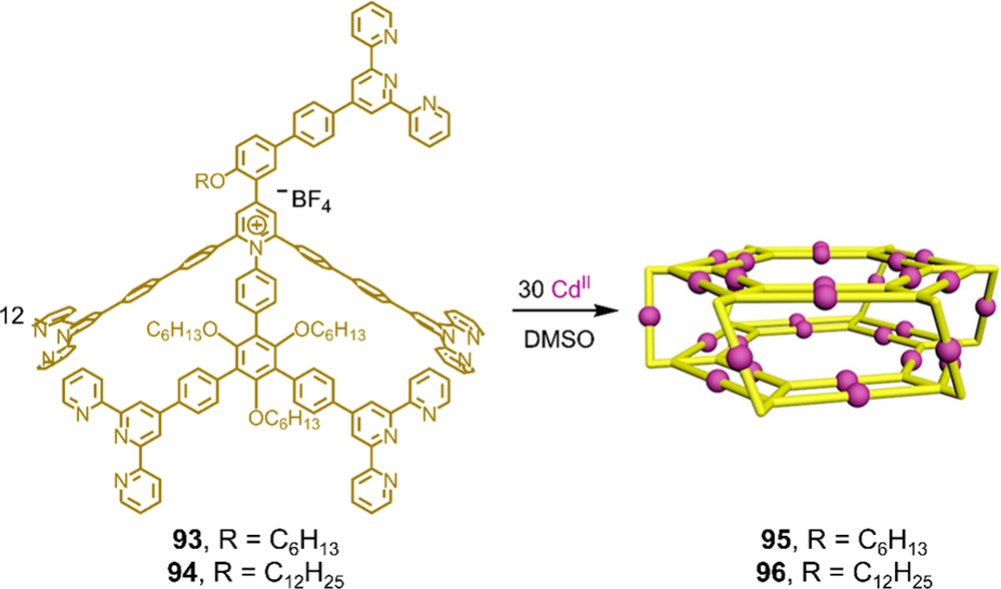
Self-assembly of three-dimensional hexagonal-prismatic structures **95** and **96**, consisting of two connected 2D double-rimmed “Kandinsky circles”, from Cd^II^ and ligands **93** and **94**, respectively. Adapted from ref (153). Copyright 2019 American Chemical Society.

Another approach to designing ligands capable of forming architectures with greater complexity is the modification of ligands that have previously been used to form metal–organic assemblies, for example by appending additional donor sites. This approach was used to design pentatopic ligands **93** and **94** (Figure 25), which form 3D hexagonal-prismatic structures **95** and **96**, consisting of two connected 2D double-rimmed “Kandinsky circles”, when combined with octahedrally coordinated cadmium(II) ions.^153^ Ligands **93** and **94** are based upon a tetratopic donor previously reported by Li et al.,^154^ with the fifth terpyridine group appended to allow the two circles to be linked.

**Figure 26:**
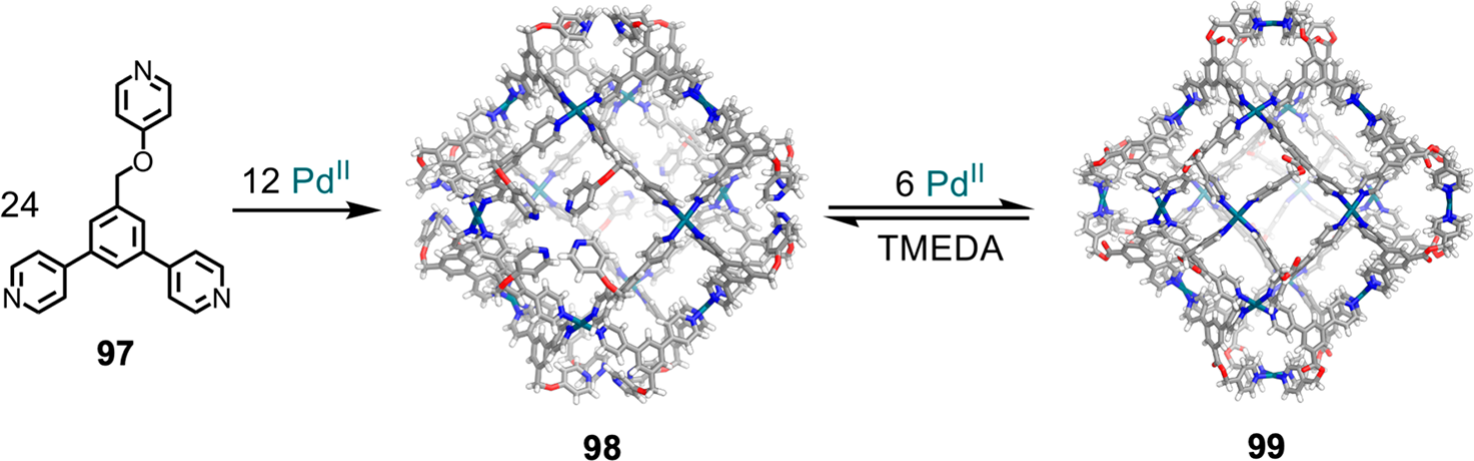
Stepwise assembly of Pd^II^_18_**97**_24_ stellated cuboctahedron **99** from terpyridine **97** and Pd^II^.^155^

As well as providing a method for the formation of 3D structures from known 2D structures, the ligand-modification approach can be used to increase the complexity of an existing 3D structure. Fujita and co-workers employed this approach to form a Pd^II^_18_**97**_24_ stellated cuboctahedron **99** using tripyridyl ligand **97**, consisting of a rigid bipyridyl unit with a third pyridyl moiety flexibly tethered to the backbone.^155^ As shown in Figure 26, the assembly process occurs in a stepwise fashion. The tripyridyl ligand combines with Pd^II^(BF_4_)_2_ to yield Pd^II^_12_**97**_24_ cuboctahedron **98**, analogous to a previously reported complex that incorporates a rigid bipyridine ligand.^156^

Initial selective complexation with just one type of pyridine donor to form **98** is perhaps surprising at first sight. The authors suggested that the selectivity observed is due to the high kinetic stability of the cuboctahedral framework. Previous work had shown that ligand exchange on a completed cuboctahedron occurs with a half-life of 20 days.^157^ Kinetic trapping of the cuboctahedron thus drives the selective assembly.

Subsequent addition of more Pd^II^(BF_4_)_2_ to intermediate structure **98** resulted in capping of the square faces by the coordination of four “free” pyridyl groups to each new palladium(II) center and consequent stellation of the structure to form **99**. Stellation is reversed by the addition of *N*,*N*,*N*′,*N*′-TMEDA, resulting in the reformation of **98**. The authors noted that this reversible opening and closing through stellation may have future applications in guest capture and release.

### 3.3 Nonplanar Macrocyclic Ligands

As shown in the system in Figure 22, macrocycle-derived subunits can be employed to construct coordination cages.^146,158−161^ These components often have greater complexity than simpler small-molecule ligands, while still maintaining high symmetry, which increases the complexity of the resulting metal–organic architectures.^162,163^ Furthermore, the use of macrocycle-derived components also may enable combination of the guest-binding abilities of the macrocycles with those of the higher-order superstructures that the macrocycles form.^164−167^

Complementing the work of Hu and co-workers, who used *p*-*tert*-butylthiacalix[4]arene to form the vertices of a metal–organic polyhedron,^146^ macrocyclic components have also been employed as the edges and faces of metal–organic cages. Hardie and co-workers reported foundational work in this area using tritopic cyclotriveratrylene (CTV)-related ligands.^168−170^

**Figure 27:**
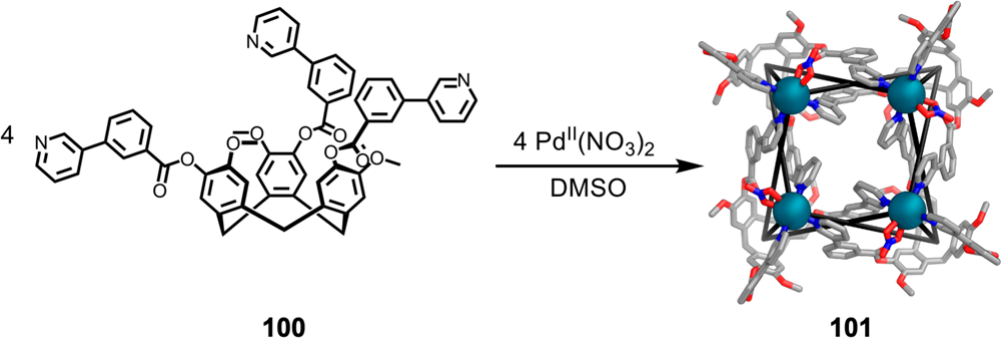
Assembly of Pd^II^_4_**100**_4_ “Solomon’s cube” **101**.^170^

The Hardie group’s use of CTV-related ligands to provide an array of new structure types culminated in the report of a “Solomon’s cube”,^170^ based upon the topology of a Solomon link.^171,172^ The combination of extended tris(pyridyl)cyclotriguaiacylene (**100**) with Pd^II^(NO_3_)_2_ in DMSO results in Pd^II^_4_**100**_4_ structure **101** shown in Figure 27. While resembling a Solomon link,^171,172^ with alternating under and over crossing points of two rings, **101** has additional connections between the rings, linking them. Consequently, the structure was described as a “Solomon’s cube”, with square faces and eight triply connected vertices.

Structure **101** may thus be considered in terms of its three stereochemically distinct subunits: ligand **100**, the Solomon link, and the figure-eight motifs lying on each of four sides of the structure. The crystal structure shows two enantiomers, in which all three of these elements concertedly show opposite handedness.

The driving force for the formation of smaller Pd^II^_4_**100**_4_ assembly **101**, as opposed to a Pd^II^_6_**100**_8_ structure, is likely due to the fact that ligand **100** contains *m*-pyridine-based arms, as opposed to a linear *para* ligand regiochemistry, connected to a rigid macrocyclic core. Further stabilization of this topology may come from interligand π-stacking interactions.

Structure **101** in Figure 27 thus demonstrates the ability of nonplanar macrocycle-based ligands to produce more complex structure types than would be observed in analogous cases using planar *D*_3*h*_-symmetric ligands. Interwoven **101** also exemplifies how the use of novel classes of ligands can lead to serendipitous discoveries.

### 3.4 Metallosupramolecular Chemistry Meets DNA Nanotechnology

**Figure 28:**
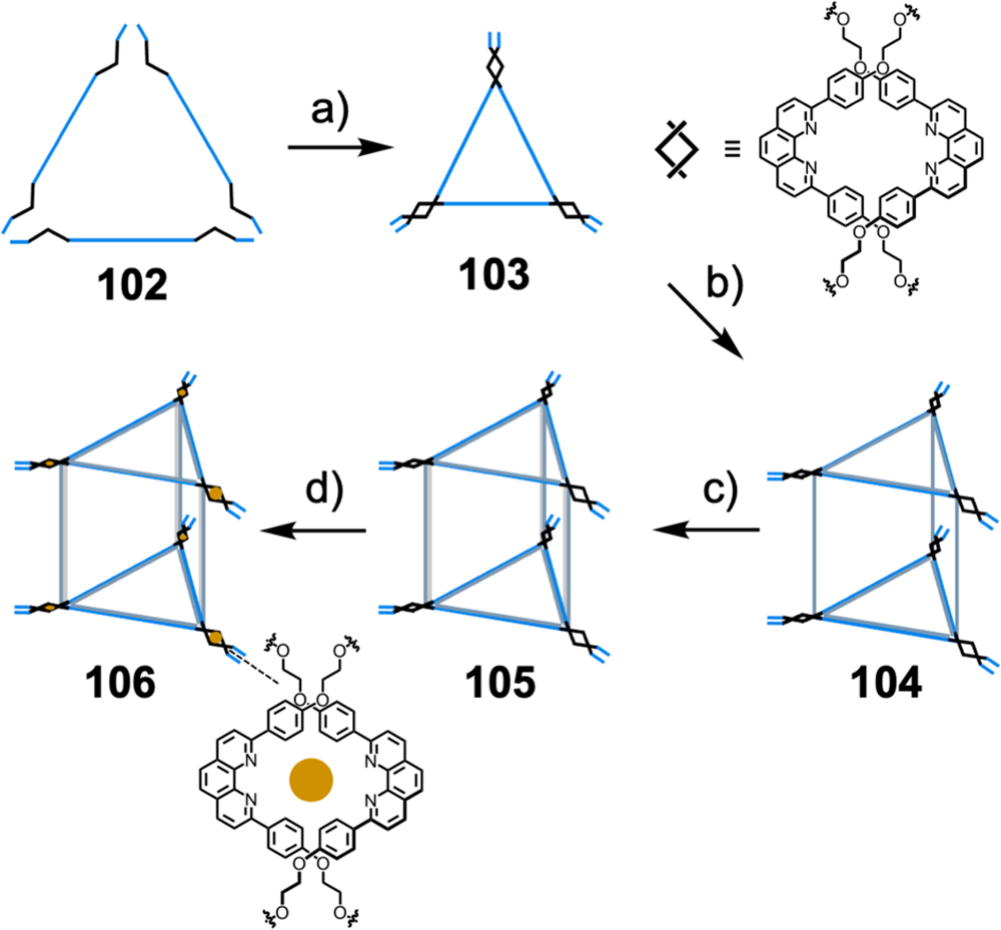
Stepwise assembly of metal–DNA cages from diphenylphenanthroline-containing DNA strands. (a) Hybridization of three complementary oligonucleotide strands. (b) Linking of two triangles with single strands. (c) Rigidification of the linking strands. (d) Site-specific metalation.^173^

Many of the architectures discussed in this review are assembled using small-molecule organic ligands and metal ions. A more exotic example is provided by the metal–nucleic acid cages of Sleiman et al.^173^ (Figure 28). These structures require stepwise assembly of oligonucleotide strands (**102**). First, triangles **103** with corners consisting of two 2,9-diphenyl-1,10-phenanthroline ligands (dpp–dpp) are formed through hybridization of three complementary oligonucleotide strands. Second, two triangles are linked with single strands to give **104**, and the struts are then rigidified to form trigonal-prismatic structures **105**. Finally, site-specific metalation involving the coordination of Cu^I^, Ag^I^, Au^I^, Zn^II^, Co^II^, Cd^II^, or Eu^II^ to the dpp–dpp sites, enables the creation of metal–DNA cages **106**.^173^

The Sleiman group also demonstrated that the order of the steps could be swapped: premetalation of the triangles followed by single-strand triangle linkage and rigidification results in the same metalated trigonal-prismatic structures. Although the flexibility in the order of construction steps indicates that metal–ligand coordination is not required to template the formation of these trigonal-prismatic structures, metalation of the structures increased their resistance to both chemical and thermal denaturation compared with their demetalated counterparts. Metal coordination was thus demonstrated to enable the formation of robust architectures assembled from strands of DNA, potentially enhancing the range of applications of 3D DNA architectures.^174−178^

Through highlighting some key examples of complex or reduced-symmetry ligands that have led to novel structures, this section has emphasized the roles of both rational design and serendipity. As a general approach, the use of reduced-symmetry and complex ligands often involves rational design, sometimes with the aid of computational predictions. Postassembly rationalization has in many cases also played a role, enabling the discovery of new assembly rules, which may then be used for future designs.

## 4 Ligand Flexibility Drives Structural Complexity

Flexible ligands in many cases assemble into high-symmetry architectures.^179−185^ However, flexibility within a ligand can also extend the scope of structure types beyond those having high symmetries. This section summarizes novel structure types generated via the incorporation of flexibility into the building blocks used to assemble discrete structures. Ligand flexibility often generates serendipitous results, as ligand degrees of freedom are deployed in unforeseen ways.

### 4.1 Flexible Ditopic Ligands

Ward and co-workers pioneered the construction of metal–organic architectures with flexible ditopic ligands, focusing on ligands containing two bidentate pyrazolylpyridine chelating sites, each attached to a central aromatic group via flexible methylene linkages. These ligands were combined in a 3:2 ratio with octahedral metal centers to yield several distinct structure types. Some of these structures have the geometries of Platonic solids, such as tetrahedra,^179−181^ and others have lower symmetries and greater complexity.

**Figure 29:**
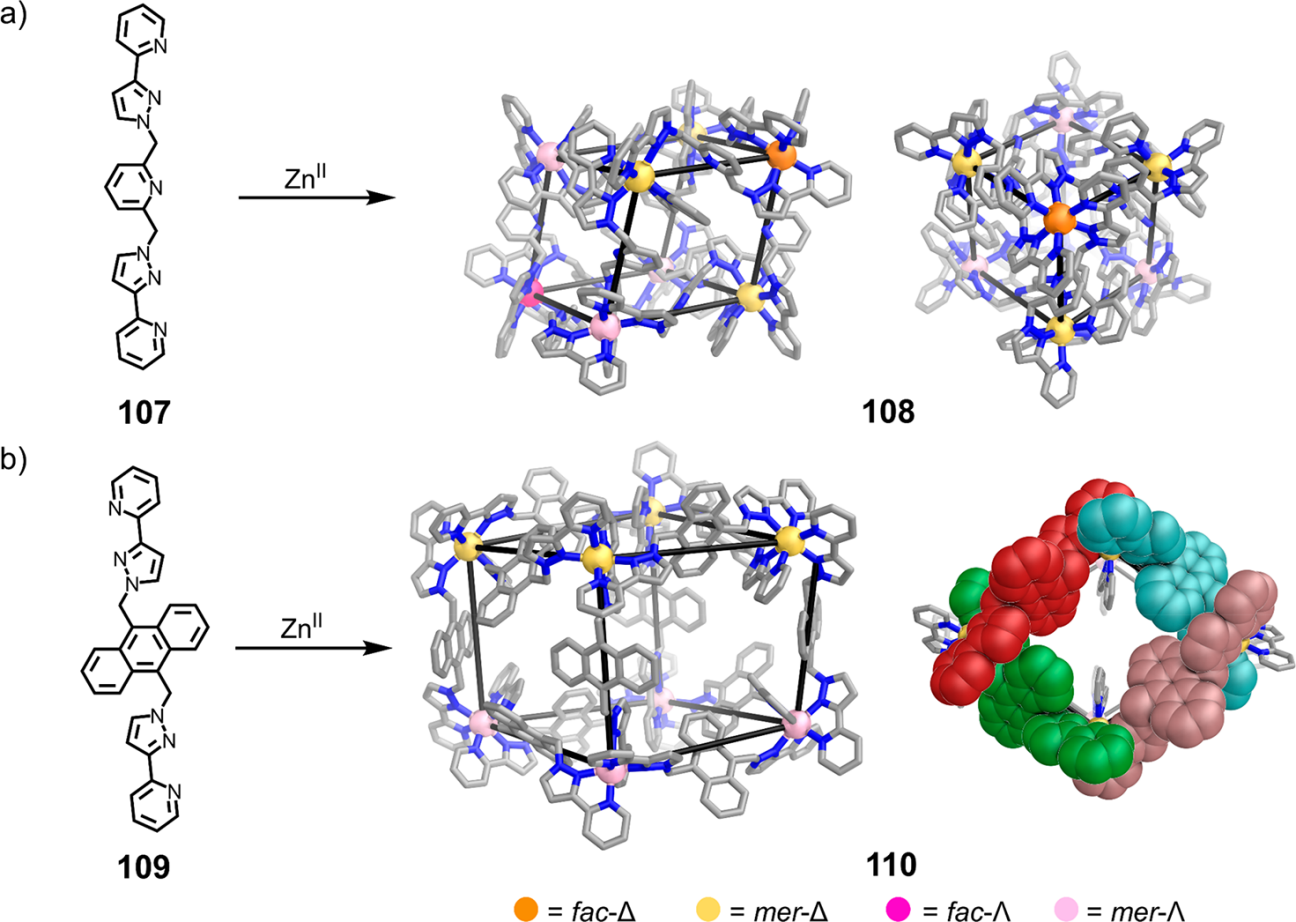
Two distinct M^II^_8_L_12_ structures formed using ditopic bis(pyrazolylpyridine) ligands and octahedral metal centers.^186,187^

Several of Ward’s M_8_L_12_ structures exhibit symmetry reduced from that of a cube.^186,187^ For example, as shown in Figure 29a, the combination of **107** with Zn^II^ yields Zn^II^_8_**107**_12_ cuboid **108** with *S*_6_ symmetry. An antipodal pair of Zn^II^ centers define the *S*_6_ axis of the structure. These metal centers have *fac* stereochemistry but opposite handedness.^186^ The other six metal centers have *mer* stereochemistry and are grouped into two sets of three. All of the metal centers within the same set have the same handedness, opposite to that of the other set. Mass spectrometry data showed the formation of an analogous M^II^_8_L_12_ structure, Co^II^_8_**107**_12_, from Co^II^ and **107**.

With anthracene-cored ligand **109**, structure **110** was formed, which has the same M^II^_8_L_12_ composition as **108** but significant structural differences. Cuboid **110** consists of two connected Zn^II^_4_**109**_4_ cyclic helical units (Figure 29b).^187^ Within each tetrameric unit, the four metal centers are trischelated in a *mer* fashion and have the same absolute configuration. However, as shown in Figure 29b, the handedness of the four metal centers in one tetrameric unit is opposite to that of the metal centers making up the other tetrameric face. The use of Cu^II^(BF_4_)_2_ with **109** yields Cu^II^_8_**109**_12_, which has a similar structure as **110**.

The diversity of structures formed using such ligands was further demonstrated by the formation of unusual Ni^II^_4_L_6_ “square” and M^II^_6_L_9_ (M^II^ = Zn^II^, Co^II^) “open book” structures using **107** and its modified derivatives.^188,189^

**Figure 30:**
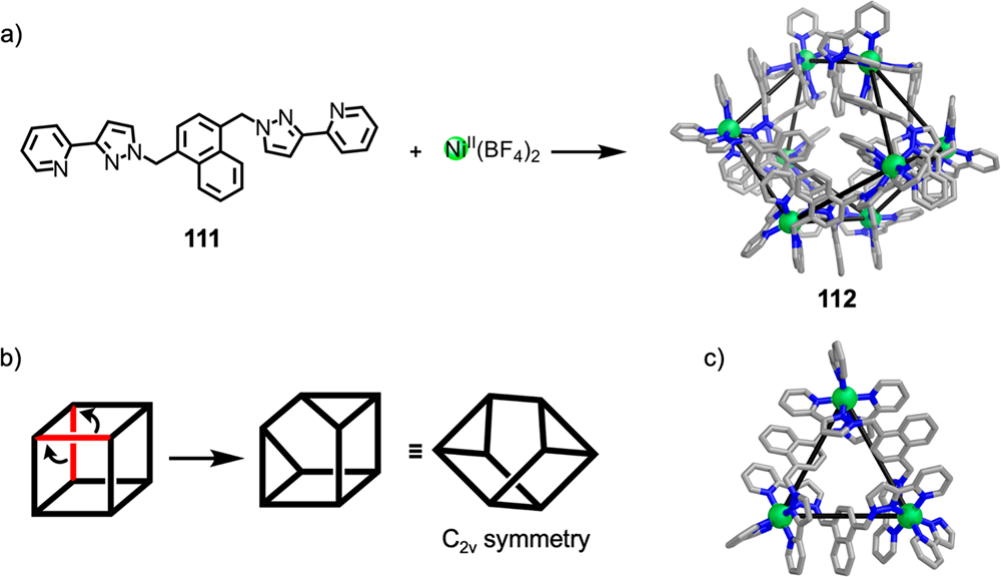
(a) Formation of Ni^II^_8_**111**_12_ complex **112** with a structure based on a “cuneane-like” core.^190^ (b) The “cuneane” structure is obtained by the rearrangement of two edges of a cube. (c) View perpendicular to one of the Ni^II^_3_L_3_ cyclic helical units making up the two triangular faces of **112**.^190^

Ligand **111** reacts with Ni^II^(BF_4_)_2_ in a 3:2 ratio in MeOH/CH_2_Cl_2_ (Figure 30) to yield a Ni^II^_8_**111**_12_ structure, which was initially thought to be cubic.^190^ However, X-ray crystallography showed that product **112** has an unusual structure, based on *C*_2*v*_-symmetric cuneane formed by the rearrangement of two edges of a cube (Figure 30b). All eight of its metal centers have *mer* stereochemistry. Interestingly, seven of the metal centers have the same absolute configuration, with the eighth displaying the opposite handedness.

**Figure 31:**
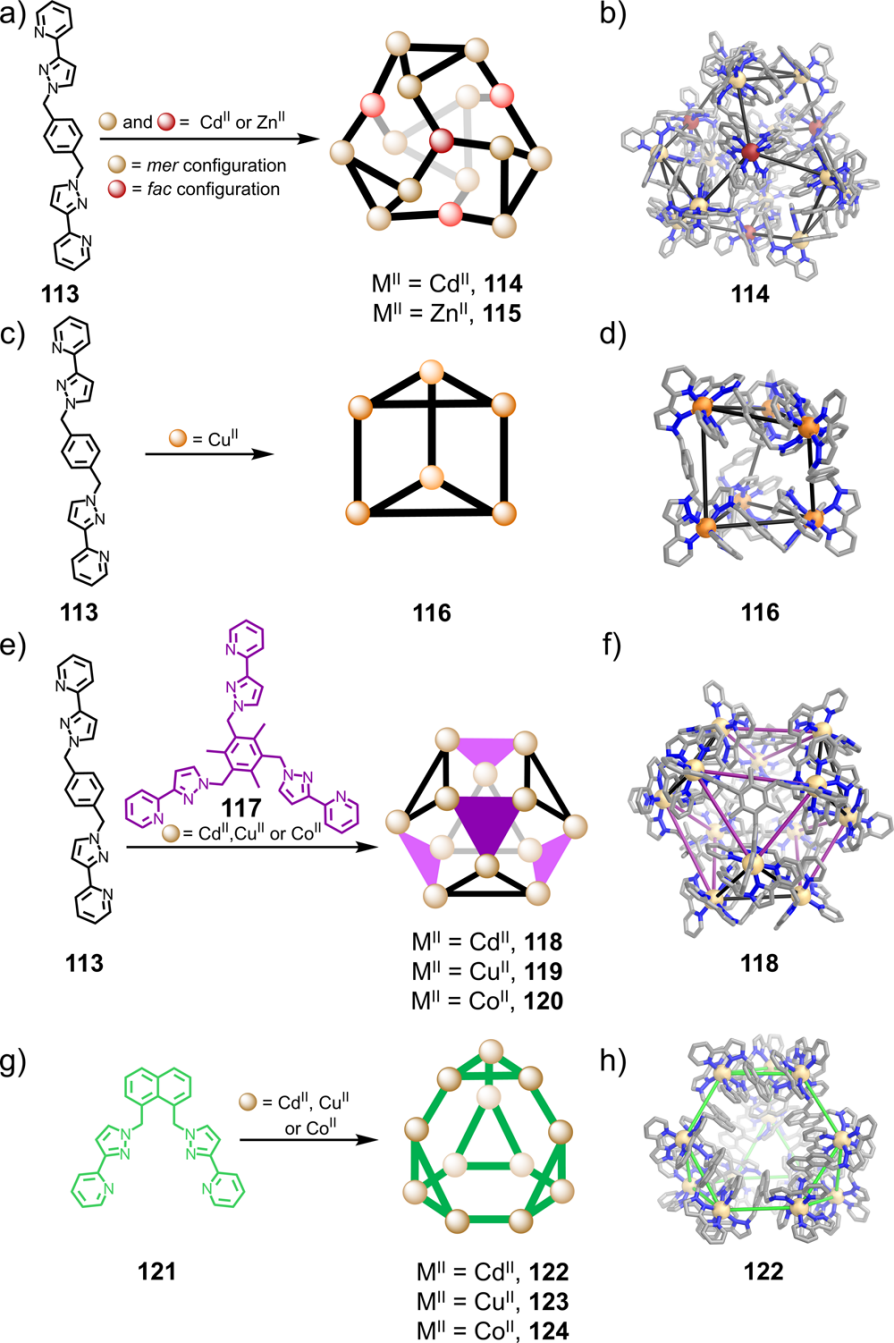
Four different structure types containing M_3_L_3_ circular helicate units. (a) Schematic view of M^II^_16_**113**_24_, (b) Cd^II^_16_**113**_24_,^191^ (beige and red spheres correspond to *mer* and *fac* configurations, respectively), (c) schematic view of M^II^_6_**113**_9_, (d) Cu^II^_6_**113**_9_,^191^ (e) schematic view of M^II^_12_**113**_12_**117**_4_, (f) Cd^II^_12_**113**_12_**117**_4_,^192^ (g) schematic view of M^II^_12_**121**_18_, and (h) Cd^II^_12_**121**_18._^193^

Each of the two triangular faces of **112** is made up of an M^II^_3_**111**_3_ metallomacrocycle (Figure 30c). Such M_3_L_3_ units have been observed in structures employing similar ditopic ligands.^191−193^ The four structure types shown in Figure 31 are built from M_3_L_3_ subunits, with their different geometries arising from differences in how these subunits are connected to each other.

As shown in Figure 31a, structure **114**, a Cd^II^_16_**113**_24_ twisted tetracapped truncated tetrahedron, results from the reaction of **113** and Cd^II^ in MeCN; Zn^II^ also forms the analogous structure **115**.^191^ Within **114**, four Cd^II^_3_**113**_3_ cyclic helical subunits are linked by Cd^II^**113**_3_ units, which act as tritopic complex ligands (Figure 31b). The *fac*-configured Cd^II^ centers of the Cd^II^**113**_3_ units (red spheres in Figure 31a,b) cap each of the four hexagonal faces of a Cd^II^_12_ distorted truncated tetrahedral core described by the 12 *mer*-configured centers (beige spheres in Figure 31a,b) of the four Cd^II^_3_**113**_3_ units. When ligand **113** reacts with Cu^II^, the smaller Cu^II^_6_**113**_9_ trigonal-prismatic structure **116** forms (Figure 31c). Trigonal prism **116** consists of two Cu^II^_3_**113**_3_ circular helical units bridged by three ligands, with some offset between triangular faces leading to distortion toward a trigonal-antiprismatic structure (Figure 31d).

The reaction of Ni^II^(BF_4_)_2_ with **113** produces a Ni^II^_8_**113**_12_ cubic cage, which does not contain trinuclear helicate units. The observation of different structures with the same ligand but different metal ions was attributed to variations in the ionic radii and stereoelectronic preferences of the metal centers.^191^ Furthermore, reaction of the same ligand (**113**) together with flexible tris-bidentate ligand **117** and Cd^II^, Cu^II^, or Co^II^ in a 3:1:3 ratio yields a [M^II^_12_**117**_4_**113**_12_] cage with approximately cuboctahedral geometry (Figure 31e).^192^ Of its eight triangular faces, four are capped by **117**, and each of the remaining four consists of an M^II^_3_**113**_3_ circular helical subunit, similar to those found in the other structures.

The fourth structure type, shown in Figure 31g, is an M^II^_12_**121**_18_ truncated tetrahedral cage framework with idealized *T* symmetry. This structure results from the reaction of **121**, which has a naphthyl central linking group, with Cu^II^, Co^II^, or Cd^II^.^193,194^ These structures consist of four M_3_**121**_3_ circular helical motifs that are connected directly by six bridging ligands.

A common thread linking the different geometries shown in Figure 31 is the presence of linked M^II^_3_L_3_ circular helicate subunits, where the three metal centers have a *mer* trischelate geometry. Another important feature of these four structure types is the prevalence of interligand aromatic stacking interactions, often between electron-rich central aromatic moieties on one ligand and electron-deficient pyrazolylpyridine units on another.^191−193^ This elegant work by the Ward group has thus established the utility of relatively simple, flexible ligands in the construction of assemblies with structures beyond the Platonic solids, whose geometries are controlled by subtle variations in reaction conditions and ligand structure.

^1^H NMR spectroscopy and mass spectrometry showed that the Cd^II^_16_**113**_24_ structure **114** described above is initially present in solution, but the structure rearranges to give a smaller Cd^II^_6_**113**_9_ trigonal prism over weeks in solution.^191^ Replacing the 1,4-phenyl moiety in **113** with the 1,4-naphthyl in ligand **111** results in a Cd^II^_16_**111**_24_ tetracapped truncated tetrahedron (in contrast to the cuneane structure observed for **111** with Ni^II^, shown in Figure 30a), which does not rearrange in solution. The additional interligand π stacking provided by the naphthyl spacer was inferred to stabilize the tetracapped truncated tetrahedron in solution.^195^

In contrast, the reaction of **111** with Cu^II^ does not selectively yield any species analogous to those shown in Figures 30 and 31. Instead, crystals of an unusual Cu^II^_12_**111**_15_ structure form in low yield, consisting of two Cu^II^_3_**111**_3_ units linked by an equatorial belt of six Cu^II^ ions, each with a coordination number of 4 or 5.^195^

Utilizing ligand **111** also allowed Ward et al. to analyze the Cd^II^**111**_12_**117**_4_ analogue of the structures shown in Figure 31e,f in solution. Cd^II^**111**_12_**117**_4_ was shown to exist as three different diastereomers in solution, with *T*, *C*_3_, or *S*_4_ symmetry.^196^ The difference between the diastereomers arises from the different relative helical handednesses of the four Cd^II^_3_L_3_ circular helical units in the structure.

**Figure 32:**
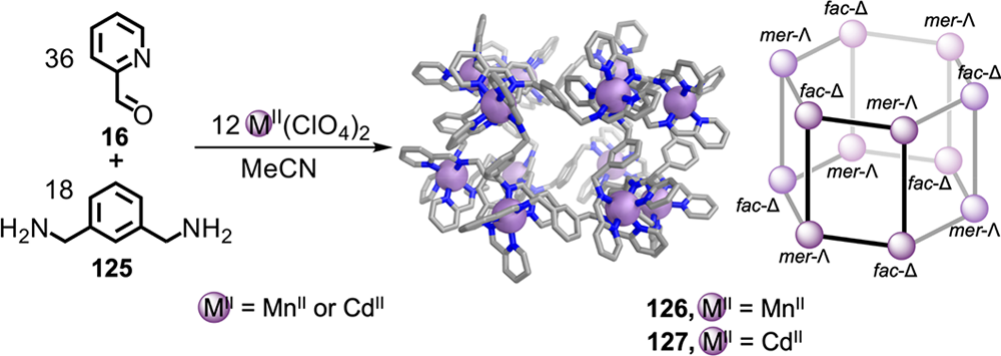
Subcomponent self-assembly of *D*_3_-symmetric M^II^_12_L_18_ hexagonal-prismatic structures **126** and **127**.^197^

Kwong et al. reported the formation of *D*_3_-symmetric M^II^_12_L_18_ hexagonal-prismatic architectures following the reaction of 2-formylpyridine **16**, *m*-xylylenediamine **125**, and Mn^II^(ClO_4_)_2_ or Cd^II^(ClO_4_)_2_ in acetonitrile (Figure 32).^197^ The crystal structure of **126** reveals two M_6_L_6_ hexagons having chair conformations, made up of alternating Λ- and Δ-configured metal centers. Bridging ligands connect metal centers with a Λ configuration on one ring with those with a Δ configuration on the other, resulting in *mer*-Λ and *fac*-Δ configured metal centers within prism **126**. Other metal–organic structures beyond the Platonic solids constructed using similarly flexible ditopic ligands include a Hg^II^_4_Cl_8_L_4_*S*_4_-symmetric coordination nanotube^198^ and a [Dy^III^_8_L_8_(μ_2_-CH_3_OH)_4_]^8+^ dual triple-stranded helicate.^199^

**Figure 33:**
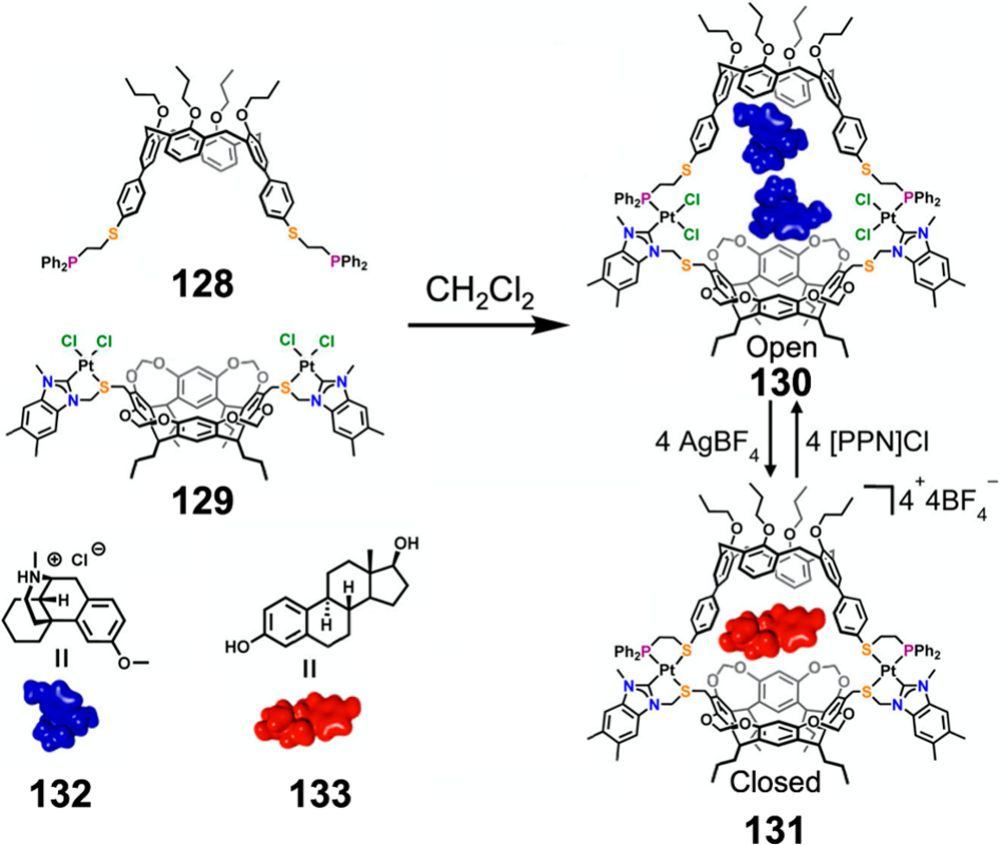
Controlled guest release and uptake by means of the “weak-link” approach using ligands **128** and **129**. [PPN]Cl = bis(triphenylphosphine)iminium chloride. Adapted from ref (201). Copyright 2017 American Chemical Society.

Mirkin and co-workers developed the “weak-link approach” to forming reduced-symmetry structures with complex functions.^200^Figure 33 shows a dimeric capsule produced using this approach, incorporating resorcin[4]arene and calix[4]arene subunits linked by platinum(II) centers.^201^ In the absence of chloride, “weak-link” thioethers coordinate to platinum(II) binding sites. Upon the addition of chloride ions, these thioethers are selectively displaced, causing expansion of the cavity. The addition of silver(I) tetrafluoroborate reverses this expansion by abstracting chloride from the platinum(II) centers and regenerating the closed state of the capsule. In the thioether-coordinated form **131**, estradiol **133** is bound selectively. In the chloride-coordinated form **130**, two molecules of dextromethorphan·HCl (**132**) bind instead. Sequential addition of chloride to **131** and silver(I) tetrafluoroborate to **130** brings about reversible binding and release of dextromethorphan, showcasing the ability to reversibly generate cavities with different sizes and shapes and thus control guest binding.

### 4.2 Flexible Tritopic Ligands

**Figure 34:**
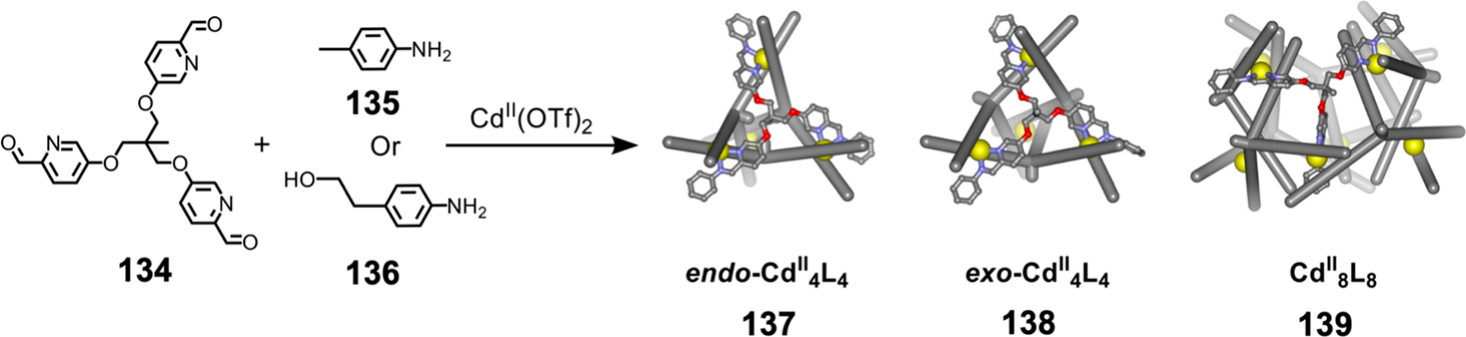
Conditions-dependent subcomponent self-assembly of three discrete products: tetrahedra **137** and **138**, and Cd^II^_8_L_8_ tetragonal antiprism **139** with *D*_4_ point symmetry. Adapted from ref (202). Copyright 2016 American Chemical Society.

The combination of flexible *tris*-formylpyridine subcomponent **134** with Cd^II^(OTf)_2_ and *p*-toluidine (**135**) yields a mixture of three products (Figure 34).^202^ Two of these are *T*-symmetric Cd^II^_4_L_4_ tetrahedra (**137** and **138**). In **137**, the central methyl groups of the ligands point inside the cavity (*endo*), whereas in **138** these methyl groups point outward (*exo*). The third, minor, product is Cd^II^_8_L_8_ tetragonal antiprism **139** with *D*_4_ point symmetry. The eight metal centers defining the vertices of the structure have the same handedness, each with a *mer* arrangement of ligands.

The relative amount of the Cd^II^_8_L_8_ antiprismatic structure **139** grows with increasing concentration because of a reduction in the entropic penalty of forming a larger Cd^II^_8_L_8_ species instead of the smaller Cd^II^_4_L_4_ complexes. Even more effective at driving the formation of the Cd^II^_8_L_8_ structure is the use of 2-(4-aminophenyl)ethanol (**136**) as a subcomponent in place of **135** and the use of a 1:3 CH_2_Cl_2_/MeCN solvent mixture. We hypothesized that these conditions allow the formation of stabilizing hydrogen-bonding interactions between the hydroxy groups of the aniline residues in the Cd^II^_8_L_8_ antiprismatic structure. In this example, the analysis of a serendipitous result enabled the rational development of design principles for the optimized preparation of a complex architecture, illustrating the synergy between serendipity and rational design.

Hong et al. used a tris(pyridine) ligand, which had a flexible core similar to that of **134**, for the construction of open Ag^I^_6_L_4_ cages upon reaction with Ag^I^BF_4_.^203^ These cages undergo further assembly to produce higher-order polycatenanes and polycages, depending on the reaction conditions.

### 4.3 Flexible Tetratopic Ligands

**Figure 35:**
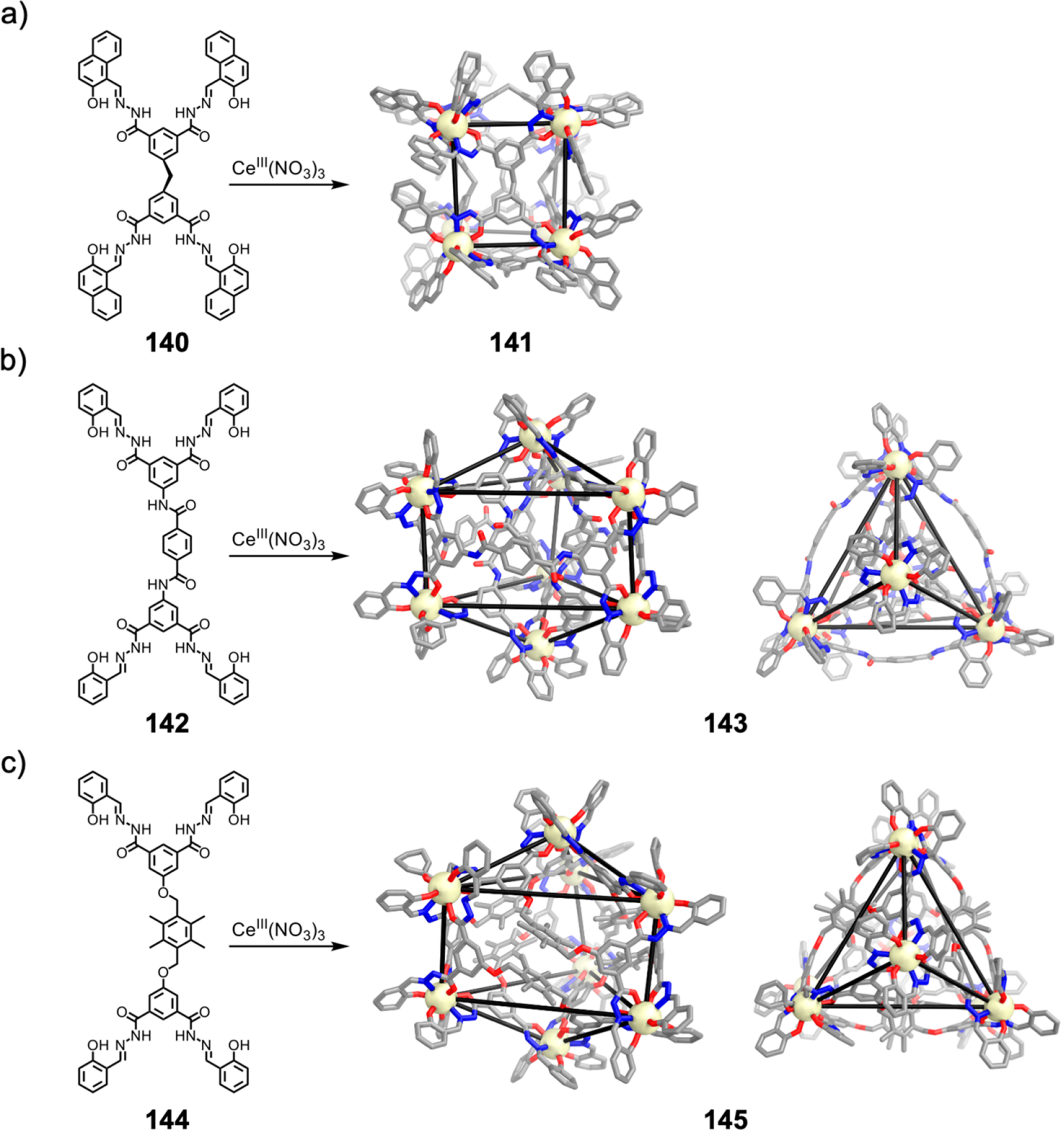
With Ce^III^, tetratopic ligands (a) **140**, (b) **142**, and (c) **144** form cuboid **141** and bicoronal trigonal prisms **143** and **145**, respectively.^205−207^

In sections 7.2 and 7.3 we explore how barrel-like and other complex architectures have been constructed using tetratopic ligands that are elongated along one axis or curved. Expanding upon this approach, Duan et al. used tetratopic ligands with flexible linkers separating two bis-tridentate units to prepare structure types that include trigonal-prismatic barrels, cubelike structures, and bicoronal trigonal prisms.^204−207^ Assembly **141** (Figure 35a) is a Ce_8_**140**_6_ cuboidal architecture with pseudo-*S*_4_ symmetry formed from Ce^III^(NO_3_)_3_, KOH, and ligand **140**.^205^ The crystal structure of **141** shows that four of its ligands have their long axes aligned, with their central methylene groups bent toward the inside of the cage, and the ligands at the top and bottom of the structure both have their methylene groups bent toward the outside of the cage.

However, such a pseudocubic structure type does not form when the similar ligands **142** and **144** are used (Figure 35b,c). Instead the Ce_8_L_6_ complexes **143** and **145**, respectively, are formed. Assembly **143** consists of a Ce_6_**142**_3_ trigonal-prismatic framework, with two additional metal centers and three ligands forming a helical pillar within the prism. Two of the tridentate moieties of each ligand in the helical pillar bind to the apical cerium centers, and the other two tridentate sites chelate two of the metal centers making up the prismatic framework (Figure 35b).^206^ In contrast, **145** has a cagelike structure in which the flexible ligand twists so that the four cerium centers binding to the same ligand are not coplanar.^207^ Furthermore, stacking interactions between the benzyl groups on neighboring ligands are inferred to stabilize the unusual structure of **145**.^207^

Sun and co-workers also reported the use of flexible tetratopic ligands in the synthesis of unusual “conjoined twin-cages”.^208,209^ They were further able to control which species formed, either a Pd^II^_12_L_6_ cage with three mechanically coupled cavities or two helically isomeric Pd^II^_6_L_3_ cages, by the judicious choice of assembly conditions.^208^

### 4.4 Flexible Ligands Containing More than One Type of Coordinating Motif

**Figure 36:**
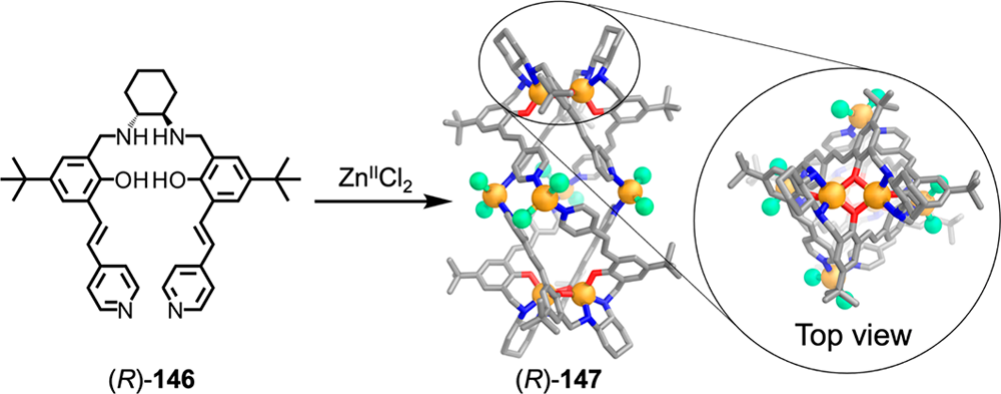
Assembly of octanuclear helicate **147** consisting of two bowl-like Zn^II^_2_**146**_2_ dimers linked by four equatorial Zn^II^Cl_2_ units.^210^

This section considers flexible ligands that bind metal centers using more than one type of donor atom or binding moiety incorporated into the same ligand. Octanuclear helicate **147** (Figure 36), with a cavity large enough to bind amino acids enantioselectively, exemplifies this approach.^210^ The combination of Zn^II^Cl_2_ and chiral salen-based ligand **146** produces **147**, which consists of two bowl-like Zn^II^_2_**146**_2_ dimers linked by four equatorial zinc centers. Within each dimer, each five-coordinate zinc center is chelated by the N_2_O_2_ pocket of one of the ligands, and the two metal centers are linked by two phenalato oxygen atoms. The two pendent pyridyl groups of each ligand remain free to coordinate to additional Zn^II^ ions, whose tetrahedral geometries are satisfied by coordination of two chloride ions, resulting in the formation of the Zn^II^_8_**146**_4_Cl_8_ structure **147**. The use of enantiopure ligand **146** is essential for the formation of cagelike helicate **147**. The use of racemic **146** results in the formation of dimeric units containing ligands with opposite handedness, which causes the four peripheral pyridyl groups to point toward different faces of the Zn^II^_2_ core, precluding helicate formation.

**Figure 37:**
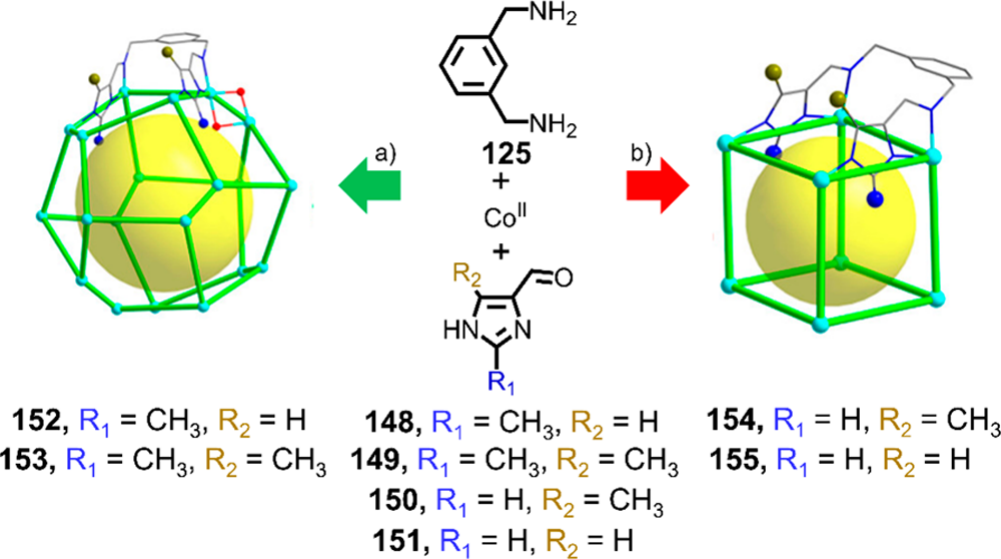
Subcomponent self-assembly of metal imidazolate (a) tetartoids and (b) cubes. The geometry of the assembled structure is governed by the steric properties of substituent R_1_. Adapted from ref (211). Copyright 2017 American Chemical Society.

Li et al. reported cobalt–imidazolate cage **152**, which assembles upon combination of 2-methyl-4-formylimidazole (**148**), *m*-xylylenediamine (**125**), and Co^II^.^211^ The 12 ligands form in situ and combine with 12 OH^–^ ions, four water molecules, four octahedral Co^III^ centers, four tetrahedral Co^II^ ions, and 12 distorted square-pyramidal Co^II^ centers to form a *T*-symmetric tetartoid structure (Figure 37a). Furthermore, the addition of d- or l-menthol during self-assembly yields enantiopure ΔΔΔΔ-**152** or ΛΛΛΛ-**152**, respectively. The imidazolyl 2-methyl substituent was an effective steric structure-directing feature. This methyl group points inside the pentagonal face of the structure, whereas it could not fit within the smaller window of a cube.

In contrast, when 5-methyl-4-formylimidazole (**150**) or 4-formylimidazole (**151**) combines with Co^II^ and *m*-xylylenediamine, cubic cage **154** or **155** (Figure 37b) forms; in **154**, the 5-methyl groups point away from the faces of the structure.^212^ Thus, the substituent at the 5-position of the imidazolyl ring does not exert steric control over the structure formed, in contrast with the 2-substituent. This work, together with Kwong’s (Figure 32),^197^ highlights the role that flexible subcomponents play in directing self-assembly. The same simple diamine subcomponent formed complexes with very different structures depending on the steric properties of other subcomponents within the system.

**Figure 38:**
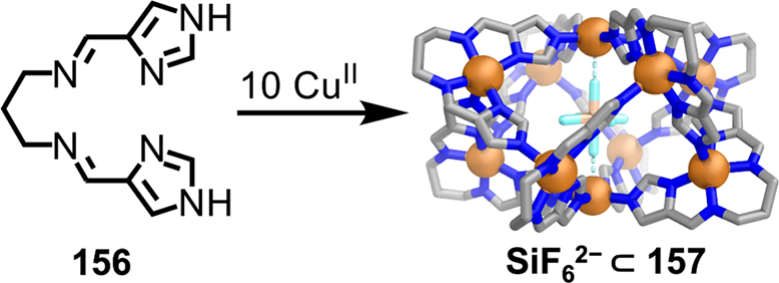
Self-assembly of adaptable Cu^II^_10_**156**_8_ bicapped square antiprism **157**.^213^

Li recently reported the use of a different flexible bis(imidazole) ligand, **156** (Figure 38), to form bicapped square-antiprismatic structure **157** upon reaction with Cu^II^ under solvothermal conditions (Figure 38).^213^ Single-crystal X-ray diffraction showed the formation of Cu^II^_10_**156**_8_ cages that have two types of Cu^II^ centers. Eight equatorial Cu^II^ ions have a distorted square-pyramidal geometry, with tetradentate chelation of one ligand and monodentate binding of a second. Each of the two axial Cu^II^ centers is bound by four imidazolate donors, with additional coordination of anions and water molecules to complete the coordination sphere.

Bicapped square-antiprismatic structure **157** can expand or compress vertically to accommodate different anions in its cavity because of its flexible ligands. Among the anions encapsulated (SiF_6_^2–^, ClO_4_^–^, Br^–^, and Cl^–^), SiF_6_^2–^ gives the largest cavity volume and Cl^–^ the smallest. Cage compression is triggered through anion exchange, for example, by the addition of KCl to a cage binding ClO_4_^–^ internally. Li et al. also employed ligand **156** to form a mixed-valence Cu^II^/Cu^I^ metallocycle.^214^ Upon combination of this metallocycle with triethylenediamine in a 2:3 ratio, a trigonal-prismatic structure forms.^215^ This trigonal prism undergoes a structural transformation to form **157** upon oxidation of Cu^I^ to Cu^II^.

### 4.5 Ligand Flexibility Arising from Substituent Positioning

An alternative way to introduce flexibility into ligands without incorporating alkyl or other flexible linkers is to vary the position of substitution of aryl rings or change the metal-binding moieties so as to provide multiple conformers capable of binding metal ions in different ways.

**Figure 39:**
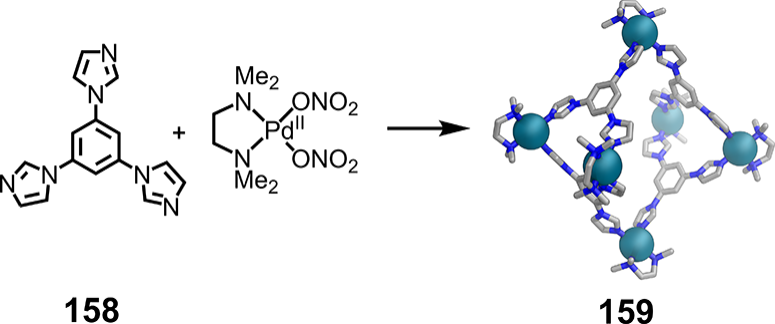
Self-assembly of open Pd^II^_6_**158**_4_ cage **159**.^216^

For example, tri- and tetratopic ligands that employ 3-pyridyl binding sites or imidazoles have been used in place of conformationally locked 4-pyridyl binding sites. When binding to *cis*-protected square-planar metal centers, such tritopic ligands can form M_6_L_4_ open cages (**159**)^216^ or bowl-like^217^ structures. Mukherjee et al. reported Pd^II^_6_**158**_4_ open cage **159**, which forms from tritopic ligand **158** with imidazole donor groups (Figure 39) and can catalyze Knoevenagel condensations and Diels–Alder reactions within its hydrophobic cavity in water.^216^ Recently Klajn and co-workers adopted this cage for the investigation of photoswitching in confined environments.^218−221^

Analogous tetratopic ligands have been shown to form M_6_L_3_ trifacial^222,223^ and M_8_L_4_ tetrafacial^64,224^ barrels that are structurally similar to those formed using the elongated tetratopic ligands discussed in section 7.2. A similar type of ligand flexibility was employed by Schröder and co-workers to form a Cd_66_ nanosphere with idealized *T* symmetry. Its dual-shell structure consists of a sphere of 66 Cd^II^ centers bridged by μ^3^-hydroxide, μ^3^-oxo, and μ^5^-NO_3_^–^ anions and enclosed by 12 DMF ligands and 20 tritopic organic capping ligands.^225^

### 4.6 Flexible Pseudolinear Polypyridyl Ligands

**Figure 40:**
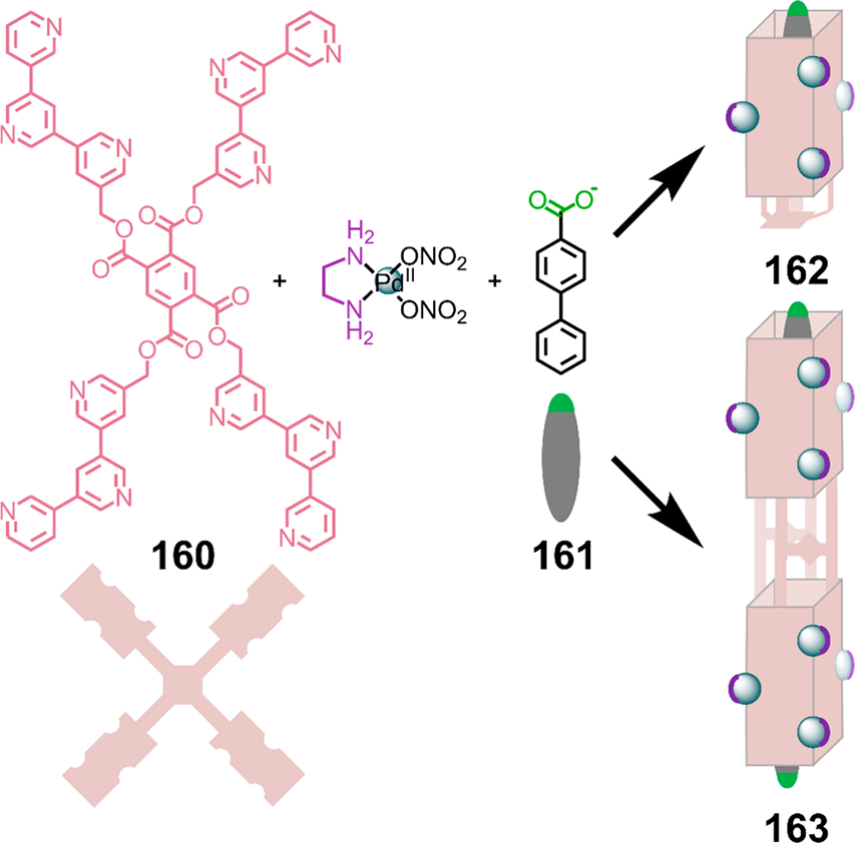
Flexible ligand **160** forms Pd^II^_6_**160** nanotube **162** and Pd^II^_12_**160**_2_ nanotube **163**.^226^

Fujita and co-workers have demonstrated that in addition to the formation of nanotubes from relatively rigid polypyridyl ligands through guest templation (section 7.4), nanotubular structures are also obtained using more flexible ligands. The combination of Pd^II^(en)(NO_3_)_2_ with ligand **160** (Figure 40) and a rodlike guest template results in the formation of Pd^II^_6_**160** end-capped tube **162**.^226^ The flexible nature of the benzenetetracarboxylate-containing core of the ligand allows it to fold and form structure **162** containing only one folded ligand. Selective guest binding within this tube was observed, whereby a biphenylcarboxylate guest bound unidirectionally with the biphenyl group ensconced in the hydrophobic pocket of the tube and the hydrophilic carboxylate exposed to the solvent.

At higher concentrations, the longer Pd^II^_12_**160**_2_ tube **163** forms as a minor species and was isolated via crystallization. X-ray crystallography revealed a doubly open-ended tube that is 3 nm in length with two template molecules residing inside the cavity.

Similarly, Chand et al. showed that a flexible pseudolinear tripyridine ligand forms a Pd^II^_3_L_4_ double-decker cage.^227^ Upon reduction of the metal:ligand ratio from 3:4 to 1:2, the ligand reconfigures into a U-shaped conformation in which the two terminal pyridines bind to the same Pd^II^ center to form a Pd^II^L_2_ spiro-type complex and the central pyridine donor of each ligand remains uncoordinated. Interconversion between the two structure types occurred following alteration of the metal:ligand ratio of the reaction mixture.

A consistent theme for this section is that the structures formed from flexible ligands can be difficult to reliably predict, meaning that the results are often serendipitous. However, as elsewhere, rules and hypotheses derived from these initial observations can enable the design of related structures and components to selectively form a desired structure that may have initially been observed to form as one component of a mixture. The adaptability exhibited by some structures formed using flexible ligands is more rarely observed for structures formed with more rigid ligands. The reconfiguration of these more flexible structures can lead to new functions, often related to guest binding.

## 5 Complexity Derived from Solvent, Anions, and Templates

Changes in the environments where metal–organic cages form can alter the structure formed. The course of self-assembly may be reconfigured by changing the solvent, adding a guest, or manipulating external conditions such as temperature and concentration. Although the effects of the environment on the structure may be challenging to predict beforehand, they may be rationalized, and the resulting knowledge can again be used to infer self-assembly rules. In this section we review techniques used to generate complex architectures from simple subcomponents by modulation of the external conditions and by guest addition.

### 5.1 Solvent- and Concentration-Dependent Complexity

**Figure 41:**
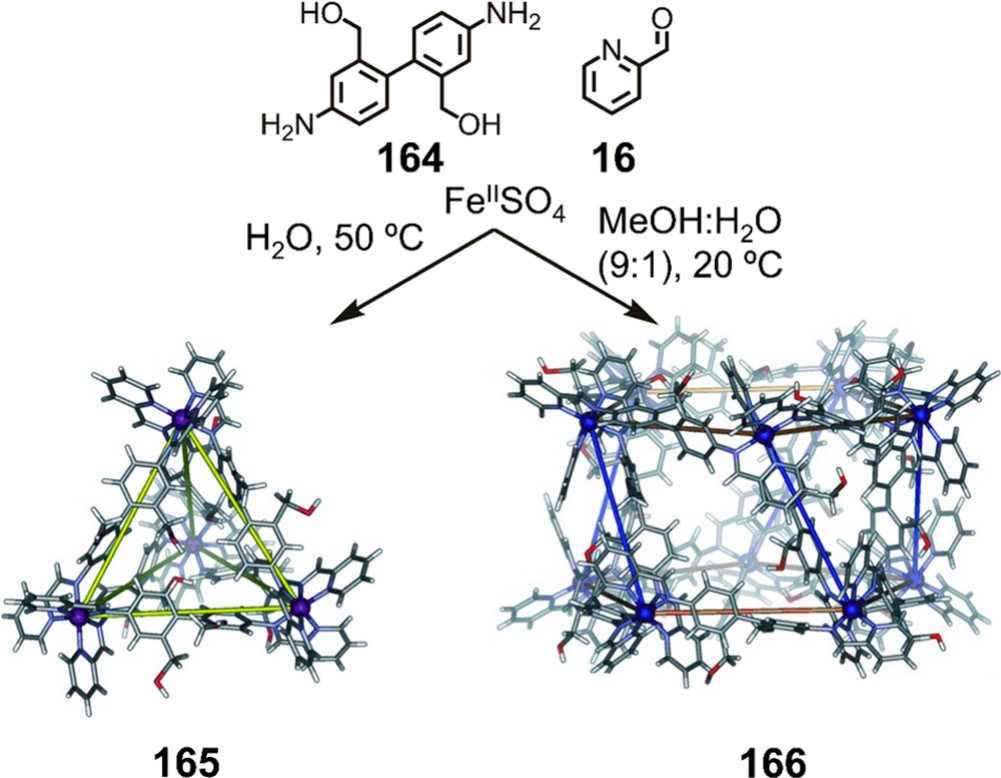
Formation of a self-assembled tetrahedron (**165**) and pentagonal antiprism (**166**), controlled by solvent polarity. Adapted with permission from ref (228). Copyright 2013 Wiley-VCH Verlag GmbH & Co. KGaA, Weinheim.

One of the most straightforward methods to direct the assembly of complex architectures is to vary the solvent used. We reported a system where tetrahedral metal–organic cage **165** forms in water but a mixture of methanol and water leads to the selective formation of pentagonal antiprism **166** instead (Figure 41).^228^ By tuning of the temperature in addition to the solvent, either architecture can be prepared exclusively, with lower temperatures favoring the pentagonal antiprism. This process involves a switch from *fac* coordination stereochemistry around the metal centers in the tetrahedral cage to all *mer* metal centers in the pentagonal antiprism, where the lower-symmetry *mer* coordinative linkages give rise to increased structural complexity.^229−232^ Antiprism **166** is kinetically stable toward changes in the solvent, requiring heating for a week to convert to the thermodynamically preferred tetrahedron **165** following solvent exchange. However, the pentagonal antiprism is responsive to the addition of a competing aniline: addition of 4-methoxyaniline to a mixture of pentagonal antiprism **166** and tetrahedron **165** brings about the selective disassembly of **166**. Severin and co-workers were able to trigger the rearrangement of an octanuclear prismatic cage that forms in chloroform into a tetranuclear complex by exchanging the chloroform solvent for dichloromethane.^233^ This work illustrates how even quite subtle changes in the solvent can trigger substantial transformations in the assembly structure, especially when the structures have specific binding interactions with that solvent.

**Figure 42:**
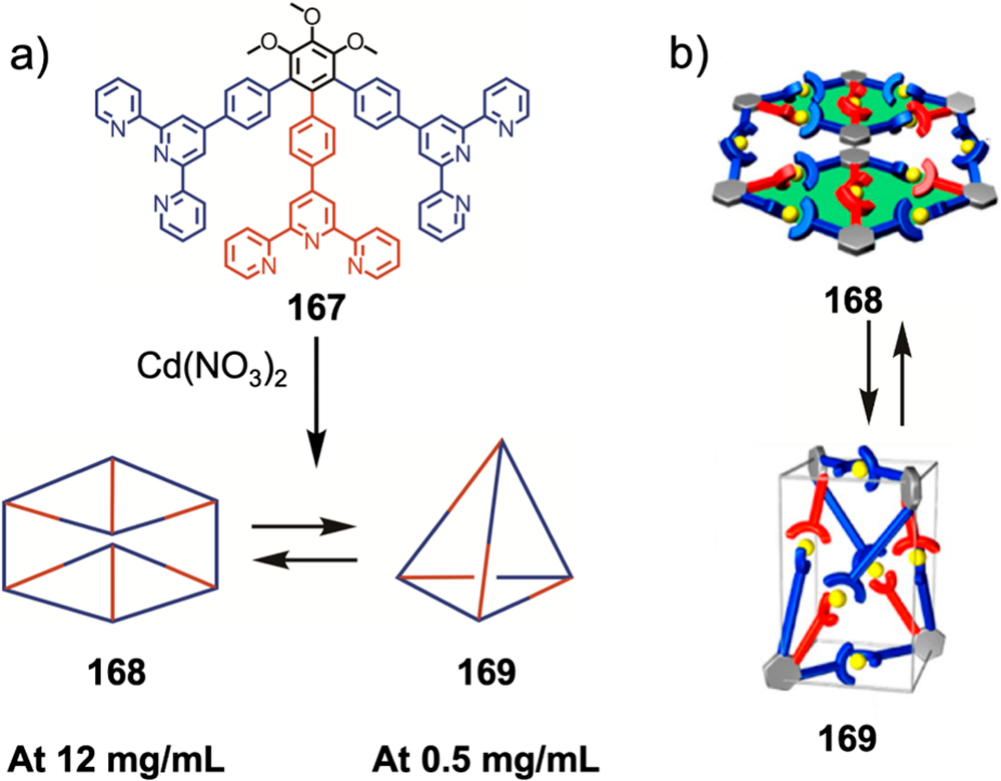
(a) Formation and switching between bisrhombohedral complex **168** and tetrahedron **169** in a concentration-dependent process. (b) Schematic view. Adapted from ref (234). Copyright 2014 American Chemical Society.

Newkome, Wesdemiotis, and co-workers reported an assembly process where at higher concentrations ligand **167** assembles with Cd^II^ to produce bisrhombohedral structure **168**, whereas at lower concentrations the simpler tetrahedron **169** is favored (Figure 42).^234^ The use of tris(terpyridine) ligands with Cd^II^ provided the delicate balance of lability and stability required for these structures to form. At higher concentrations, the bisrhombohedral architecture forms exclusively, whereas the tetrahedron is the exclusive product at lower concentrations. To confirm the structure of the tetrahedron, which could not be isolated because of rapid equilibration back to the bisrhombohedral architecture, a ruthenium(II)-containing metalloligand, essentially consisting of two units of **167** connected by the coordination of one (blue) terpyridine unit on each **167** to a kinetically inert ruthenium(II) center, was employed. The formation of a similar tetrahedral architecture confirmed the structural assignment of **169**. The authors subsequently reported a system wherein the predominant product among three architectures—a cuboctahedron, an octahedron, and a triangular sandwich complex—depended upon the concentration.^235^ These systems provide a way to generate and switch between complex architectures selectively in solution through manipulation of the concentration.

**Figure 43:**
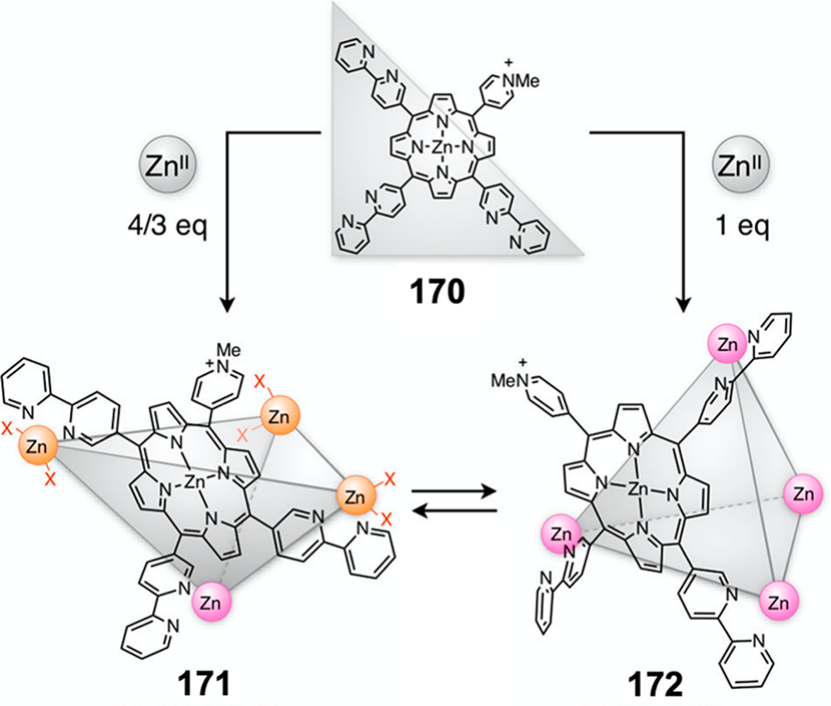
Formation of bowl **171** or pseudotetrahedron **172** from tris(bipyridyl)porphyrin **170**. Reproduced with permission from ref (236). Copyright 2019 American Chemical Society.

Shionoya and co-workers reported that tritopic ligand **170** forms bowl-like structure **171** and pseudotetrahedron **172** (Figure 43).^236^ Conversion between **171** and **172** is governed by different stimuli. Changes in solvent, metal:ligand stoichiometry, guest addition, or pH lead to the formation of one architecture over the other. For example, increasing the proportion of water in the acetonitrile solvent leads to selective formation of capsule **172** from bowl **171**, and addition of zinc triflate to a solution of **172** produces **171**.

Shionoya’s group reported the use of a similar ligand with fourfold symmetry in combination with zinc triflate and a mixed solvent system to generate an unusual *D*_3_-symmetric enneahedron.^237^ We have also made use of solvent effects in a system where a tetrahedron interconverted with dimeric and trimeric stacked structures on the basis of different chemical stimuli.^238^ Analogous stacked structures had previously been generated from a more rigid, achiral subcomponent, where interconversion between double and triple stacks was controlled by subcomponent substitution.^239^

### 5.2 Temperature-Dependent Assembly

**Figure 44:**
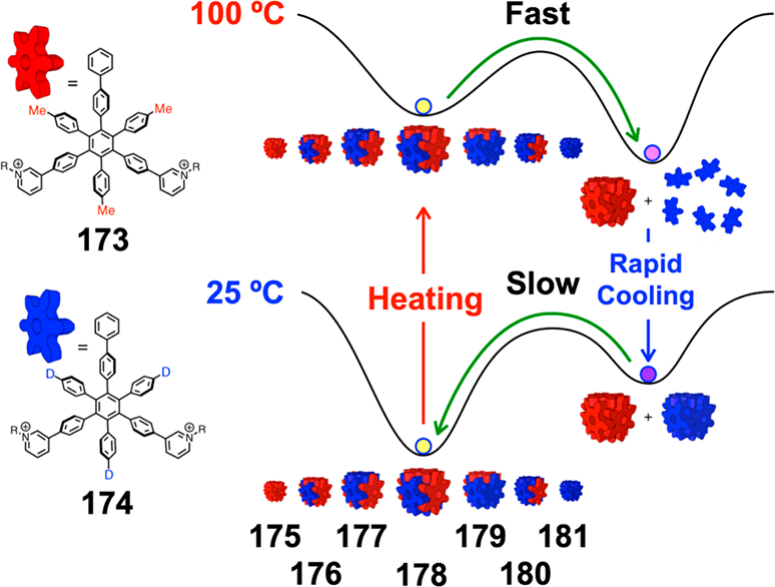
Temperature-dependent self-sorting and scrambling behavior driven by quenching equilibria. R = CH_3_. From ref (240). CC BY 4.0.

Hiraoka and co-workers reported the intriguing system shown in Figure 44, where two similar building blocks sort with a selectivity that varies as a function of temperature.^240^ Although these capsules are purely organic (Figure 44) and are held together by van der Waals forces, cation−π interactions, and the hydrophobic effect, their novel mechanism of sorting warrants inclusion in this review. The authors used gear-shaped amphiphilic molecules **173** and **174** with hexaphenylbenzene cores that self-assemble to form hexameric cubic architectures. These two hexaphenylbenzenes differ in the presence (**173**) or absence (**174**) of methyl groups at three positions (Figure 44). These additional methyl groups have a significant effect on the thermal stability of the formed hexameric architectures, with assembly **175**, composed of trimethylated **173**, dissociating at 130 °C, whereas assembly **181**, formed from non-methylated **174**, dissociates at 65 °C. When **173** and **174** were mixed and the mixture was allowed to equilibrate at room temperature in water, a statistical distribution of capsules **175**–**181** containing both panels was formed as a result of the structural similarities between them. The authors then heated the scrambled system above the disassembly temperature of **181**, which led to survival of only **175** at 100 °C. The authors then quenched the system by rapid cooling, trapping it in a metastable state consisting of only the two homogeneous capsules **175** and **181**. These capsules then reequilibrated, taking 2 days at 25 °C to reach the equilibrium state of a statistical distribution. This process could be further controlled by binding of guests in the cavities of these self-assembled architectures. This work provides a unique example of control over the statistical distribution of a system of capsules using temperature-quenching techniques analogous to those used in metallurgy.

### 5.3 Guest-Templated Assembly

**Figure 45:**
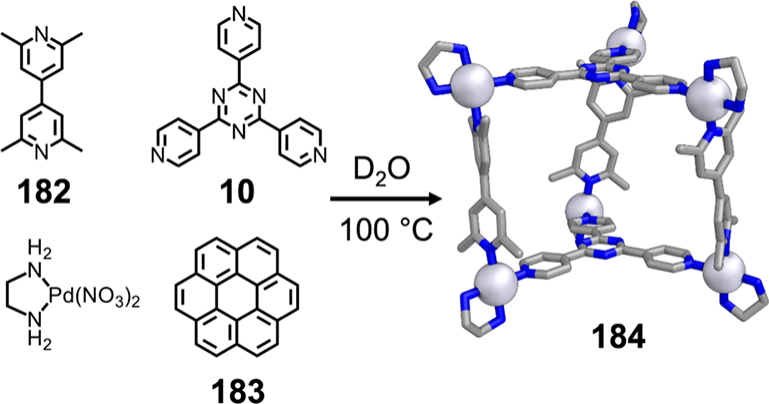
Self-assembly of ditopic **182** and tritopic **10** with (ethylenediamine)Pd^II^(NO_3_)_2_ to form guest-templated trigonal prism **184**. For clarity, the two bound coronene (**183**) guests are not shown.^241^

Fujita and co-workers prepared self-assembled heteroleptic trigonal prism **184** (Figure 45), the assembly of which required the presence of certain π-extended guest molecules.^241^ When no guest was present, oligomeric species and homoleptic octahedral [(ethylenediamine)Pd^II^]_6_**10**_4_ predominated. The selective formation of heteroleptic **184** is driven by aromatic stacking interactions between electron-poor cage panels (**10**) and coronene (**183**) and also by the steric bulk of the linear bipyridine strut **182**, which disfavors the coordination of two linear bipyridine ligands to a single palladium center. When the bipyridine struts are extended, allowing the formation of larger trigonal prisms, three guest molecules stack selectively within the capsule cavity. Both capsules, containing two or three guests, were shown by UV/vis spectroscopy to exhibit charge-transfer character.

The Fujita group has also reported systems of capsules formed from Pd^II^ with *cis*-chelating bidentate ligands and pyridine or pyrimidine donor ligands, where guest binding triggers structural transformations. In one example, a trigonal-bipyramidal cage transforms into an octahedral architecture upon guest binding.^242^ In another, an octahedral Pd^II^_20_L_8_ structure transforms to an open Pd^II^_8_L_4_ bowl-shaped structure.^243^ Further to this, Hiraoka and Fujita were able to control the specific constitutional isomer of a Pd_3_L_2_ complex that forms from a reduced-symmetry tripyridyl ligand by judicious choice of guest molecules.^244^ Addition of a flat guest molecule (1,3,5-benzenetricarboxylic acid) favored the formation of a capsule with a relatively flat cavity, whereas the addition of a spherical guest (CBrCl_3_) resulted in the formation of an isomer with a more spherical cavity, providing an early example of guest control of isomer formation. We reported the transformation of a tetrahedral Fe^II^_4_L_6_ cage with porphyrin panel edges upon the addition of C_60_ or C_70_. After addition of the fullerene to the system, a Fe^II^_3_L_4_ conelike architecture formed. Selective transmetalation of a single iron(II) vertex for copper(I) was favored, resulting in the formation of a heterometallic Cu^I^Fe^II^_2_L_4_ structure, exploiting the coordinative unsaturation of iron(II) at a single position.^245^ Our group also reported the formation of a cuboctahedron that shows cooperative binding of a pair of C_60_ molecules. The binding of these guests triggers a rearrangement of the original *O*-symmetric structure to an *S*_6_-symmetric analogue that optimizes fullerene binding.^246^

Müller and Möller reported the formation of a trigonal-bipyramidal capsule that incorporated its template.^247^ Sodium 5,5-diethylbarbiturate was added to a threefold-symmetric guanidinium-based ligand that chelated three Pd^II^ centers, leading to the formation of a trigonal-bipyramidal architecture containing 33 distinct building blocks. While investigating the endohedral functionalization of metal–organic polyhedra via coordination of organophosphonates to polyoxovanadate units at the vertices of the cages, Fang et al. observed the formation of a barrel-like structure instead of a tetrahedral structure that would ordinarily be expected to form.^248^ The observed preference for the barrel-like structure was attributed to steric effects, as the interior cavity of the tetrahedron would be too small to accommodate the sterically bulky organophosphonate groups. Donnelly, Abrahams, Paterson, and co-workers likewise reported the guest-induced formation of coordination nanotubes that can bind small molecules such as CO_2_, CS_2_, and acetonitrile.^249^

**Figure 46:**
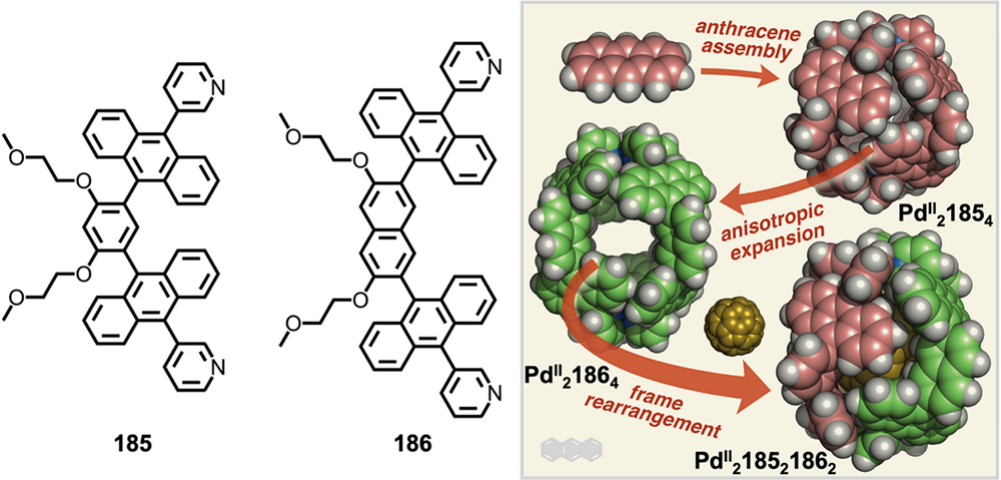
Formation of a heteroleptic lantern driven by guest encapsulation. Reproduced with permission from ref (250). Copyright 2015 Wiley-VCH Verlag GmbH & Co. KGaA, Weinheim.

The Yoshizawa group reported a modification of their anthracene-paneled M_2_L_4_ lantern architectures wherein guest binding drives the formation of heteroleptic structures (Figure 46).^250^ They used two pyridine-based bidentate ligands of different lengths, **185** and **186**. Each ligand independently assembled with the metal ion to form an M_2_L_4_ lantern architecture with spherical internal cavities capable of binding aromatic guests. When the two cages were mixed together, a complex mixture of hetero- and homoleptic architectures formed. However, when C_60_ was added to this mixture, a single heteroleptic host–guest species was seen, with the composition Pd^II^_2_**185**_2_**186**_2_⊂(C_60_). Computational models suggested that the *cis* isomer of this architecture is favored over the *trans* isomer. The observed preference for the heteroleptic architecture was attributed to the optimization of aromatic stacking interactions between the guest and host.

This guest-driven host rearrangement concept might be employed to selectively generate a range of architectures tailored to specific guest-binding tasks. In this approach, the cavity of the host is already filled, thus precluding its use for the binding of guests with lower affinity than the template or necessitating the optimization of template removal. Yoshizawa and co-workers recently reported the use of a guest template to drive a system of equilibrating atropisomeric cages toward a single isomer in a similar system.^251^

### 5.4 Anion-Templated Assembly

**Figure 47:**
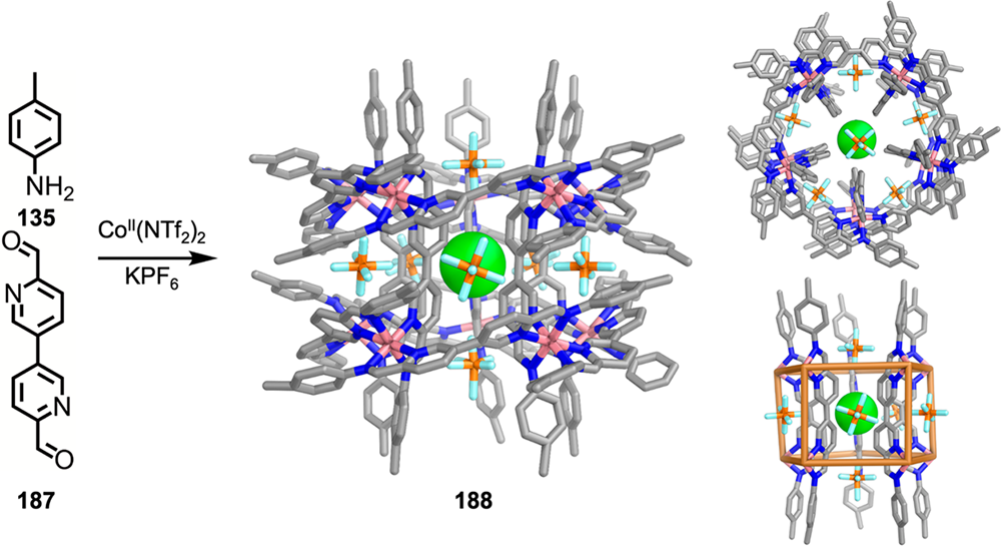
Formation of anion-templated pentagonal prism **188**.^252^

We reported a system of complex architectures based on anion binding and subcomponent self-assembly (Figure 47).^252^ Diformylbipyridine subcomponent **187** combines with *p*-toluidine (**135**) and cobalt(II) triflimide to generate an initial mixture of architectures. The addition of a triflate template leads to the assembly of a Co^II^_4_L_6_ tetrahedron. However, when lithium perchlorate or potassium hexafluorophosphate is added to either the dynamic library or the tetrahedral architecture, clean conversion to pentagonal prism **188** (Figure 47) is observed upon heating.

Co^II^_10_L_15_ architecture **188** consists of two parallel Co^II^_5_L_5_ pentagonal rings connected by five bridging ligands, creating a barrel-like structure. This structure is templated by five perchlorate or hexafluorophosphate anions in binding pockets between pairs of bridging ligands. Forming a pentagonal prism in place of the initial tetrahedral structure is favored for reasons that include the maximization of stacking interactions and a better size match of perchlorate or hexafluorophosphate with the smaller cavities found in the pentagonal prism.

The formation of pentagonal prism **188** also generates a central binding pocket surrounded by 10 internally facing pyridine CH groups. This pocket is occupied by a chloride ion, scavenged during synthesis, which is so strongly bound that it cannot be removed by addition of silver(I), in analogous fashion to other self-assembled systems found to bind strongly to halides.^253−255^ This system thus provides an unusual way to generate a secondary anion binding site by the addition of an initial anionic stimulus. Further work explored different architectures formed by subcomponent self-assembly using this same dialdehyde **187**.^256−259^

Chifotides and Dunbar reported the use of anion−π interactions to control the formation of tetrameric or pentameric helicates, depending on the identity of the anion used.^260^ The choice of anion during self-assembly likewise dictates the identity of the product formed in the work of Pan, Xu, and co-workers, where the formation of either a Pd^II^_2_L_4_ or a Pd^II^_3_L_6_ capsule is driven by the addition of nitrate (favoring Pd^II^_2_L_4_) or triflate/tetrafluoroborate (favoring Pd^II^_3_L_6_).^261^ This selectivity is driven by differential guest binding within each capsule. Lützen and co-workers also reported a chiral Pd^II^_4_L_8_ flexible architecture whose formation is dependent on templation by tetrafluoroborate.^262^

## 6 Multimetallics: Heterometallic and Cluster-Containing Architectures

Complexity may be enhanced through the development of heterometallic self-assembled systems, which employ the differing coordination preferences of more than one metal. The development of systems that take advantage of the coordinational flexibility and unusual geometries of metal clusters can also lead to the formation of novel architectures. Either the kinetics or the thermodynamics of self-assembly may be employed to direct the outcome of a multistep process, as described in the examples below.

### 6.1 Ligand Coordination Preference

**Figure 48:**
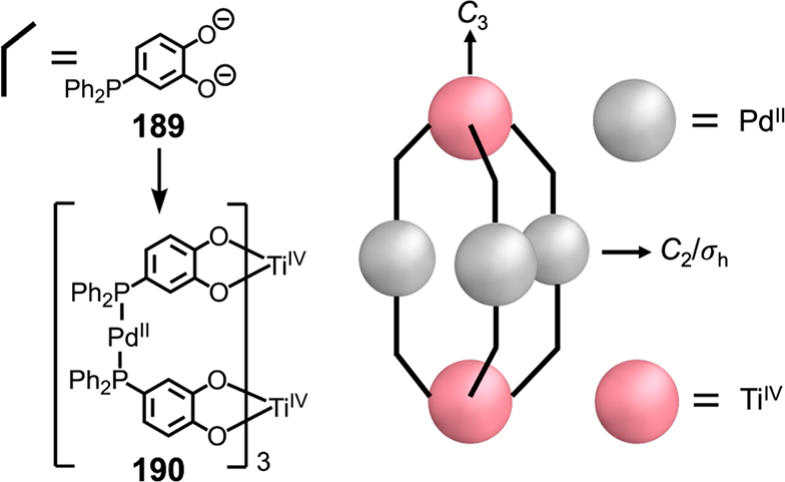
Heterobimetallic Ti^IV^_2_Pd^II^_3_**189**_6_ mesocate **190** formed using ligands that contain both hard and soft donors.^263,264^

An early example of heterometallic supramolecular assemblies was provided by Raymond and Wong, who used ligand **189** that contains both hard and soft donors (Figure 48) to selectively bind two different metal ions.^263,264^ The catechol group of ligand **189** binds to hard metal centers such as Ti^IV^ and Sn^IV^, and the phosphine binds to softer metal centers such as Pd^II^. A stepwise process was initially employed, first installing the catechol-binding metal, as insoluble polymers were observed when phosphine coordination was attempted first. However, under the optimized conditions Ti^IV^- and Sn^IV^-containing mesocates are generated in a single step via selective self-assembly. Similar principles using other ligand designs have been used by the groups of Wang,^265^ Duan,^266,267^ Brechin,^268^ and Youngs^269^ to generate other trigonal-bipyramidal assemblies.

**Figure 49:**
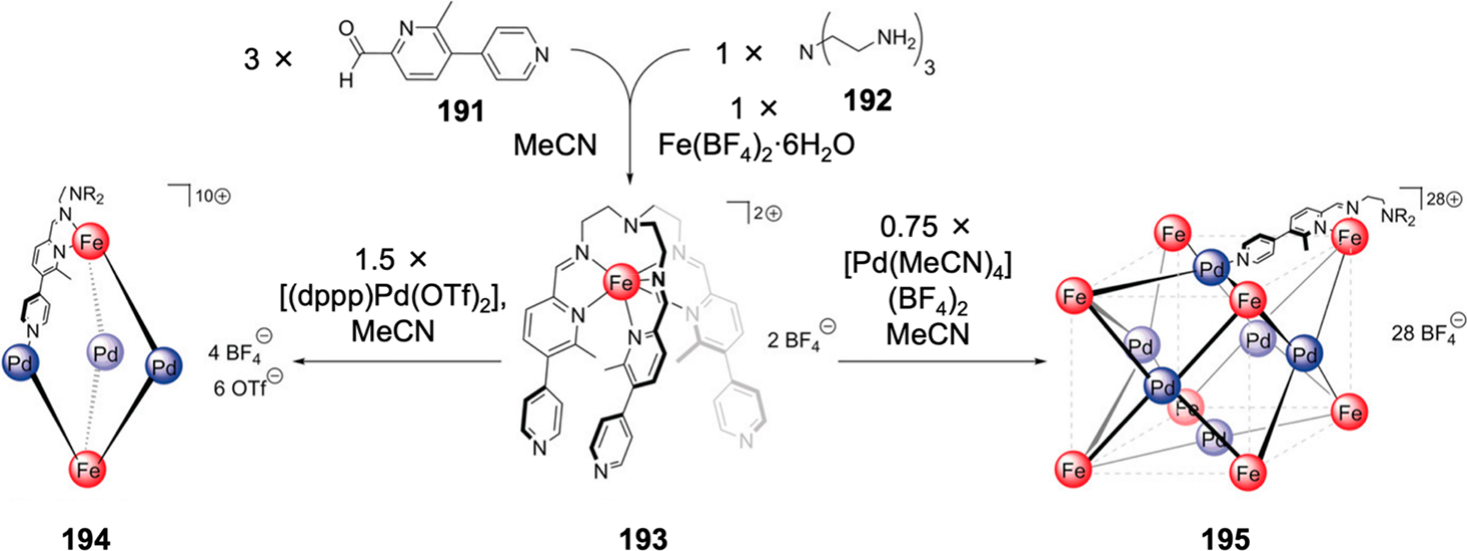
Self-assembly of subcomponents **191** and **192** with Fe^II^ to produce metalloligand **193**, which then forms bimetallic trigonal-bipyramidal (**194**) and cubic (**195**) architectures. Reproduced with permission from ref (270). Copyright 2019 Wiley-VCH Verlag GmbH & Co. KGaA, Weinheim.

Expanding upon the principles developed by Raymond and Wong,^263,264^ Lützen et al. reported a system incorporating both Fe^II^, which is selectively bound by pyridylimines, and Pd^II^, which binds selectively to monotopic pyridine donors, to form a system capable of complex-to-complex switching with a concomitant spin-state transition (Figure 49).^270^ Selective binding is driven both by intrinsic ligand preference (avoidance of steric clash at palladium centers ligated by pyridylimines) and by the preassembly of Fe^II^ metalloligand **193** using chelating tris(2-aminoethyl)amine (**192**). This chelating ligand enforces *fac* hexadentate coordination on the assembly. Subsequent addition of [(dppp)Pd^II^(OTf)_2_] (dppp = 1,3-bis(diphenylphosphino)propane) causes selective assembly of trigonal bipyramid **194**, where two coordination sites on each palladium are occupied by the bidentate phosphine ligand. Employing [Pd^II^(MeCN)_4_](BF_4_)_2_ instead generates cubic architecture **195**, which has a structure analogous to one that we prepared using a preassembled platinum complex metalloligand.^271^ When a less sterically hindered aldehyde subcomponent is added, the more hindered subcomponent **191** is displaced, and the iron(II) transitions from a high-spin state to a low-spin state.

Crowley and co-workers reported a nonanuclear heterometallic Pd^II^_3_Pt^II^_6_ donut-shaped cage, where the use of two different metal-binding sites on each ligand—bidentate triazolylpyridine versus monodentate pyridine—enables heterometallic assembly.^272^ The architecture catalyzes the light-mediated hetero-Diels–Alder reaction of anthracene with singlet oxygen.

### 6.2 Combining Kinetically Inert and Labile Metal Ions

**Figure 50:**
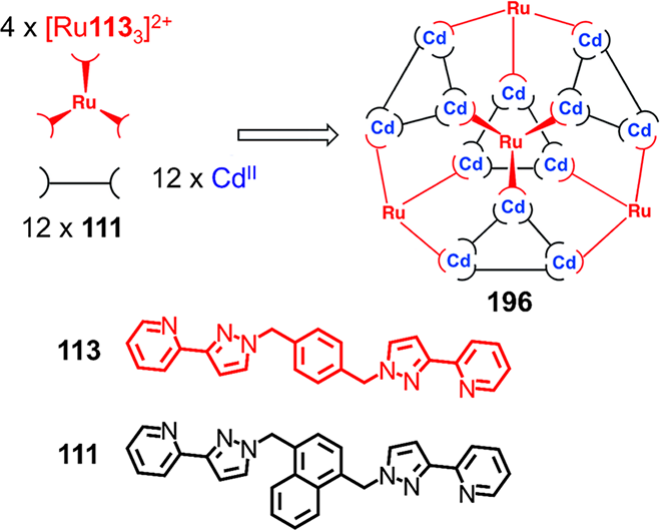
Formation of Ru^II^_4_Cd^II^_12_**111**_12_**113**_12_ twisted tetracapped truncated tetrahedral assembly **196** containing both kinetically inert and labile coordination centers and two distinct ligands. From ref (276). CC BY 3.0.

The use of a mixture of kinetically inert and labile coordination centers has been explored extensively by the Ward group (Figure 50).^273−276^ Their stepwise approach involves the initial formation of a coordination complex between three pyrazolylpyridine ligands and kinetically inert Ru^II^ or Os^II^. This complex is formed as a statistical mixture of *fac* and *mer* isomers, and the minor *fac* isomer is then isolated, exploiting the inertness of these metals.^277^ An example of this *fac* complex, *fac*-Ru**113**_3_, is represented in red in Figure 50. One coordinating site on each ligand is occupied by the Ru^II^, leaving the other free for coordination to a coordinationally labile metal center. Subsequent addition of labile Co^II^, Cd^II^, or Ag^I^ then results in self-assembly with efficient error checking, as the structures undergo coordinative reconfiguration about the labile metal ion.

The Ward group further developed this system to install two types of ligands selectively into a heterometallic architecture to form Ru^II^_4_Cd^II^_12_**111**_12_**113**_12_ twisted tetracapped truncated tetrahedral array **196** (Figure 50).^276^ The design of this structure builds upon the homometallic M_16_L_24_ structures previously reported by the same group, as depicted in Figure 31a.^191^

The Jin group also reported a series of heterometallic capsules that combine kinetically inert metals such as rhodium and iridium with kinetically labile metals such as silver and zinc.^278^

### 6.3 Multimetallic Vertices

**Figure 51:**
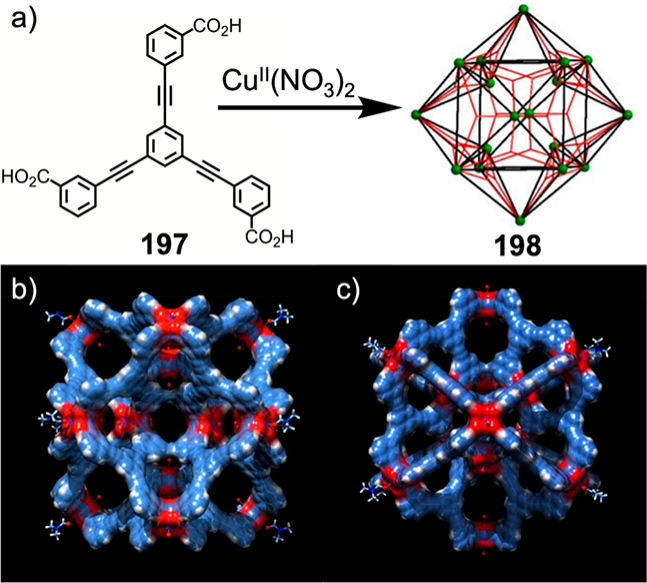
(a) Formation of “capsule-within-a-capsule” **198**, composed of an octahedron nested within a cuboctahedron. (b, c) Views of **198** from two perspectives. From ref (281). CC BY 4.0.

Dicopper “paddlewheel” vertices have been used to generate an array of molecular capsules.^279,280^ In an elegant example, Schmitt and co-workers generated “capsule-within-a-capsule” **198** using dicopper paddlewheel complexes as nodes (Figure 51).^281^ Extended tri-*m*-benzoic acid ligand **197**, once deprotonated, reacts with Cu^II^(NO_3_)_2_ to form **198**. X-ray crystallography revealed a complex octahedron-within-cuboctahedron architecture, with the inner assembly fully linked to the outer one. The architecture, which can be made soluble by postassembly modification with alkylpyridine donors, can absorb 7-amino-4-methylcoumarin from solution.

**Figure 52:**
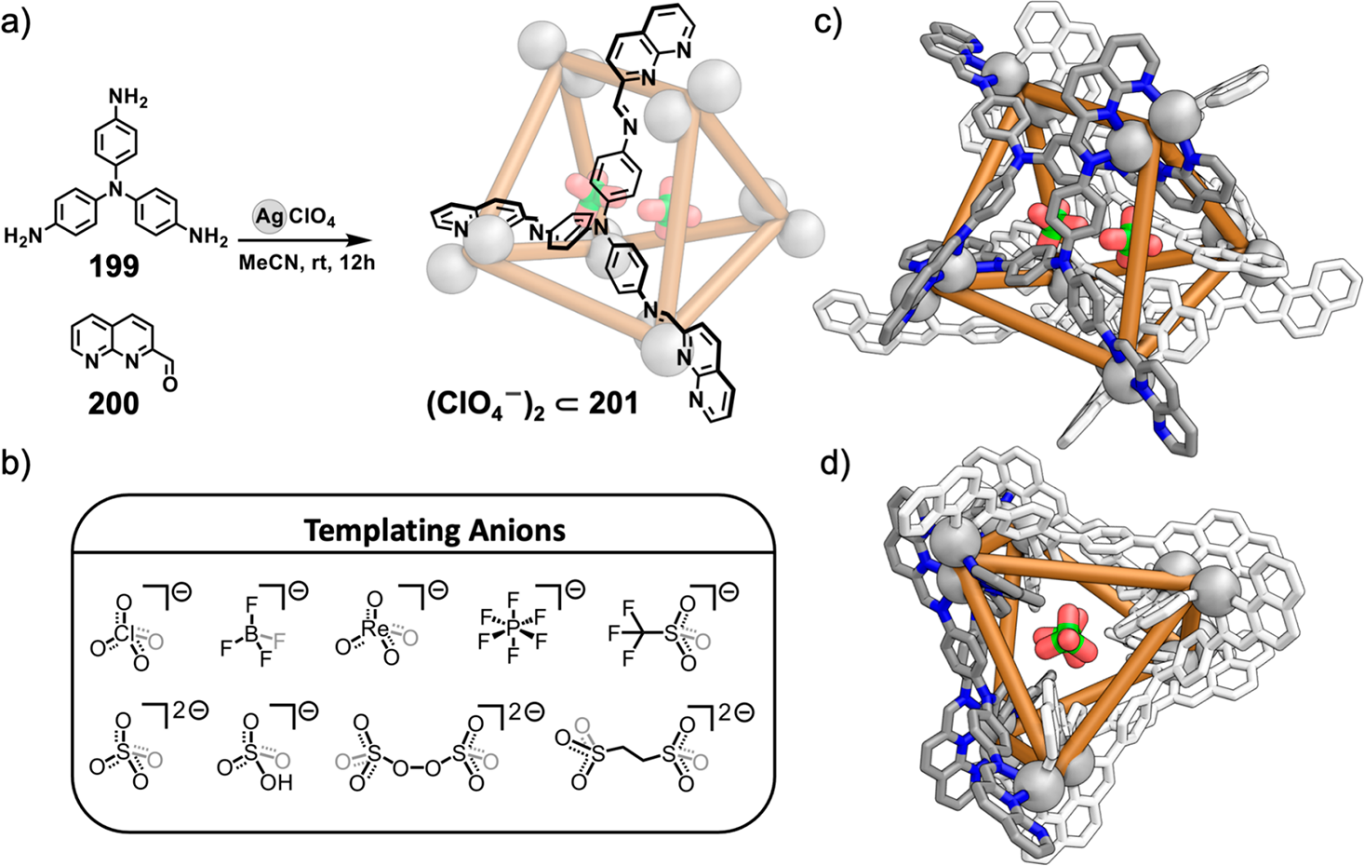
(a) Formation of trigonal prism **201** from silver(I) and subcomponents **199** and **200**, which binds (b) pairs of anions, or dianions, driven by the coordinational flexibility of silver(I) and anion templation. (c, d) Two views of the crystal structure of **201**. Reproduced from ref (282). Copyright 2019 American Chemical Society.

The potential of using coordinationally flexible bimetallic clusters was recently highlighted by our group in the assembly of a trigonal-prismatic cage that incorporates disilver vertices (Figure 52).^282^ The nonconverging coordination vectors of 2-formyl-1,8-napthyridine (**200**) combined with the flexible coordination sphere of silver(I) leads to the formation of disilver vertices. The geometry of tris(4-aminophenyl)amine (**199**) and an appropriate anionic template (Figure 52b) generate Ag^I^_12_L_6_ trigonal prism **201**.

Crystallographic analysis of **201** showed that two anions are bound within its cavity, held in proximity by the surrounding metal–organic architecture. Two HSO_4_^–^ anions bind in close proximity, stabilized by additional hydrogen-bonding interactions, as seen in the cyanostars of Flood and colleagues.^283^ Linear, covalently linked dianions also serve as competent templates for the trigonal prism. Even highly oxidizing species such as peroxodisulfate, which is known to oxidize Ag^I^ to Ag^II^,^284^ can be used, demonstrating the power of self-assembly to alter the properties of structural subunits.^2,35,62,285^ Silver clusters have also been used to generate a Ag_180_ nanocage with a diameter of 2.5 nm based on silver “trigons” (three silver ions in a triangular arrangement).^286^

**Figure 53:**
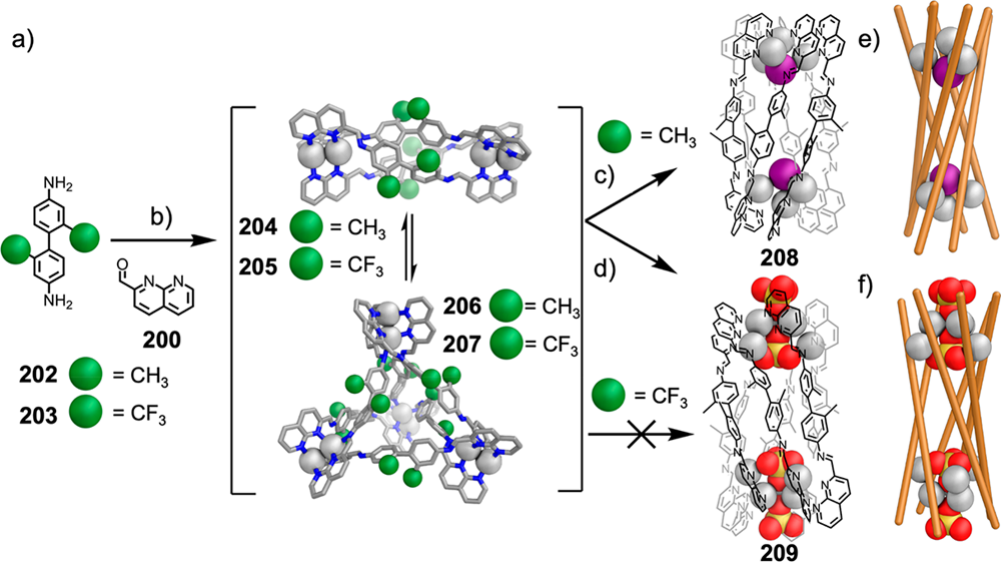
(a–d) Synthesis of six-stranded helicates **208** and **209** capped by Ag_4_ or Ag_6_ clusters, formed during self-assembly from dianiline **202** but not from dianiline **203**: (a) structures of **202** and **203**; (b) formation of **204**–**207** upon addition of AgNTf_2_ in MeCN; (c, d) formation of (c) **208** and (d) **209** from **204**/**206** upon addition of tetrabutylammonium iodide and tetramethylammonium sulfate, respectively. The structures of **204** and **206** are MM3 models, and those of **208** and **209** are based on crystallographic data. (e, f) Simplified representation of six-stranded helicates (e) **208** and (f) **209**. Adapted from ref (287). Copyright 2020 American Chemical Society.

We further extended this concept to incorporate not just bimetallic corners but also Ag^I^_4_ and Ag^I^_6_ clusters as integral structure-directing motifs using the ditopic subcomponent **202**.^287^ As shown in Figure 53b, **202** and **203** initially form mixtures of tetrahedra and three-stranded helicates (**204**–**207**). The mixture of **204** and **206** then proceeds to generate six-stranded helicates **208** and **209** in the presence of suitable anionic templates (Figure 53c,d), while analogous structures do not form from subcomponent **203**. Key to the formation of these unusual structures is the judicious choice of anion. Whereas the addition of other anions to the equilibrating mixture of **204** and **206** does not effect structural transformation, addition of iodide, bromide, or sulfate triggers rearrangement to a six-stranded helicate that resembles a sheaf of wheat. Its elongated structure was confirmed by X-ray crystallography and solution NMR spectroscopy. This work provides an unusual example of the mutual stabilization between metal clusters and a self-assembled architecture, with anions playing a central role in structuring the metal cluster and therefore the superstructure thus formed.^287^

Other metal clusters have been used as capsule vertices,^288^ including polyoxometalate-derived caps,^289^ which formed a capsule with an unusual “near-miss Johnson” geometry; tungsten–copper synthons, which enabled the formation of distorted octahedral structures;^290^ tripalladium vertices ligated by tetrazole linkers;^291^ and trizirconium clusters, which formed the corners of a chiral coordination cage capable of performing sequential asymmetric reactions.^292^ Manganese has also been employed to generate cages related to truncated tetrahedra.^293^

### 6.4 Organometallic Macrocyclic Tubes

**Figure 54:**
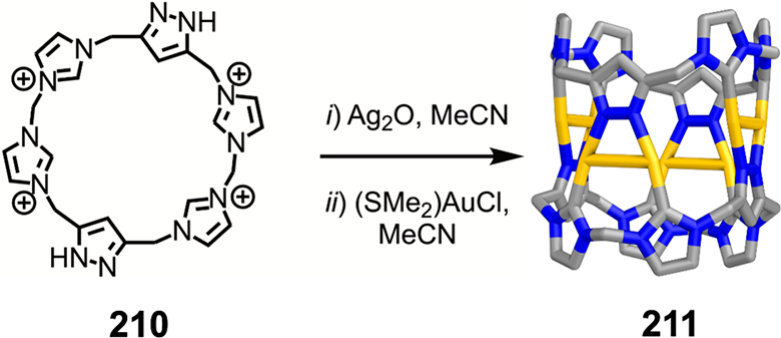
Formation of organometallic macrocyclic tubular structure **211**.^294^

Tubular assemblies may be generated by linking macrocyclic ligands with a band of metal centers, as exemplified by the work of Altmann and Pöthig (Figure 54),^294^ representing an alternate form of structure-directing metal cluster. The reaction of macrocycle **210**, containing four imidazolylidene and two pyrazolate rings, with silver(I) and then gold(I) ions leads to the formation of extended tube **211**. The cavity of **211** binds the linear guest 1,8-diaminooctane in organic and aqueous solutions. Architecture **211** exemplifies a novel approach to metal-driven self-assembly where the metal ions define a central ring rather than vertices. Such architectures have been used to form mechanically interlocked organometallic [2]rotaxanes.^295^ In a conceptually related example, Shionoya and co-workers had previously reported a Ag_3_L_2_ structure in which two ligand disks were linked by a ring of three silver ions, but solid disk-shaped ligands were used rather than macrocycles.^296^

Hetero- and multimetallic self-assembled structures have not been used as extensively as other techniques to generate complex architectures, but there is clearly great potential in this approach. A focus of future work will be utilizing the properties of the multiple metal ions to achieve tasks that cannot be achieved by a single ion. The use of metal clusters with coordinational flexibility as vertices likewise shows promise in the generation of complex architectures from simple ligands.

## 7 Geometric, Steric, and Subtle Non-covalent Effects

**Figure 55:**
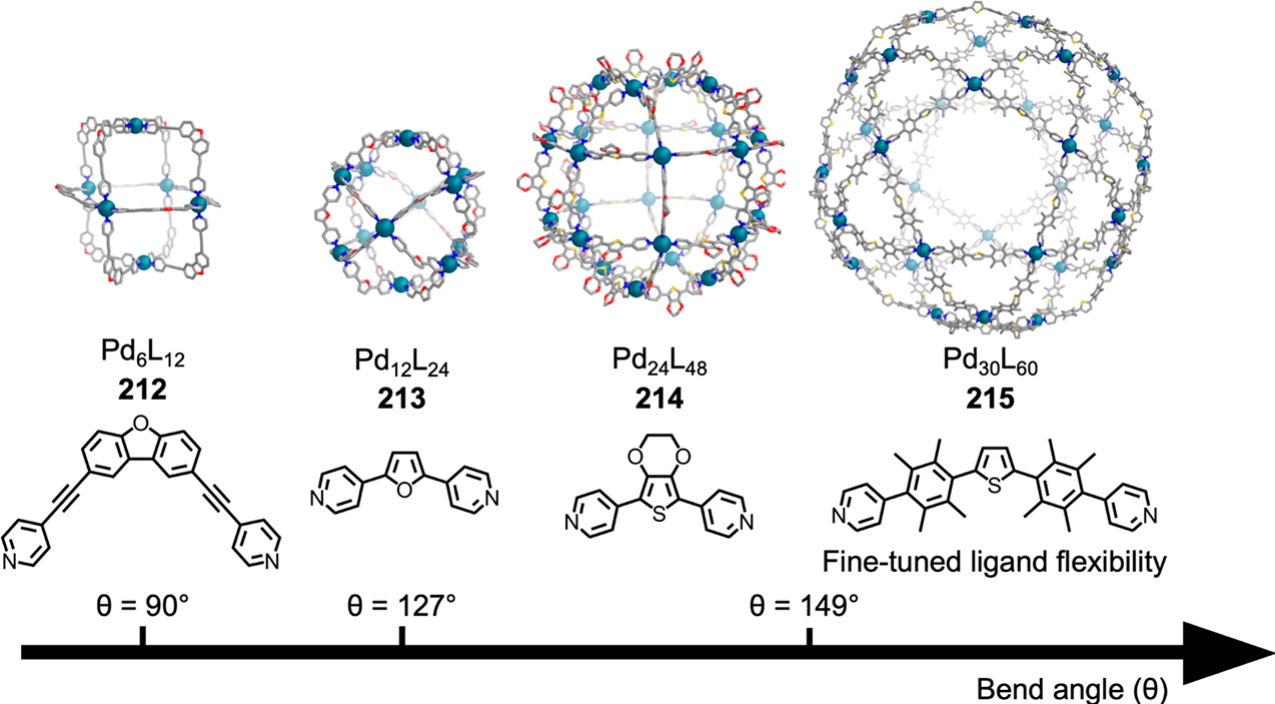
Family of spherical, polyhedral structures that Fujita et al. constructed by varying the bend angle, or flexibility, of ditopic “banana-shaped” dipyridine ligands.^31,156,297,298^

Geometric constraints can shape the self-assembly of high-symmetry structures. Key examples from the Fujita group have shown that even slight alterations of the bend angle (θ, Figure 55), or flexibility, of dipyridyl ligands can result in the formation of polyhedra with dramatically different sizes (Figure 55).^297,298^ The selective formation of tetrahedra or cubes also depends upon the relative orientations of the coordination vectors in bis-bidentate ligands.^28^ Similar geometric principles have been shown to drive the formation of more complex, lower-symmetry architectures and to enable discrimination between different structures of high complexity. This section will highlight instructive examples of geometric control along with cases demonstrating the impact of subtle steric effects and non-covalent interactions on the outcome of self-assembly processes.

### 7.1 Bend-Angle Dependence of Ditopic Struts

Key work by Fujita and others has demonstrated that myriad metal–organic structure types with the general formula M^II^_*n*_L_2*n*_ are formed through the self-assembly of dipyridyl ligands having different bend angles with square-planar Pd^II^ and Pt^II^ cations. These products are symmetrical polyhedra (Figure 55), including Pd^II^_6_L_12_ octahedron **212**,^31^ Pd^II^_12_L_24_ cuboctahedron **213**,^156^ Pd^II^_24_L_48_ rhombicuboctahedron **214**,^297,299^ and Pd^II^_30_L_60_ icosidodecahedron **215**.^298^ Higher-order structures are observed when ligands have a larger bend angle.^300,301^ However, other dipyridyl ligands have been shown to form architectures that deviate from these Platonic and Archimedean ideals.

**Figure 56:**
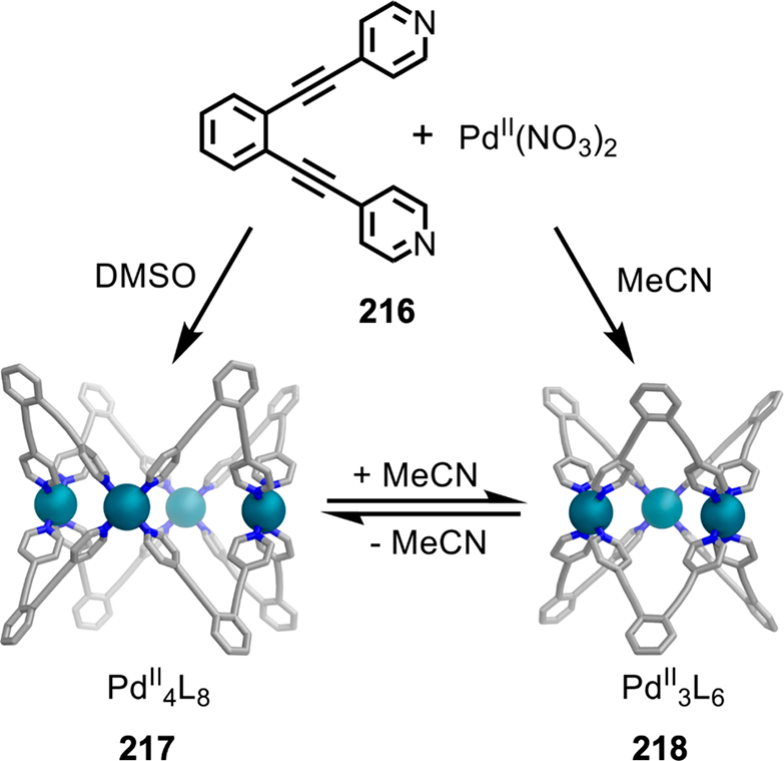
Solvent-dependent self-assembly of ditopic ligand **216** having a 60° bend angle and Pd^II^(NO_3_)_2_ to give Pd^II^_4_L_8_ (**217**) and Pd^II^_3_L_6_ (**218**) box-shaped structures.^302^

Fujita et al. demonstrated that dipyridyl ligand **216** (Figure 56), having a bend angle of 60°, assembles with Pd^II^(NO_3_)_2_ in DMSO to give Pd^II^_4_**216**_8_ box **217**.^302^ In contrast, carrying out the reaction in CD_3_CN results in the formation of smaller Pd_3_**216**_6_ tube **218**. The selective formation of **217** requires the presence of both DMSO and nitrate, with a mixture of **217** and **218** observed when Pd^II^(OTf)_2_ in DMSO is used. Finally, the ratio of **217** to **218** tracks the DMSO:MeCN ratio of the solvent mixture. Using similar principles, the Yoshizawa group obtained a Pd^II^_2_L_4_ polyaromatic capsule that was structurally contracted in comparison with a previously reported capsule using a similar ligand.^303^

**Figure 57:**
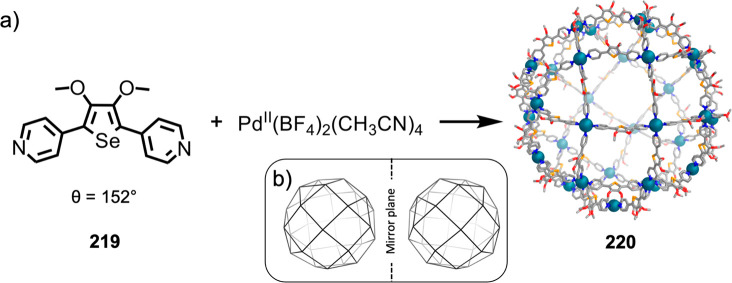
(a) Formation of metal–organic architecture **220**, corresponding to a chiral tetravalent Goldberg polyhedron. (b) Schematic representations of the two enantiomers of **220**.^304^

In targeting the next-largest structure in the series of regular Pd^II^_*n*_L_2*n*_ assemblies shown in Figure 55, a Pd^II^_60_L_120_ rhombicosidodecahedron, by widening the bend angle of the ditopic ligand, Fujita et al. instead formed an unexpected new architecture. Selenophene-centered ligand **219** (Figure 57) exhibits a bend angle of 152°, a modest increase upon the angle of 149° in the thiophene-centered ligands previously reported to assemble into Pd^II^_24_L_48_**214** and Pd^II^_30_L_60_**215** (Figure 55).^297−299^ The assembly of **219** and Pd^II^ in DMSO instead yields structure **220**, with a formula of Pd^II^_30_**219**_60_, the same composition as icosidodecahedral structure **215**.^304^

Refinement of X-ray crystallographic data through the extension of techniques originally developed for protein crystallography revealed that the surface of structure **220** is tiled by eight triangles and 24 squares. Fujita’s team developed a mathematical description of this new class of structures, which they named tetravalent Goldberg polyhedra. According to their nomenclature, **220** is a *tet*-G(2,1) polyhedron, with the numbers in parentheses describing the relative orientations and spacings of the triangles among its squares. In **220**, a given triangle is located two steps horizontally and one step vertically away from its nearest neighbor. Structure **220** is chiral, existing as a pair of enantiomers (Figure 57b). Using graph theory, the authors predicted that Pd^II^_48_L_96_*tet*-G(2,2) structures would be the next largest members of their new series. Meticulous modeling predicted that a ditopic ligand with a bend angle of 152° should favor the formation of such a Pd^II^_48_L_96_ architecture, suggesting that the initially observed Pd^II^_30_**219**_60_ structure is a kinetically trapped species.

Through optimization of the conditions of self-assembly and screening of many crystals, the Fujita group was once more able to use their novel crystallographic methods to identify a Pd^II^_48_**219**_96_ structure with the geometry of a larger *tet*-G(2,2) polyhedron. This remarkable structure demonstrates the power of using the analysis of serendipitous results in the targeting and discovery of new structures. Such structures are among the largest synthetic assemblies known, rivaled only by those incorporating biomolecular building blocks. For example, the Heddle group have used metal-driven assembly of protein subunits to produce large assemblies with unusual structures, such as the snub cube.^305^

**Figure 58:**
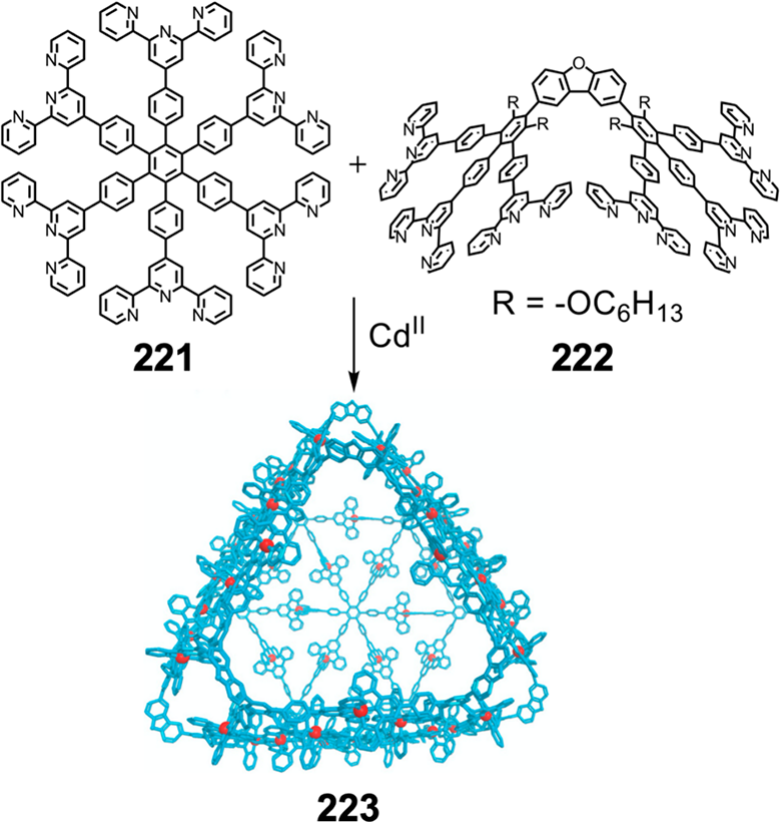
Self-assembly of heteroleptic truncated tetrahedron **223** (energy-minimized structure from molecular modeling) from ligands **221** and **222** together with Cd^II^. Adapted from ref (306). Copyright 2020 American Chemical Society.

Newkome et al. demonstrated the role that geometric constraints can play in the assembly of heteroleptic structures. They reported that mixing hexatopic ligands **221** and **222** with Cd^II^ in a 1:3:12 ratio results in the formation of the Cd^II^_48_**221**_4_**222**_12_ truncated tetrahedral structure **223** with a molecular weight of approximately 70 kDa (Figure 58).^306^ Each of the individual bent **222** ligands acts to connect two hexagonal **221** units, four of which make up the faces of the truncated tetrahedron. The rigidity of bent **222** enables the selective formation of the desired structure, which does not form when a more flexible alkyl linker is used as the spacer between the two tris(terpyridine) units. Instead, double-decker hexagons are formed.^307^

### 7.2 Stretching Ligands: Elongated Tetratopic Ligands

Metal–organic cages with a high degree of enclosure are often targeted, as well-enclosed cavities tend to exhibit superior guest binding properties. However, other classes of supramolecular host, such as cucurbiturils^308^ and pillarenes,^309^ have open-ended structures and have been used in applications where this openness is useful. Control of the degree of enclosure can also affect the guest binding kinetics,^310,311^ which is a vital parameter in designing functional systems. As a result, metal–organic architectures with open-ended box-, barrel-, and tubelike structures have been investigated.

**Figure 59:**
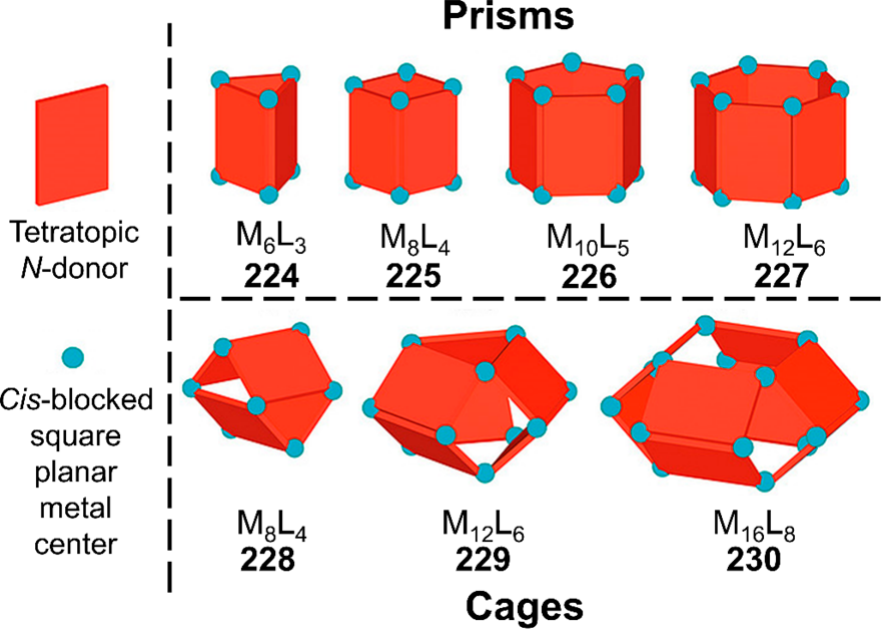
Geometries that can be formed by the combination of elongated tetratopic N-donor ligands and *cis*-protected square-planar metal centers in a 1:2 ratio. Reproduced with permission from ref (325). Copyright 2019 Wiley-VCH Verlag GmbH & Co. KGaA, Weinheim.

The combination of elongated tetratopic pyridyl-based ligands and *cis*-protected square-planar metal centers has been shown to yield a variety of trigonal,^312−314^ tetragonal,^315−318^ pentagonal,^319^ and hexagonal^320^ barrel-like structures (**224**–**227**; Figure 59).^321,322^ Intriguingly, recent reports have demonstrated that ligands of this class can also form structures with gyrobifastigium,^323−325^ triangular-orthobicupola,^326,327^ and square-orthobicupola^319^ geometries (**228**–**230**), in some cases selectively and in others as a minor product. Thus, while the combination of elongated tetrapyridyl ligands with *cis*-protected square-planar metal centers can yield different structures (Figure 59), control over the thermodynamics of the system is required to ensure that a single product is formed.

Recent work by Severin and co-workers sought to uncover the design principles responsible for the assembly of several different structures, particularly taking into account geometric considerations.^319^ When the authors postulated that the ligands were fully rigid, geometric analysis predicted that larger, often pentagonal, barrels would form. However, tetragonal or trigonal-prismatic barrels are observed in practice. The formation of these entropically favored smaller assemblies is thus attributed to the conformational flexibility of the ligands, which enables them to deviate from planarity, and also to flexibility arising from the potential for a slight misalignment between the coordinate vectors of the ligand and the metal–ligand bonds.

Severin’s group also discovered that ligands containing bulky cores can form structures with gyrobifastigium-like geometries (**228**; Figure 59).^323^ This geometry allows a greater distance between bulky ligand cores, thereby reducing steric clashes compared to barrel-like geometries. Further geometric analysis indicated that the gyrobifastigium structure emerges only in a certain window of ligand length-to-width ratios. Further elongation of the ligand precludes gyrobifastigium formation, favoring instead the formation of larger barrel-like assemblies, which also reduce steric clashes between ligand cores.^319^

Factors beyond simple geometric considerations were also revealed to be important in determining which among the many architectures shown in Figure 59 might predominate. For example, favorable interligand non-covalent interactions can influence the preferred geometry of the structures formed.^325^ Although analysis of geometric considerations does not yet provide a definitive guide for selectively obtaining each of the seven structure types shown in Figure 59, these principles may be used to guide the targeting of other structure types, using ligands with bulky metal(II) clathrochelate cores.^319^

**Figure 60:**
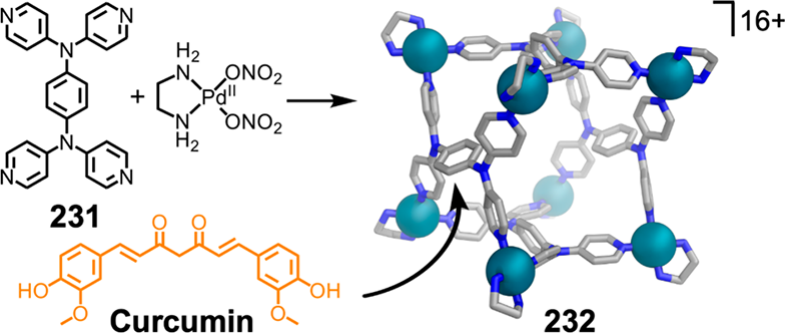
Formation of water-soluble tetrafacial barrel **232** from tetrapyridine **231** and *cis*-Pd^II^(en)(NO_3_)_2_. Barrel **232** increases the water solubility and photostability of curcumin.^315^

Mukherjee et al. further demonstrated the utility of this class of structures using water-soluble tetrafacial barrel **232**, selectively formed via the combination of tetrapyridyl ligand **231** and *cis*-Pd^II^(en)(NO_3_)_2_ in a 1:2 ratio (Figure 60). This barrel acts as a carrier for curcumin.^315^ Complexation within the cavity increases the water solubility of curcumin and also protects it from photodegradation in aqueous solution when it is exposed to either daylight or UV light. The authors attribute the photostabilizing property of the metal–organic barrel to absorption of most of the incident photons by the aromatic panels of the structure, reducing the number absorbed by the curcumin guest. Curcumin has been shown to have pharmacological activity,^328^ but two factors limiting its potential use in therapeutics are its tendency to undergo photodegradation^329^ and its low bioavailability arising from poor aqueous solubility,^330^ both of which may be alleviated by supramolecular carrier **232**. The Mukherjee group also reported a urea-functionalized trigonal prism capable of catalyzing Diels–Alder reactions in aqueous media, further demonstrating the potential application of water-soluble open cages.^331^

Utilizing the principles discussed above, along with those illustrated in Figure 3, Zhang and co-workers constructed heteroleptic tetragonal barrel-shaped metallacages, which were emissive in both solution and the solid state.^332^

**Figure 61:**
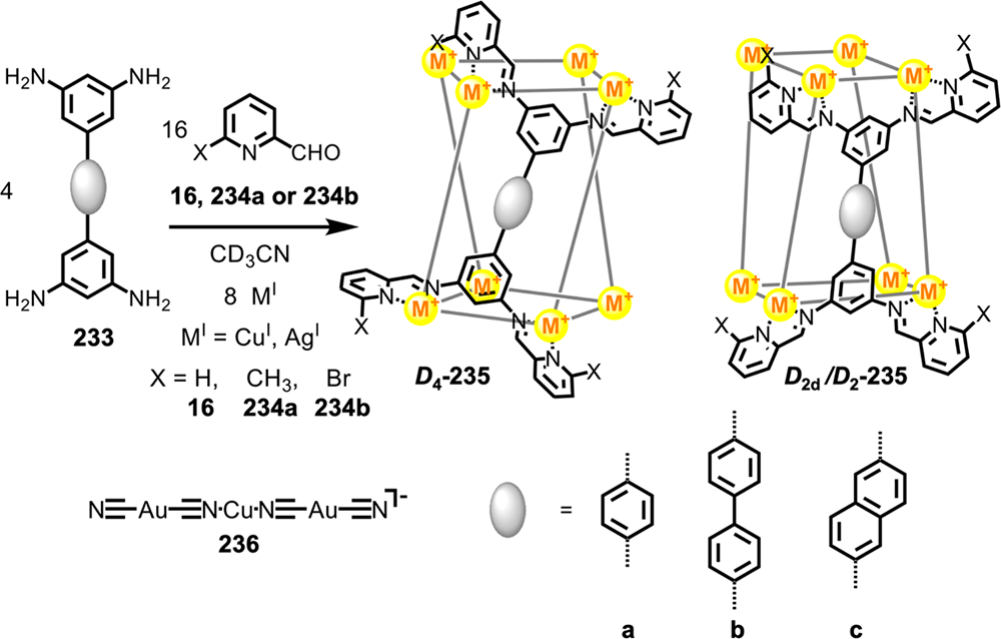
Subcomponent self-assembly of M^I^_8_L_4_ tubelike structures **235** with narrow cavities capable of binding linear cyanoaurate guest complexes such as **236**. Adapted from ref (334). Copyright 2014 American Chemical Society.

We reported the formation of a series of tubular M^I^_8_L_4_ structures **235** via subcomponent self-assembly (Figure 61) that have narrower cavities than the other structures discussed above. They are obtained from the reactions of elongated tetraanilines (**233a**–**c**), a 2-formylpyridine (**16**, **234a**, or **234b**), and Ag^I^ or Cu^I^.^333,334^ The tubelike hosts exist in two possible diastereomeric forms, either *D*_2_/*D*_2*d*_-**235** or *D*_4_-**235**. The equilibria between diastereomers depend upon the ligand length, the substituents, the identities of the metal ion and the counteranion, and the temperature.^334^ Furthermore, the linear cavities of the *D*_4_-symmetric isomers of these structures bind and stabilize unusual cyanide-based linear guest species such as **236**, illustrating an advantage to the formation of elongated cavities.

### 7.3 Curved versus Planar Ligands

Much of section 7.1 focused upon architectures produced from bent ditopic ligands and their analogues. On the basis of similar reasoning, curvature can be introduced into ligands with higher topicities. When they are planar or nearly planar, such ligands have been employed as panels in the formation of diverse architectures with both high and low symmetries.^1,66,335^ The deviation of a ligand from planarity can favor the formation of metal–organic complexes with greater complexity and lower symmetry.

**Figure 62:**
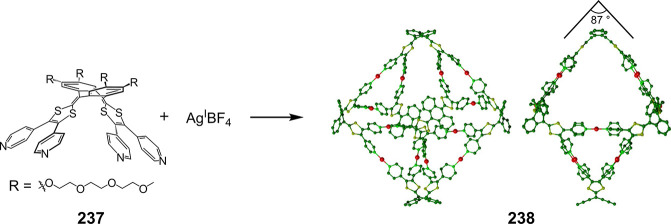
Proposed structure of **238** assembled from ligand **237** and Ag^I^BF_4_. Reproduced with permission from ref (338). Copyright 2018 Wiley-VCH Verlag GmbH & Co. KGaA, Weinheim.

Salle et al. used tetrapyridyl ligands based on π-extended tetrathiafulvalenes (exTTF) to construct M_4_L_2_^336^ and M_6_L_3_^337^ ringlike structures as well as a larger M_12_L_6_^338^ species. The combination of curved tetratopic ligand **237** with Ag^I^BF_4_ in mixed CHCl_3_/CH_3_NO_2_ forms Ag^I^_12_**237**_6_ architecture **238** (Figure 62).^338^ Although an X-ray crystal structure was not obtained for **238**, solution studies and molecular modeling enabled its assignment as shown in Figure 62. The modeled structure has a ligand curvature of 87°, close to the value of 86° observed for the free ligand. An analogous structure forms by the assembly of ligand **237** with *trans*-Pd^II^Cl_2_(MeCN)_2_ in DMSO.^338^

An additional feature of electron-rich ligand **237** is its ability to undergo two-electron oxidation. The conformational change of the ligand core that accompanies this oxidation was exploited to drive the redox-controlled disassembly and reassembly of a M_4_L_2_ coordination cage. This redox-governed process was coupled with guest binding to provide a means of guest release and recapture.^339^ Ag^I^_12_**237**_6_ structure **238** rearranges from the discrete cage into a three-dimensional supramolecular polymer when the ligand units are oxidized to the dicationic state. In contrast, the Pd^II^_12_**237**_6_ cage remains intact upon oxidation.^338^

### 7.4 Linear Polytopic Ligands

The development of structures that can bind more than one guest is of great interest in the context of new coordination cage applications.^57^ This goal may be achieved through the design and synthesis of metallosupramolecular structures that contain multiple linked cavities with a high degree of enclosure and are therefore expected to exhibit guest binding. Crowley and co-workers expanded on design principles for the formation of simpler Pd^II^_2_L_4_ structures to design pseudolinear polypyridyl ligands that form multicavity structures.^93^ These architectures contain similar cavities linked end to end.

**Figure 63:**
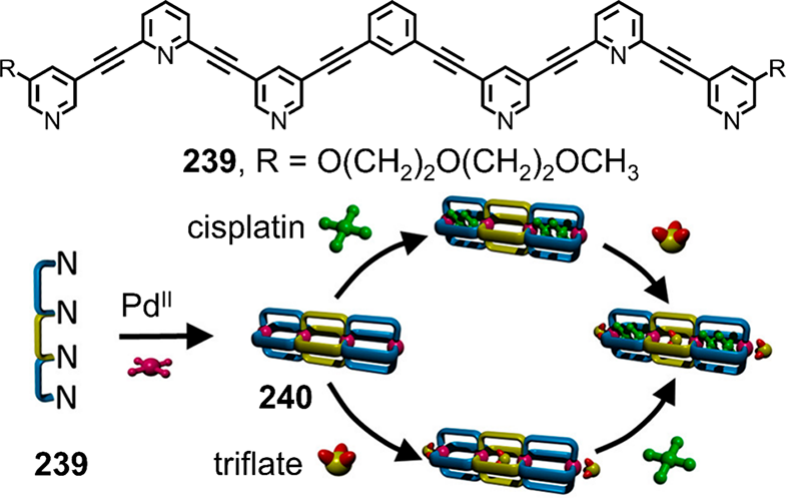
Self-assembly of ligand **239** with Pd^II^ into triple-cavity cage **240**, which is capable of binding two different types of guest molecules within two distinct types of internal cavities. Reproduced from ref (93). Copyright 2017 American Chemical Society.

As shown in Figure 63, combining hexapyridyl ligand **239** and Pd^II^ leads to the formation of triple-cavity cage **240**. A higher temperature than typically needed for the formation of Pd^II^_2_L_4_ complexes is required in order for the error-correcting disassembly of misassembled intermediate structures to occur during the formation of multicavity structures. To illustrate the potential use of such cages, the authors demonstrated the segregated binding of two distinct types of guests within the two different cavity types (terminal and central) within **240**. Cisplatin is encapsulated in the terminal cavities, whereas triflate is bound in the central cavity as well as externally at each end of the structure.^93^

Clever et al. formed double- and triple-cavity cages by expanding upon these principles. A topologically interpenetrated dimeric species forms from the double-cavity cage upon addition of chloride or bromide ions.^340^ Each of the five segregated interior cavities of the structure can bind chloride or bromide.

**Figure 64:**
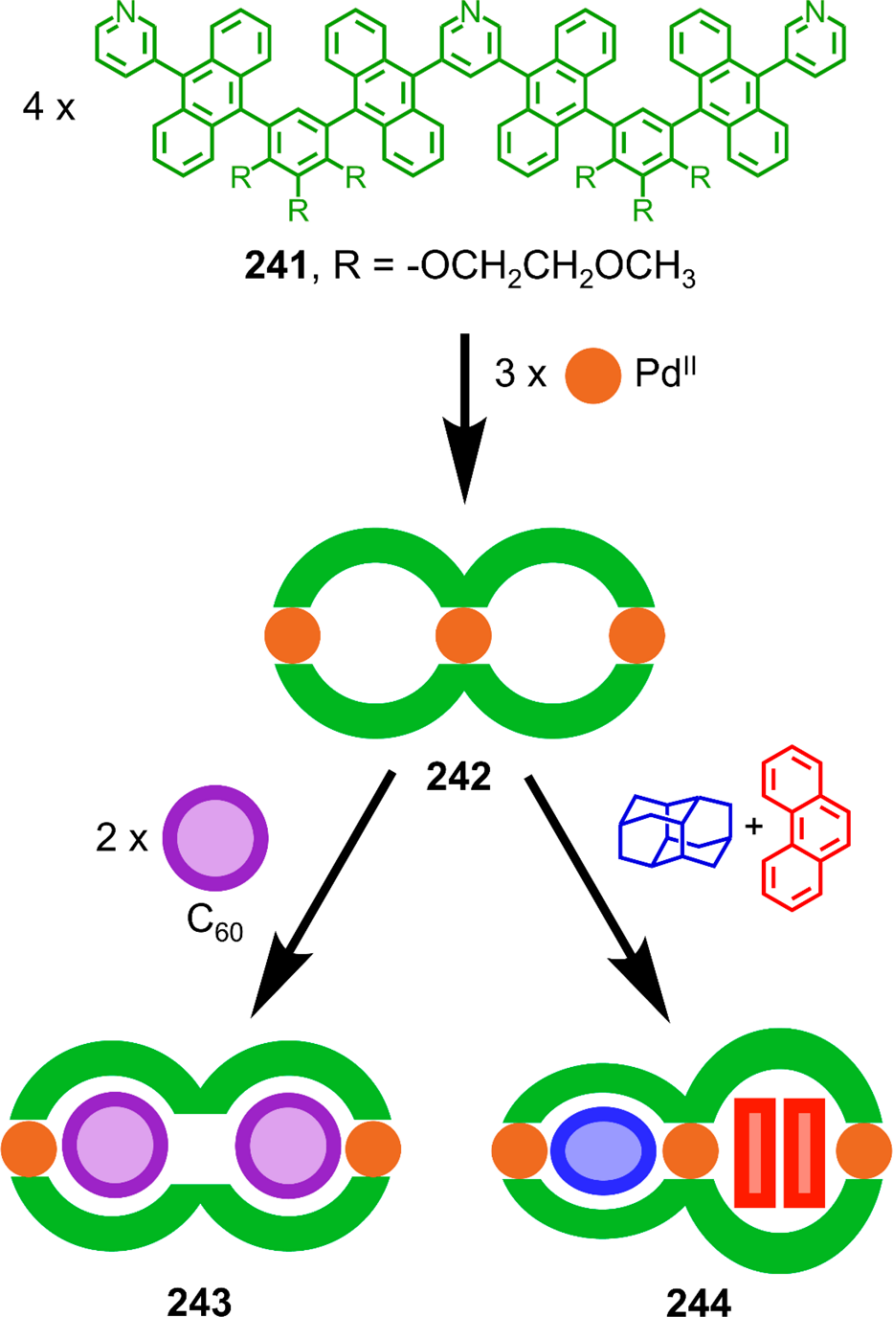
Molecular double capsule **242**, which can form “molecular peanut” **243** upon ejection of the central Pd^II^ ion and encapsulation of two C_60_ molecules or heterotopic (diamantane)(phenanthrene)_2_ complex **244**.^341^

Yoshizawa et al. reported the assembly of W-shaped tripyridine ligand **241** with Pd^II^ to form Pd^II^_3_**241**_4_ double capsule **242**.^341^ When structure **242** is heated with C_60_, the central Pd^II^ is ejected, resulting in the formation of Pd^II^_2_**241**_4_·(C_60_)_2_ “molecular peanut” **243** (Figure 64), with extensive stabilization from aromatic stacking interactions between the anthracene panels of the host and the fullerene guests outweighing the energetic cost associated with a loss of coordinative saturation of the central pyridine. Capsule **242** also simultaneously binds different guests, diamantane and phenanthrene, with the preference for heterotopic encapsulation of these guests to form **242**·(diamantane)(phenanthrene)_2_ complex **244** attributed to cooperative changes in the volumes of the two cavities that occur upon guest binding. The same group later showed that similar peanut-shaped polyaromatic shells form under much milder conditions when the central pyridine ring is replaced by a phenyl ring, which allows the stepwise encapsulation of two C_60_ molecules.^342^

**Figure 65:**
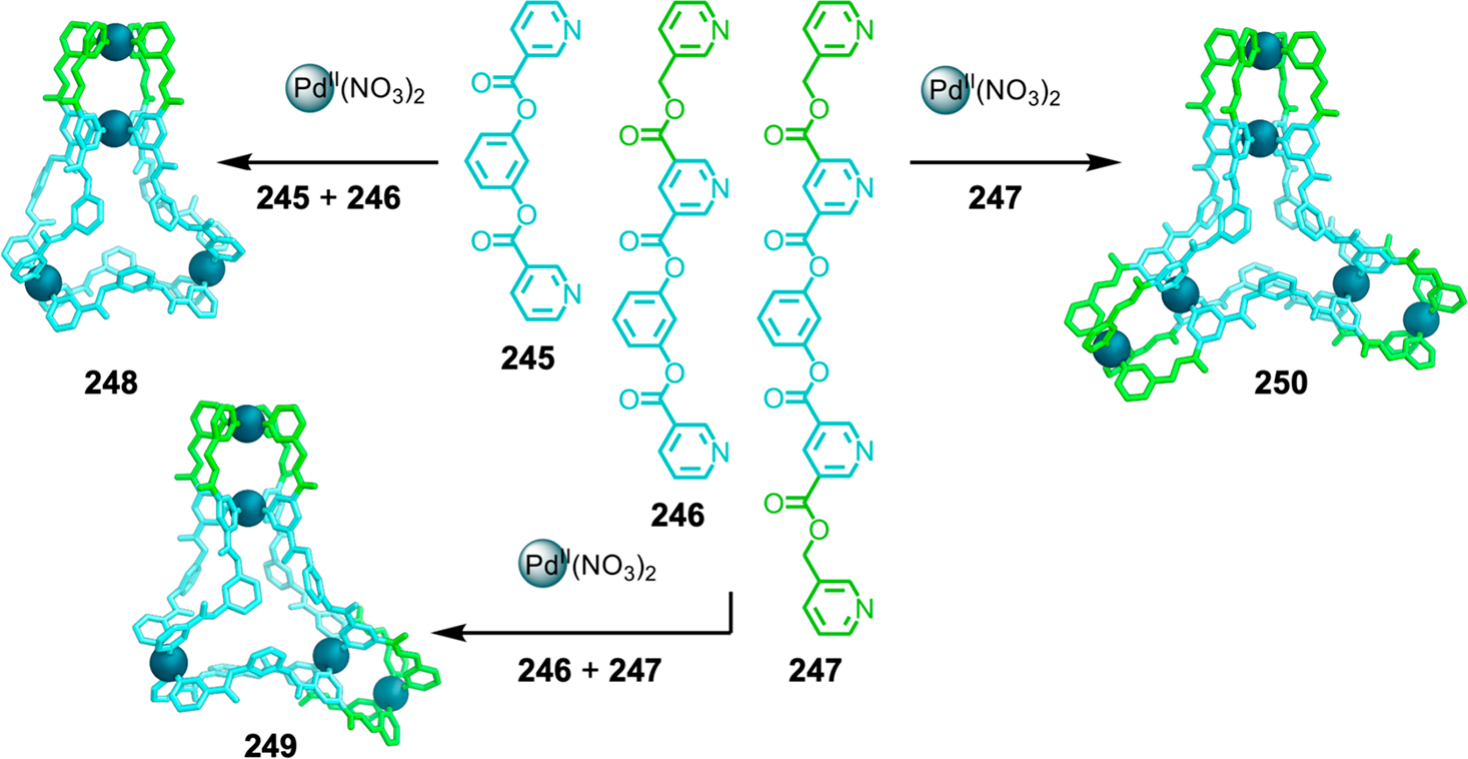
Conjoined cages **248**–**250** consisting of laterally or linearly conjoined Pd^II^_2_L_4_ and Pd^II^_3_L_6_ units; heteroleptic **248** and **249** form selectively via integrative self-sorting of mixtures of the corresponding ligands.^343^

Chand and co-workers reported that ligands **245**–**247** form the conjoined cages **248**–**250** (Figure 65), further developing the concepts introduced above. Instead of different cavities having similar sizes and shapes, ligands were rationally designed to form architectures consisting of laterally or linearly conjoined Pd^II^_2_L_4_ and Pd^II^_3_L_6_ units (Figure 65).^343^ Structures **248**–**250**, which were all confirmed by X-ray crystallography, assemble from one or more of the carefully designed ligands **245**–**247** and Pd^II^ in DMSO. In the cases of **248** and **249**, integrative self-sorting results in the selective formation of heteroleptic structures. The different types of cavities bind different guests. Small anions, such as NO_3_^–^ and Cl^–^, bind within the smaller cavities, and their presence is required to template the formation of structures **248**–**250**. Crystallography also showed that multiple DMSO molecules reside in the larger, trigonal, cavity.

**Figure 66:**
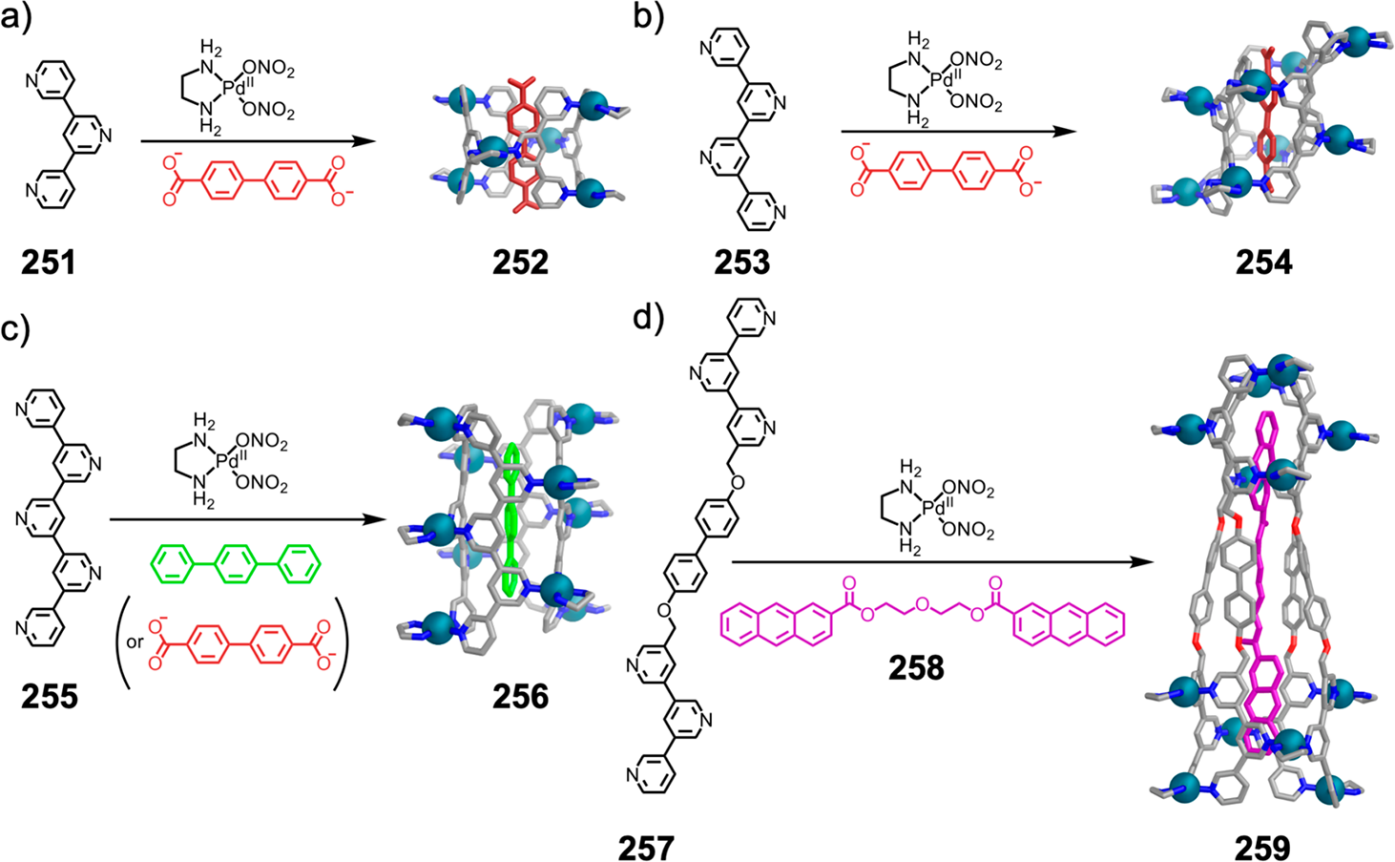
(a) Pd^II^_6_**251**_4_ (**252**), (b) Pd^II^_8_**253**_4_ (**254**), (c) Pd^II^_10_**255**_4_ (**256**), and (d) Pd^II^_12_**257**_4_ (**259**) nanotubes prepared through assembly of the given ligands with *cis*-protected Pd^II^ centers and a rodlike template.^344−346^

Fujita et al. reported the use of linear polypyridyl ligands in the formation of a series of nanotubular architectures. The combination of ligand **251** (Figure 66a) with *cis*-protected Pd^II^ and a template such as 4,4′-biphenyldicarboxylate leads to the formation of Pd^II^_6_L_4_**252**; elongated ligands **253** (Figure 66b) and **255** (Figure 66c) lead correspondingly to Pd^II^_8_L_4_**254** and Pd^II^_10_L_4_**256**. A suitable template is essential to generate well-defined, discrete assemblies.^344^ The Pd^II^_6_**251**_4_ and Pd^II^_10_**255**_4_ nanotubes, **252** and **256**, respectively, exist as single isomers. However, the Pd^II^_8_**253**_4_ structure forms as a mixture of isomers having *C*_2*h*_ (**254**) and *D*_2*h*_ symmetries.^345^

Later work showed that elongation of these tubelike structures through extension of the ligand with additional pyridine rings was not possible because of poor solubility.^346^ This problem was circumvented by the design of hexapyridine ligand **257** (Figure 66d) in which two terpyridine units are separated by a spacer. Importantly, the biphenyl spacer, as opposed to an alkyl moiety, ensured that the ligand remained linear instead of folding into a U-shaped conformation. Combination of **257** with *cis*-protected Pd^II^ and the specially designed template molecule **258** allowed the construction of 3.5 nm long nanotube **259**.

### 7.5 Pyrimidine versus Pyridine

The combination of pyridine donors with *cis*-protected square-planar metal centers has yielded many new coordination cages, some of which are described in this review. Pyrimidine donors are also able to coordinate to M^II^ centers, acting as 120° μ_2_-bridging ligands.

**Figure 67:**
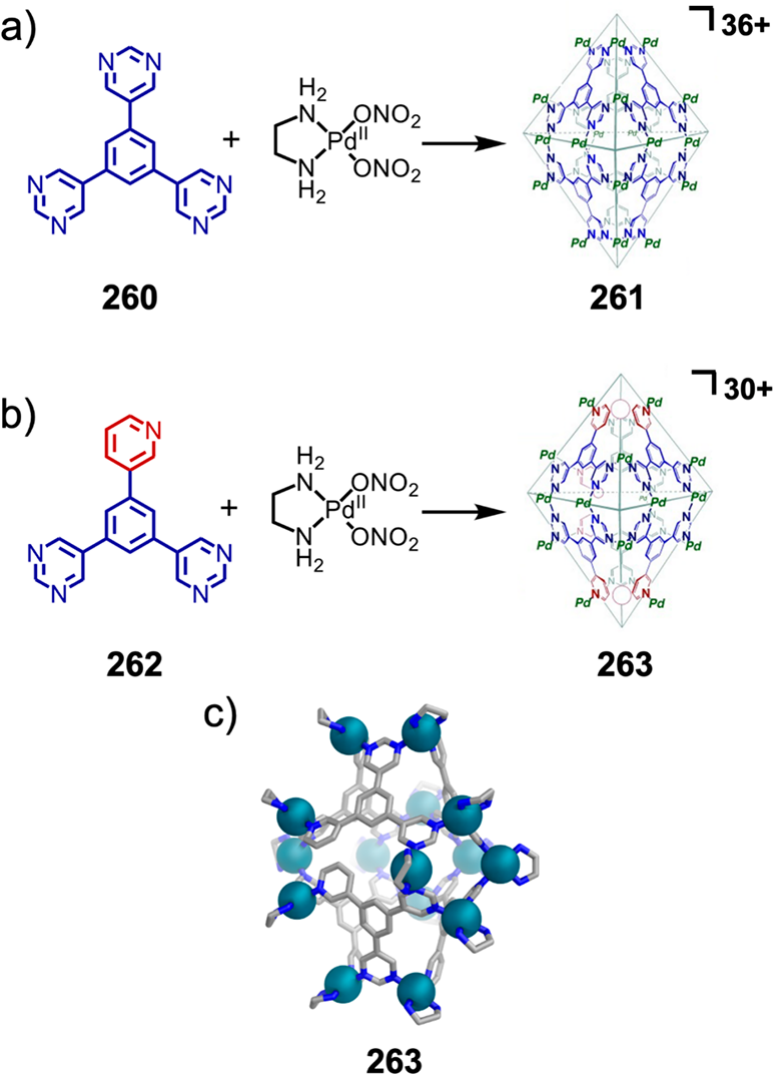
Preparation of (a) Pd^II^_18_**260**_6_ (**261**) and (b) Pd^II^_15_**262**_6_ (**263**) hexahedral architectures from ligands **260** and **262**, respectively, and Pd^II^(en)(NO_3_)_2_. Adapted with permission from ref (348). Copyright 2001 Wiley-VCH Verlag GmbH & Co. KGaA, Weinheim.

Triangular hexadentate 1,3,5-tris(3,5-pyrimidyl)benzene (**260**) is designed to form structures in which the metal centers lie upon the edges of polyhedra as opposed to their vertices.^347^ The combination of **260** with Pd^II^(en)(NO_3_)_2_ in aqueous solution yields enclosed Pd^II^_18_**260**_6_ hexahedron **261** with a trigonal-bipyramidal structure consisting of six edge-sharing triangular panels with two metal centers on each edge (Figure 67a).

Subsequent work produced the similar Pd^II^_15_**262**_6_ hexahedron **263** (Figure 67b) from modified ligand **262**, in which one pyrimidine is replaced with a 3-pyridyl moiety.^348^ Although **261** and **263** are of similar shape and size, the presence of open clefts at the apical nonbinding sites in **263** is hypothesized to allow easier access of guest molecules into the cavity for binding, in contrast to the more enclosed **261**.

Architectures **261** and **263** demonstrate how the geometric constraints of a ligand can result in the formation of novel structures. Subsequent alterations can then be made that allow for a desired structure type to be maintained, but with altered properties. Further replacement of the pyrimidine sites of **262** with 3-pyridyl moieties yields different architectures of both higher and lower symmetries, including a tetrahedron and “open cones”, depending on the conditions used.^349^

**Figure 68:**
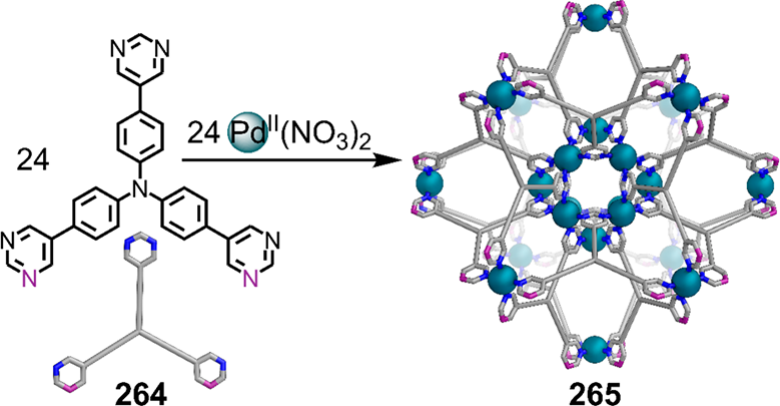
Pd^II^_24_**264**_24_ “pregnant molecular nanoball” **265**. N-donor atoms highlighted in purple remain uncoordinated in the observed product structure.^351^

Mukherjee et al. have also used ligands containing pyrimidine moieties to prepare complex architectures. The combination of 1,4-bis(pyrimidin-5-yl)benzene or 4,4′-bis(pyrimidin-5-yl)biphenyl with *cis*-[(dch)Pt^II^(NO_3_)_2_] (dch = 1,2-diaminocyclohexane) in water results in the formation of Pt^II^_8_L_4_ nanotubes with lengths of up to 22.0 Å.^350^ Assembly of hexadentate ligand **264** (Figure 68) with Pd^II^ in a 1:1 ratio produces structure **265**.^351^ Crystallography indicates that **265** is a discrete Pd^II^_24_**264**_24_ complex wherein only four of the nitrogen atoms on each ligand bind to Pd^II^, leaving two uncoordinated nitrogen atoms on each ligand (highlighted in purple in Figure 68). The authors described this structure as a “pregnant molecular nanoball”, consisting of a Pd^II^_12_ “baby ball” within a larger Pd^II^_12_ “mother ball”. Interestingly, the “mother ball” stabilizes the internal, smaller, structure, as an analogous Pd^II^_12_L_24_ cuboctahedral nanosphere does not form from the reaction between pyrimidine and Pd^II^.

### 7.6 Flexible Coordination Geometry of Metal Building Blocks

Many of the examples in previous sections utilize metal ions with rigid coordination spheres. For example, many structures contain Pd^II^ or first-row transition metal ions in the +2 oxidation state, adopting exclusively square-planar and pseudo-octahedral coordination geometries, respectively. Ions of the f-block metals can more readily adopt a wider range of coordination numbers and geometries, which can result in the formation of complex metal–organic architectures.

**Figure 69:**
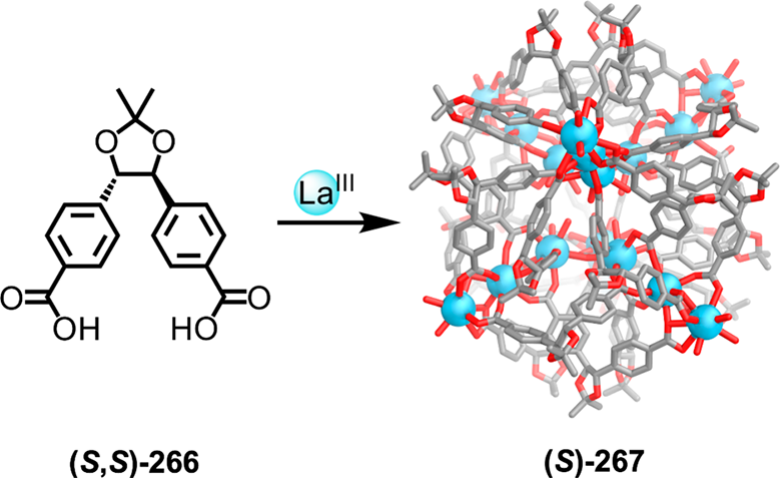
[La_18_**266**_24_(CO_3_)_2_(H_2_O)_32_]^2+^, “lanthanitin”.^352^

Jeong et al. utilized the coordinative flexibility of lanthanum to assemble the complex [La^III^_18_**266**_24_(CO_3_)_2_(H_2_O)_32_]^2+^ structure **267** (Figure 69), which the authors called lanthanitin because of its similarity to the structure of ferritin.^352^ Crystals of both (*S*)- and (*R*)-lanthanitin are obtained from mixtures of La^III^Cl_3_ and the rigid, bent ligand **266** having either (*S*,*S*) or (*R*,*R*) stereochemistry. Within **267**, La^III^ ions have different coordination numbers; a mixture of eight- and 10-coordinate La^III^ is observed.

Duan and co-workers used a different f-block ion, cerium(IV), to construct a Ce^IV^_4_L_6_ basketlike tetragon, using ditopic ligands consisting of two tridentate binding sites connected by a carbazole-based core.^353^ Four Ce^IV^ centers define the four corners of a square and are bridged by four edge-defining ligands. Each of the final two ligands also binds to two of the four cerium ions, defining the bottom of the basket.

Xu and Raymond exploited the variable coordination geometry of lanthanum to form a La^III^_8_L_8_ structure with a square-antiprismatic geometry.^354^ Within the structure, all of the lanthanum centers are nine-coordinate; however, two different coordination geometries are observed: distorted monocapped square-antiprismatic and distorted tricapped trigonal-prismatic. Similarly, Kawai et al. recently reported an octanuclear circular helicate with a *D*_4_-symmetric square-antiprismatic geometry that exhibited circularly polarized luminescence (CPL) activity.^355^

### 7.7 Non-covalent Interactions and Steric Effects

Previous sections have alluded to the importance of steric control and favorable non-covalent interactions in driving the selective formation of particular structures. In this section we present a few key examples in which these often subtle effects exert a decisive influence on the self-assembly process.

**Figure 70:**
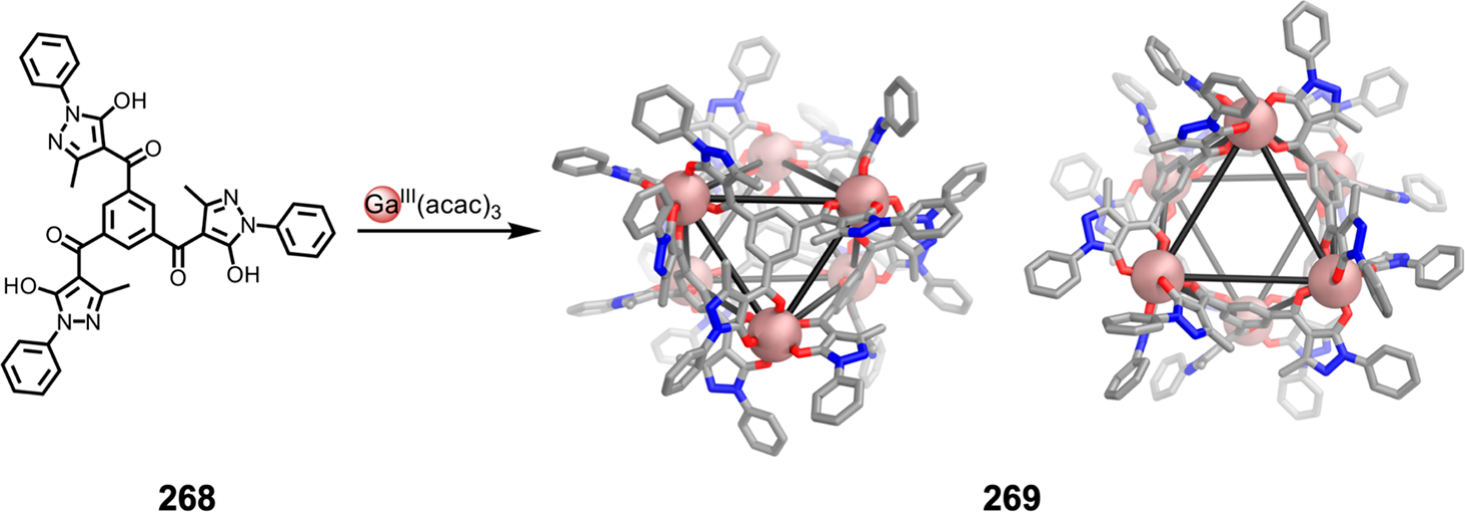
Ga^III^_6_L_6_ distorted trigonal antiprism **269** assembles from rigid, threefold-symmetric, tris-bidentate ligand **268** and gallium(III) in DMSO.^356^

Saalfrank et al. reported an early example wherein steric effects determine self-assembly outcomes.^356^ The combination of rigid, threefold-symmetric, tris-bidentate pyrazolone-based ligand **268** with gallium(III) acetylacetonate in DMSO resulted in the serendipitous formation of Ga^III^_6_**268**_6_ distorted trigonal-antiprismatic cylinder **269** having *D*_3_ symmetry, in which all six gallium centers have the same handedness (Figure 70). Although this structure represents an initially surprising deviation from the expected M_4_L_4_ tetrahedral assembly, molecular modeling indicated that the shorter metal–metal distance in a putative M_4_L_4_ tetrahedron would cause an increase in unfavorable steric clashes. Further work by the same group utilized molecular modeling to clarify the steric preference for the formation of an Fe^III^_4_L_4_ structure in the case of one ligand versus an Fe^III^_6_L_6_ trigonal antiprism with a different ligand on the basis of favorable aromatic stacking interactions in the trigonal antiprism.^357^

**Figure 71:**
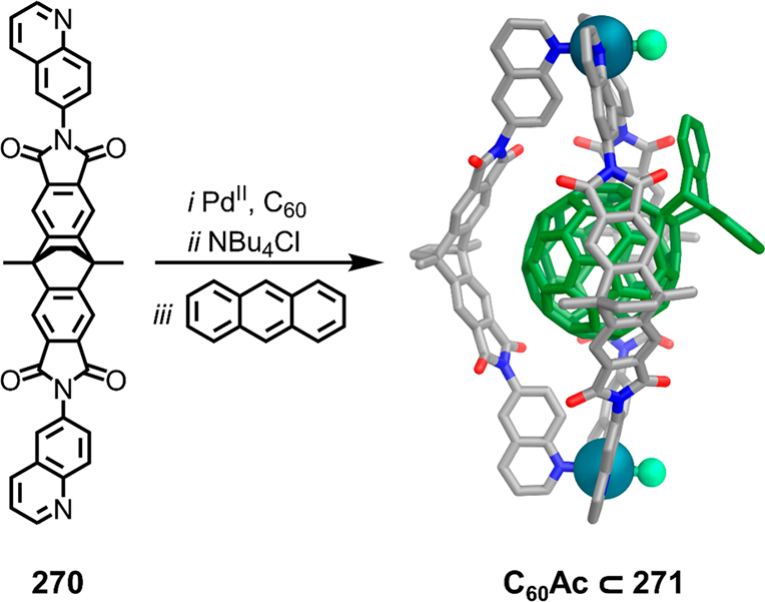
Pd^II^_2_L_3_ bowl **271** is formed, rather than a Pd^II^_2_**270**_4_ cage, due to the steric demands of quinoline-based ligand **270**. Bowl **271** acts as a supramolecular protecting group, enabling the selective formation of a C_60_–anthracene monoadduct (C_60_Ac).^358^

Clever and co-workers described the assembly of Pd^II^_2_**270**_3_ bowl **271** (Figure 71) based on a Pd^II^_2_L_4_ cage framework lacking a fourth ligand.^358^ The formation of **271** is driven by the steric demands of its ligands. In the case of ligand **270**, steric clash between hydrogen atoms near the coordinating nitrogen of the quinoline is alleviated in a Pd^II^_2_**270**_3_ structure, stabilizing **271** with respect to a Pd^II^_2_**270**_4_ structure. Upon prolonged heating of a solution of the Pd^II^_2_**270**_3_ structure, partial conversion to the Pd^II^_2_**270**_4_ structure was observed. However, this conversion could be prevented by the binding of C_60_ in the open cavity of the bowl.

Bowl **271** can also act as a supramolecular protecting group. When a solution of [C_60_⊂Pd^II^_2_**270**_3_Cl_2_]^2+^ is treated with 10 equiv of anthracene, the C_60_–anthracene monoadduct (C_60_Ac) is selectively formed without undergoing further reaction with anthracene. As shown in Figure 71, the bowl-like cavity of **271** encloses most of the surface of the C_60_ guest, leaving only a small region available for reaction with anthracene. Finally, a pill-shaped dimer can be formed by bridging the bowls of 2 equiv of **271** with a sterically undemanding terephthalate unit,^358^ utilizing principles similar to those outlined in section 2.1. The example described above (Figure 71) demonstrates that introducing steric bulk close to the coordinating sites of ligands can increase their propensity to form more complex structures.

**Figure 72:**
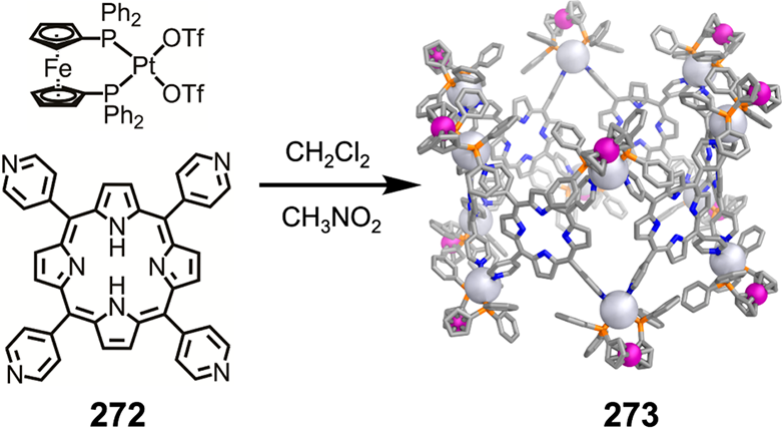
Formation of multimetallic porphyrin-based open hexagonal box **273** from tetrapyridylporphyrin **272**.^359^

Mukherjee and co-workers reported the reaction of (1,1′-bis(diphenylphosphino)ferrocene)platinum(II) with 5,10,15,20-tetrakis(4-pyridyl)porphyrin (**272**) ligands to form open hexagonal box **273** (Figure 72), as opposed to a more symmetric cubic architecture.^359^ The bulky ferrocene-derived diphosphine ligand disfavors cube formation and allows formation of hexagonal box **273** with an internal cavity of 43 550 Å^3^. The formation of **273** depends on the introduction of steric bulk in peripheral coordinating ligands rather than directly affecting ligand–metal binding. This unusual self-assembled architecture senses the presence of zinc(II), with distinct changes in UV/vis absorbance bands observed in methanol solution caused by metalation of the porphyrins.

**Figure 73:**
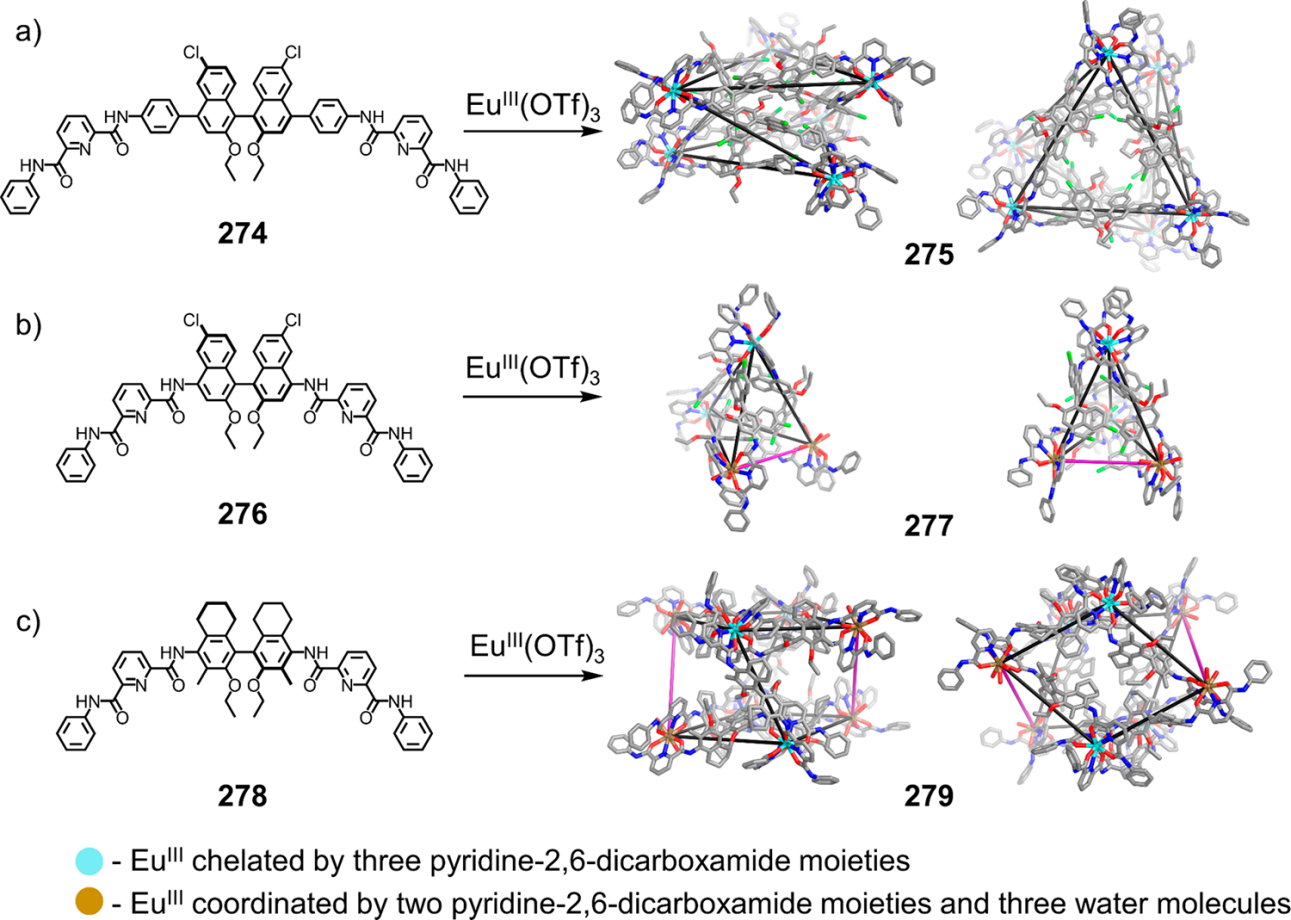
Assembly of ligands **274**, **276**, and **278** with Eu^III^ formed (a) twisted triangular prism **275**, (b) “defective” tetrahedron **277** with one missing edge, and (c) “defective” hexahedron **279** with two edges missing. The “missing” edges in the structures are indicated with purple struts.^360^

Liu and co-workers built metal–organic architectures with complex structures using Eu^III^. The ditopic ligands **274**, **276**, and **278**, each with two pyridine-2,6-dicarboxamide tridentate chelating sites connected by a 1,1′-bi-2-naphthol-derived core, produce architectures **275**, **277**, and **279**, respectively (Figure 73).^360^ The key difference between the three ligands is the amount of steric bulk in proximity to the tridentate binding sites. All of the Eu^III^ centers within the three structures are nine-coordinate. In **275**, each of the six Eu^III^ centers is chelated by three pyridine-2,6-dicarboxamide moieties, resulting in a structure with a twisted triangular-prismatic geometry (Figure 73a).

Because of the increased steric hindrance near the binding sites in ligands **276** and **278**, however, structures **277** and **279** contain Eu^III^ centers that are chelated by only two pyridine-2,6-dicarboxamide units, with the remaining coordination sites on Eu^III^ occupied by water ligands. The result is the formation of “defective” cages **277** and **279** (Figure 73b,c). Eu^III^_4_**276**_5_(H_2_O)_6_(OTf)_12_ architecture **277** has a structure approximating a distorted tetrahedron with one missing edge (Figure 73b). In contrast, Eu^III^_8_**278**_10_(H_2_O)_12_(OTf_2_)_24_ structure **279** resembles a hexahedral cage in which two edges are missing (Figure 73c).

We also utilized steric effects and non-covalent interactions to drive the formation of barrel-like prismatic structures instead of simple tetrahedra.^229^ Enforcing *mer* selectivity at the metal centers on the basis of reduced steric crowding and increased interligand aromatic stacking interactions enabled the formation of M_8_L_12_, M_10_L_15_, and M_12_L_18_ prismatic barrel structures using fluorinated ligands. We hypothesize that the presence of favorable edge-to-face aromatic interactions between the triazine and phenanthroline moieties of neighboring ligands contributed to the stabilization of an unusual M_6_L_4_*S*_4_-symmetric scalenohedron over a higher-symmetry pseudo-octahedral structure.^361^ Finally, with Siegel and Baldridge we reported the assembly of an *S*_10_-symmetric, fivefold-interlocked [2]catenane from Cu^I^, a corannulene-based pentaaniline, and 2-formyl-6-methylpyridine.^362^ DFT calculations indicated that interligand aromatic interactions between corannulene units are a key driving force for the interlocking of the two fivefold-symmetric cages.

This section highlights the critical role of geometric and steric factors, as well as the enhancement of non-covalent interactions, in controlling the final structure observed in self-assembly processes. Although a sizable amount of work has already been conducted in this area, these examples also illustrate that much space remains to be explored. Fujita’s contributions, especially those involving the use of bis-monodentate ligands with Pd^II^ metal centers, may inspire similar systematic studies focusing on small changes to one geometric feature of other classes of ligand, thereby leading to the discovery of general design principles for complex architectures.

## 8 Conclusion and Outlook

As we have detailed in this review, recent years have seen the rapid development of many new approaches to the construction of metal–organic structures beyond the Platonic solids. The advent of new applications for supramolecular cages, including in catalysis,^363−369^ sensing,^370−372^ molecular separations,^373^ and biology,^374^ has provided strong motivation for this development.

As detailed in section 2, incorporating multiple different building blocks into the same structure is an effective way of increasing complexity. However, ensuring that mixed-component structures are formed selectively and that narcissistic sorting is prevented remains challenging. The six methods we outlined for driving heteroleptic architecture formation are variations on the theme of careful ligand selection and pairing. Recent efforts have demonstrated the reliability of heteroleptic approaches for the assembly of coordination cages, producing targeted structures across a range of ligand classes.^62,73,82,83,87,89,90,96,107,128,375^

Similarly, as noted in section 6, an understanding of ligand coordination preferences can now allow the rational design of new heterometallic architectures. Employing metal ions that form coordination bonds to ligands with differing kinetic lability also provides a useful method of design. However, other methods of producing multimetallic architectures have been less thoroughly investigated, providing scope for future enquiry.

The principles of ligand flexibility, solvent effects, and templation detailed in sections 4 and 5 are well-established, and many early examples of complex architectures depend on these approaches. They can be unpredictable, however, and although serendipitous results are plentiful, targeted design from first principles remains a challenge. The factors that drive the formation of particular structures can be difficult to decipher, even after discovery. Key exceptions include groundbreaking work by Ward et al. involving thorough investigations of the self-assembly processes of flexible di- and tritopic pyrazolylpyridine-based ligands and octahedral metal centers, which led to several new classes of structures.^186−196^

The reduced-symmetry-ligand and geometry-analysis approaches of sections 3 and 7 have benefited greatly from recent advances. As with heteroleptic approaches, it appears intuitive that reducing the symmetry of a ligand or building block should result in a self-assembled structure of reduced symmetry or increased complexity. However, experience has shown that it can be challenging to obtain single structures as opposed to intractable mixtures. Recent work has nonetheless clarified the circumstances under which a reduction in the symmetry of ligands can result in the formation of a single, complex architecture as opposed to many of them.

Finally, we note that computational methods, including evolutionary algorithms^376^ and machine learning,^377^ are playing an increasing role in the discovery of new supramolecular cage structures^378−380^ and the rationalization of their applications, such as catalytic activity.^381^ The recent implementation of high-throughput synthetic screening using automation has also vastly increased the capacity for rapid exploration of large chemical spaces.^382−384^ The advent of artificial intelligence, in particular machine learning, and automated synthetic methods may thus play a key role in the structural prediction and subsequent synthesis of a broad range of low-symmetry metal–organic polyhedral capsules.
